# Identification of tumorigenesis-related mRNAs associated with RNA-binding protein HuR in thyroid cancer cells

**DOI:** 10.18632/oncotarget.11255

**Published:** 2016-08-12

**Authors:** Federica Baldan, Catia Mio, Lorenzo Allegri, Ketty Conzatti, Barbara Toffoletto, Cinzia Puppin, Slobodanka Radovic, Carlo Vascotto, Diego Russo, Carla Di Loreto, Giuseppe Damante

**Affiliations:** ^1^ Department of Medical and Biological Sciences, University of Udine, 33100 Udine, Italy; ^2^ IGA Technology Services Srl, 33100 Udine, Italy; ^3^ Department of Health Sciences, University of Catanzaro “Magna Graecia”, 88100 Catanzaro, Italy; ^4^ Institute of Anatomic Pathology, University Hospital “S. Maria della Misericordia”, 33100 Udine, Italy; ^5^ Institute of Medical Genetics, University Hospital “S. Maria della Misericordia”, 33100 Udine, Italy

**Keywords:** HuR, RNA-binding proteins, RNA-seq, RIP-seq

## Abstract

RNA binding proteins (RBPs) play a central role in cell physiology and pathology. Among them, HuR is a nuclear RBP, which shuttles to the cytoplasm to allow its RNA targets processing. HuR over-expression and delocalization are often associated to cell transformation. Numerous cancers display increased HuR protein levels and its high cytoplasmic levels has been associated with a worse prognosis.

In our study, we first evaluated HuR expression in normal and cancer thyroid tissues and then evaluated its function in thyroid cell lines. HuR is over-expressed in all thyroid tumor tissues; high cytoplasmic levels are detected in all thyroid carcinomas. HuR silencing decreased cell viability and determined apoptotic cell death, in a non-tumorigenic (Nthy-ori-3.1) and a tumorigenic (BCPAP) thyroid cell line. Global transcriptome analysis indicated that HuR silencing, though having similar biological effects, induces distinct gene expression modifications in the two cell lines. By using the RIP-seq approach, the HuR-bound RNA profiles of different thyroid cell lines were evaluated. We show that in distinct cell lines HuR-bound RNA profiles are different. A set of 114 HuR-bound RNAs distinguishing tumorigenic cell lines from the non-tumorigenic one was identified.

Altogether, our data indicate that HuR plays a role in thyroid tumorigenesis. Moreover, our findings are a proof of concept that RBP targets differ between cells with the same origin but with distinct biological behavior.

## INTRODUCTION

Gene expression regulation is an essential process by which cells react to microenvironment changes. One of the key mechanisms of gene expression regulation occurs at post-transcriptional levels, i.e on mRNA [1]. RNA regulation allows cells to react to environmental stimuli more quickly than *de novo* transcription. In fact, many important cellular processes, such as proliferation, differentiation and apoptosis, are regulated by post-transcriptional mechanisms controlling RNA stability, localization and translation [2].

In eukaryotic cells, RNAs is associated with RNA-binding proteins (RBPs), a protein family that can bind single or double stranded RNA to form ribonucleoprotein complexes (RNPs) [3]. RBPs regulate all phases of RNA biogenesis, including splicing, capping, 3′ end formation, nucleocytoplasmic transport, localization, translation and degradation [4].

RBPs bind their targets in particular sequences or to specific secondary structures, located especially in the untranslated regions (UTRs). The specific bound between regulatory proteins and these elements is achieved by RNA-binding domains (RBDs) [4]. Currently more than 40 RBDs have been identified and, if we consider that a RBP can contain one or, more often, various combinations of different RBDs, it is not that hard to understand the high flexibility of the interaction with different targets [3, 4].

Alteration in RBP activities or RBP-targets interactions could be damaging for gene expression regulation [5]. Moreover, an aberrant RBP expression has been unveil in several diseases, such as muscular atrophies, neurological disorders and cancer [6]. Indeed, in many neoplasia, it has been described an altered expression of several RBPs, which act by changing their binding to tumor tissue-specific oncogenes or tumor suppressors [4].

The human embryonic lethal abnormal vision-like protein (ELAVL1 or HuR) is a member of the Hu family of RNA-binding proteins and is one of the most remarkable RBP known to be implicated in tumorigenesis [4, 7, 8].

HuR binds its mRNA targets through its *RNA recognition motifs* (RRMs) which recognize sequences rich in adenosine/uridine or uridine (AREs), that are mostly localized in the transcript non-coding regions, such as introns and the 3′ untranslated region [9]. In physiological conditions, HuR is located into the nucleus, but can shuttle to the cytoplasm to allow its mRNA target to be processed. Since transcripts coding for important tumorigenesis factors, oncogenes, growth and anti-apoptotic factors are described among HuR targets, it is not surprising that an aberrant over-expression of HuR is associated to cellular transformation [8, 9]. Indeed, heightened HuR protein levels have been observed in numerous cancers [4]. Furthermore, high HuR cytoplasmic levels are known to be associated with a worse prognosis in many tumor types, including lung adenocarcinoma, gall bladder carcinoma, urothelial carcinoma, ovarian cancer, breast cancer, laryngeal squamous cell cancer and colon cancer [8–10].

Thyroid cancer is the most frequent endocrine neoplasm and its incidence has been enhanced in the last decade [11]. Most of thyroid carcinomas derived from follicular cells are classified in papillary, follicular and anaplastic thyroid cancer (PTC, FTC, and ATC, respectively). PTC and FTC maintains a certain degree of differentiation and are also named differentiated thyroid carcinomas (DTC) [12]. Although most of DTCs has a favorable outcome, some of them show an aggressive behavior [13]. Nowadays there are not molecular markers able to efficiently discriminate DTC with different aggressiveness.

In this study, we assessed HuR levels in human thyroid tissues and cell lines, comparing normal and cancer samples. We demonstrated a general HuR overexpression in thyroid tumors and that cytoplasmic HuR staining could discriminates malignant from benign lesions. We then investigated the effects of HuR silencing in thyroid tumor and non-cancerous cell lines, evaluating cell viability and global gene expression profiles modification. Finally, we delineated the HuR-bound RNA profiles in different thyroid cell lines. Altogether, our data provide the proof of principle that investigation on RNA binding proteins could provide clues for understanding of mechanisms underlining tumorigenesis of thyroid follicular cells.

## RESULTS

### HuR expression in thyroid tissues

**Figure 1:**
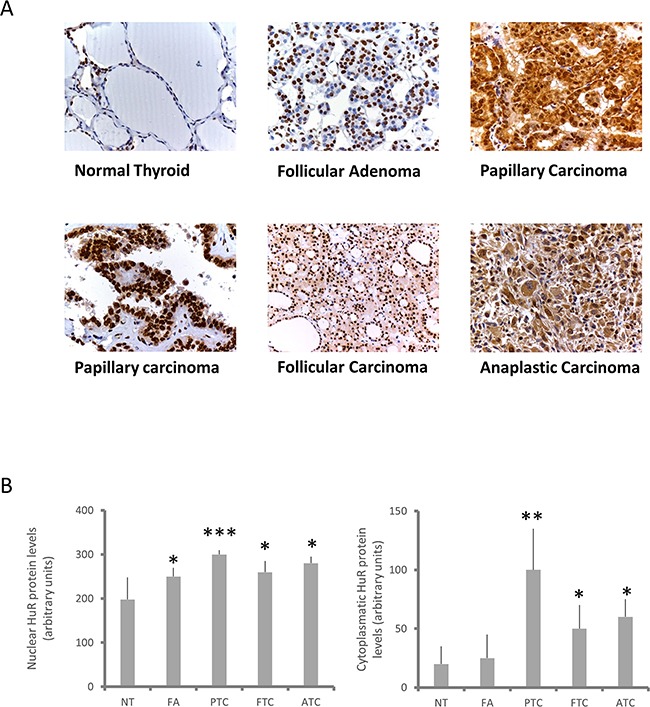
HuR expression in thyroid tissues **Panel A.** Immunohistochemical evaluation of HuR expression in normal thyroid tissues (NT), FAs, PTCs, FTCs and ATCs. Two PTC images are shown to highlight variation in HuR cytoplasmic levels. **Panel B.** Quantification of HuR expression in normal and neoplastic thyroid tissues. It was obtained by using the IHC score, calculated as described in Materials and Methods section. Results are shown as mean ± SD. * p < 0.05, ** p < 0.01, **** p < 0.0001 by ANOVA test.

In a first set of experiments we evaluated HuR protein expression and its sub-cellular localization in normal and tumor thyroid tissues. For this purpose we created a tissue microarray composed by 12 normal thyroid samples (NTs), 25 follicular adenomas (FAs), 23 follicular thyroid carcinomas (FTCs), 36 papillary thyroid carcinomas (PTCs) and 8 anaplastic thyroid carcinomas (ATCs), in order to perform an immunohistochemical HuR evaluation. Representative images of HuR staining are shown in panel A of Figure 1, indicating that all tumors displayed an increased HuR staining if compared to NT. However, important differences have been highlighted when nuclear and cytoplasmic staining were separately analyzed. Nuclear staining was significantly increased in all tumors, FAs included (Figure 1, Panel B, left side). In contrast, cytoplasmic staining shows dramatic differences between NT and FAs, on one side, and all malignancies on the other one (Figure 1, Panel B, right side). The higher cytoplasmic staining was observed in the PTC group, in which a 5-fold increment was observed in comparison to normal tissue. However, a wide variation of cytoplasmic staining was observed in PTCs (see also representative images in Panel A of Figure 1). Nevertheless, cytoplasmic HuR staining clearly discriminates between not only normal and tumor tissue, but even between malignant and benign neoplasia.

**Figure 2:**
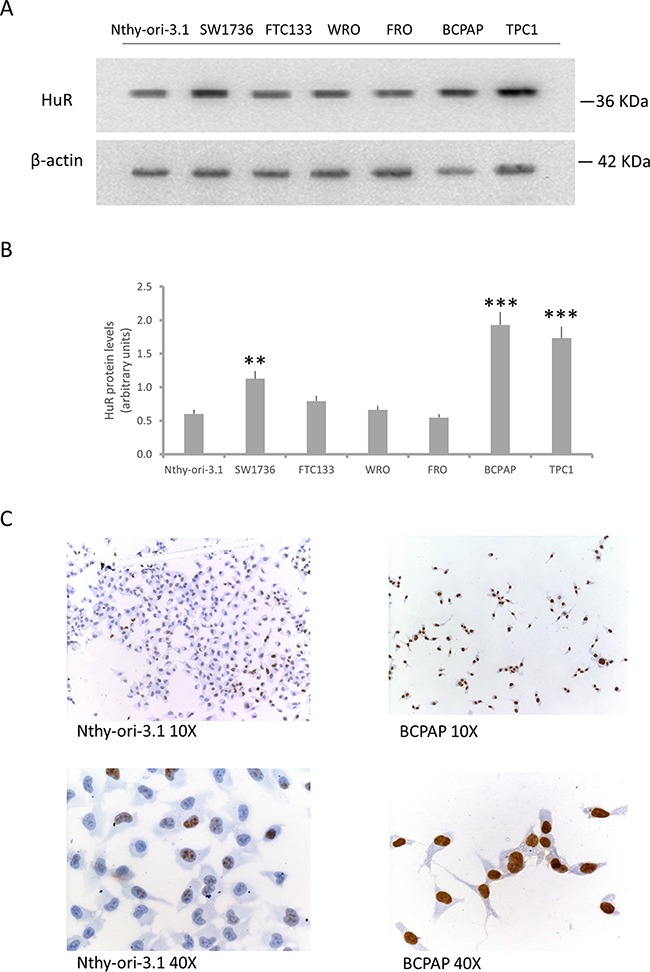
HuR expression in thyroid cell lines **Panel A.** Western blot analysis of HuR expression in a non-tumorigenic thyroid cell lines (Nthy-ori-3.1) and in six tumorigenic ones (SW1736, FTC133, WRO, FRO, BCPAP and TPC1). **Panel B.** Densitometric analysis of HuR protein levels in thyroid cell lines. **Panel C.** Immunocytochemical staining of Nthy-ori-3.1 and BCPAP cells. The brown signal indicates HuR positivity. Results are shown as mean ± SD. * p < 0.05, ** p < 0.01, **** p < 0.0001 by ANOVA test.

Based on immunohistochemical findings that suggest a relevant role of HuR in thyroid tumorigenesis, we used Western blot to evaluate expression of this protein in several thyroid cell lines. In particular, HuR protein levels were assessed in a non-tumorigenic thyroid cell line (Nthy-ory-3.1) and in six thyroid cancer cell lines (BCPAP, TPC1, FTC133, WRO, FRO and SW1736). As observed in thyroid tumor tissue, the Western blot analysis displayed a significant HuR over-expression in PTC cell lines and in SW1736 cells (Figure 2, Panel A-B). Thus, for further investigation we decided to use Nthy-ori-3.1, as model of non-tumorigenic thyroid cells and BCPAP, as model of tumorigenic ones. The HuR subcellular localization was assessed in Nthy-ori-3.1 and BCPAP cell lines by immunocytochemistry. As shown in Figure 2 (Panel C) HuR staining positivity was higher in BCPAP than in Nthy-ori-3.1. A higher HuR staining positivity was observed in BCPAP compared to Nthy-ory-3.1. Both cell lines show a HuR nuclear staining, which appears more prominent in the tumor cell line.

### HuR silencing effects

**Figure 3:**
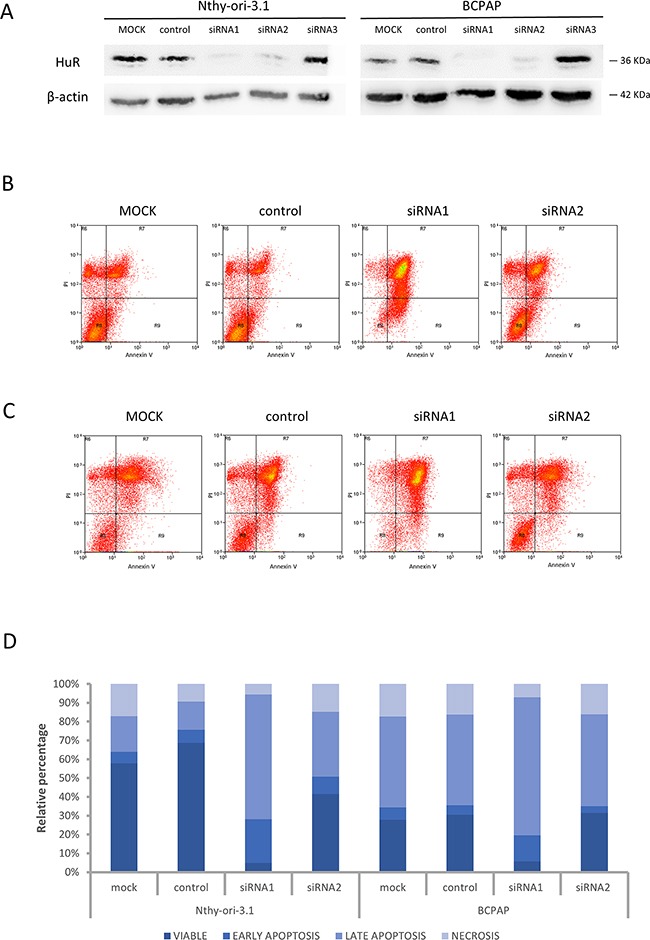
HuR silencing effects on cell viability **Panel A.** Nthy-ori-3.1 and BCPAP cells were transfected with non-targeting siRNA (NC, negative control) or three different siRNA sequence specific to HuR (5 nM) and collected after 72 h treatment. HuR protein levels were analyzed by Western Blot analysis, as described in Materials and Methods section. **Panel B-C.** Nthy-ori-3.1 (B) and BCPAP (C) were transfected to either siRNA1 or siRNA2 or negative control for 72 hours and cell death phenomena was analyzed by Annexin V and PI staining. **Panel D.** Histogram relative to Annexin V/PI analysis.

In order to evaluate biological HuR-related effects in thyroid cells, we performed an RNA interference assay. After HuR silencing, no protein levels were detectable by immunoblot analysis both in Nthy-ory-3.1 and in BCPAP (Figure 3, Panel A). Since it is described that HuR silencing could increase apoptotic phenomena [14], we analyzed cell viability and apoptosis levels after HuR silencing, by Annexin V/PI assay. In both cell lines, HuR silencing with siRNA1 reduced cell viability, about 93% and 80% in Nthy-ory-3.1 and BCPAP, respectively, and increased the percentage of apoptotic cells compared to negative control (Figure 3, Panel B - D). Hence, these data indicate that both in tumorigenic and non-tumorigenic thyroid cells, HuR plays a positive role in cell proliferation.

**Figure 4:**
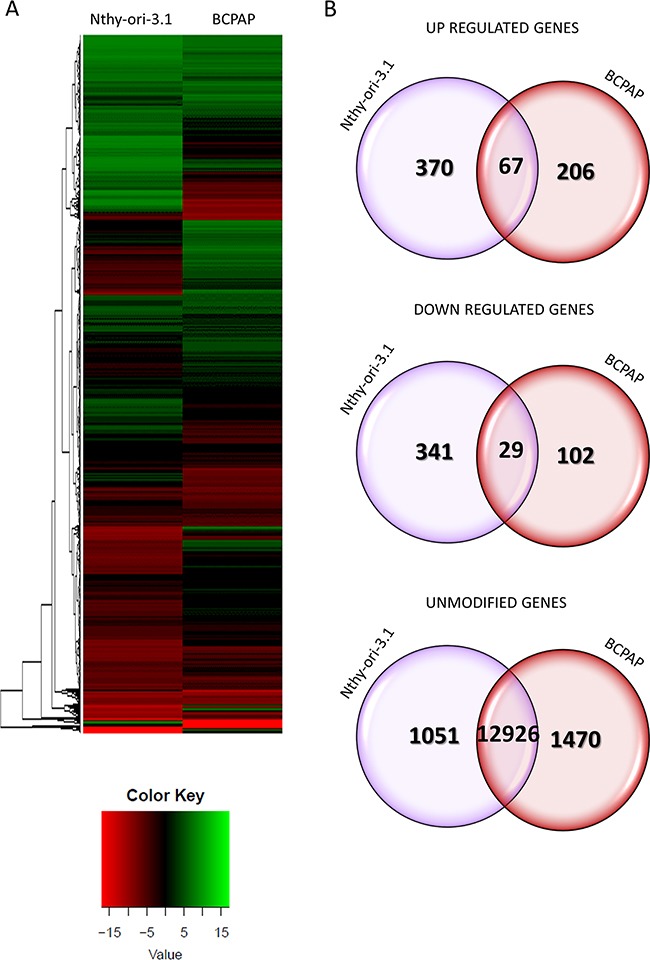
HuR silencing effects on gene expression **Panel A.** Heat maps showing the hierarchical clustering of RNA targets in Nthy-ori-3.1 and BCPAP cell lines. Cells were treated either with siRNA1 or negative control for 72 hours. Results are showed as siRNA1 compared to control transfected cells. **Panel B.** Venn diagrams represented the comparison of up-regulated, down-regulated and unmodified genes between Nthy-ori-3.1 and BCPAP cell lines after RNA-seq data analysis. The overlap of Nthy-ori-3.1 and BCPAP circles in Venn diagrams indicated shared modified genes between Nthy-ori-3.1 and BCPAP cell lines.

**Figure 5:**
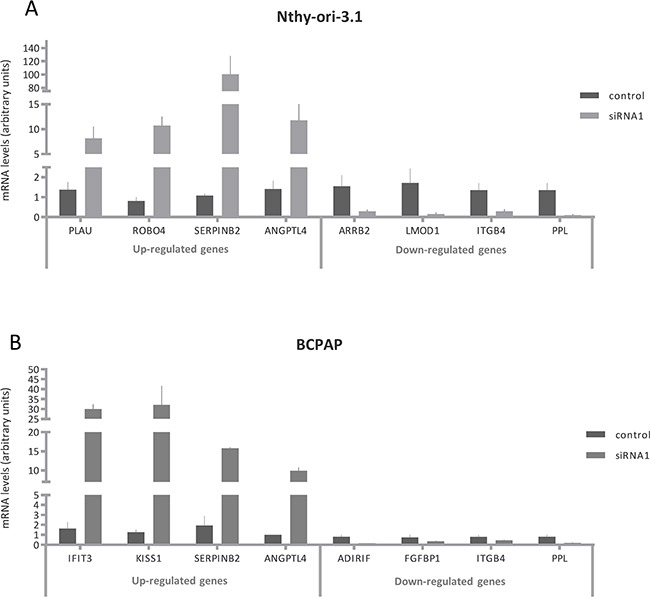
Validation of HuR silencing effects on gene expression Relative expression levels of *PLAU, ROBO4, SERPINB2, ANGPTL4, ARRB2, LMOD1, ITGB4, PPL, IFIT3, KISS1, ADIRIF* and *FGFBP1* after HuR silencing. RNA extraction and real time PCR are described in Materials and Method section. For each cell lines, the results were normalized against β actin and expressed in arbitrary unit, calculated as described in Materials and Methods section. Results are shown as mean ± SD of three different experiments. All silenced cell data were statistical significant compared to untreated cells.

As HuR is engaged in post-transcriptional gene regulation, we performed a high-throughput RNA sequencing analysis on Nthy-ori-3.1 and BCPAP transfected with siRNA1 or the negative control for 72 hours. To assess the transcription changes induced by HuR silencing, we compared siRNA1 to control-transfected cells. Data obtained highlighted that Nthy-ori-3.1 and BCPAP showed a quite different response to HuR silencing, as represented in the heat map in Figure 4 (Panel A). Expression of 14784 and 14800 genes was detected in Nthy-ori-3.1 and BCPAP cell lines, respectively. After filtering low quantity reads, results showed that 807 genes were differentially expressed after HuR silencing in Nthy-ory-3.1 (Supplementary Table S1), while, in BCPAP, the modified genes were 404 (Supplementary Table S2)(in both cell lines at a log2 fold change >2). In particular, 437 genes were up-regulated and 370 were down-regulated in Nthy-ori-3.1, while, in BCPAP, we identified 273 up-regulated genes and 131 down-regulated ones. As showed in the Venn diagrams, among the up-regulated genes, 67 were modified in both cell lines and, among the down-regulated genes, 29 were common between the two cell lines (Figure 4, Panel B). Target expression modifications induced by HuR silencing were assessed by quantitative RT-PCR both in sequenced samples and in replicate experiments. For this purpose, we chose 12 deregulated genes. The selection was based both on fold change values and on their biological function. Results are shown in Figure 5. For each selected gene, RNA-seq data were confirmed also in replicate experiments, indicating the high quality of our transcriptome analysis. In Tables 1 and 2 are listed the top 20 genes whose expression is modified by HuR silencing in both cell lines: as we can see, the majority of them belongs to the non-coding transcripts family, in particular miRNAs.

**Table 1:** Top 20 down-regulated and up-regulated genes by HuR silencing in Nthy-ori-3.1

TOP 20 Nthy-ori-3.1 down-regulated genes			TOP 20 Nthy-ori-3.1 up-regulated genes		
Gene	Control FPKM	siRNA FPKM	Gene	Control FPKM	siRNA FPKM
MIR103A2	26.53	0	OR5H14	0	0.70
MIR103B2	205.72	0	ACKR1	0	1.19
MIR1227	8.63	0	MIR5193	0	1.35
MIR1231	10.49	0	PTGIR	0	1.39
MIR1260B	12.48	0	BAALCOS	0	1.48
MIR1279	314.85	0	CASC8	0	1.48
MIR1304	11.15	0	CTSE	0	1.79
MIR1324	8.08	0	FDCSP	0	2.03
MIR15A	47.29	0	MIR5572	0	2.08
MIR181B2	12.28	0	MIR548I2	0	2.75
MIR191	13.06	0	CCL4	0	4.53
MIR192	5.77	0	MIR1178	0	4.86
MIR194-1	16.51	0	SNORA16B	0	5.04
MIR2278	14.21	0	MIR1224	0	5.21
MIR27B	10.30	0	MIR492	0	5.58
MIR302C	25.72	0	MIR221	0	5.72
MIR3176	43.99	0	SNORA79	0	6.68
MIR3190	22.54	0	MIR577	0	7.14
MIR3192	17.62	0	MIR1207	0	7.28
MIR320B2	2.41	0	SNORA70B	0	7.43

FPKM= Fragments Per Kilobase Of Exon Per Million Fragments Mapped

**Table 2:** Top 20 down-regulated and up-regulated genes by HuR silencing in BCPAP

TOP 20 BCPAP down-regulated genes			TOP 20 BCPAP up-regulated genes		
Gene	Control FPKM	siRNA FPKM	Gene	Control FPKM	siRNA FPKM
SNORD98	32.08	0	SNORA56	0	0.69
RIIAD1	0.52	0	MIR922	0	54.83
MT1JP	0.53	0	HIST1H4G	0	0.53
CLDN3	0.56	0	DDX26B	0	0.60
PPP1R14A	0.61	0	ZP4	0	0.61
FAM25A	1.13	0	KISS1	0	0.72
MIR5572	1.78	0	GNG8	0	1.24
SNORA70	2.46	0	IFNL2	0	1.24
MIR98	3.20	0	IFNB1	0	1.61
MIR941-2	5.45	0	MIR3689B	0	1.68
MIR765	6.58	0	IFNL1	0	2.12
MIR7110	7.27	0	SCARNA11	0	2.17
MIR569	7.29	0	MIR6129	0	3.32
MIR135A2	7.66	0	MIR5191	0	3.47
MIR10A	7.72	0	MIR5193	0	3.68
MIR567	7.80	0	MIR5189	0	3.71
MIR4441	7.85	0	P2RX6P	0	3.72
MIR6785	7.87	0	MIR454	0	4.07
SNORD23	8.07	0	MIR182	0	4.47
MIR345	8.48	0	MIR3940	0	4.58

FPKM = Fragments Per Kilobase Of Exon Per Million Fragments Mapped.

### HuR-bound RNA profiles

To understand whether the different effects observed after HuR silencing in Nthy-ori-3.1 and BCPAP cells could be related to a differential HuR binding pattern, we performed a HuR-RIP approach allowing the identification of HuR-bound RNA profiles in the two cell lines.

**Figure 6:**
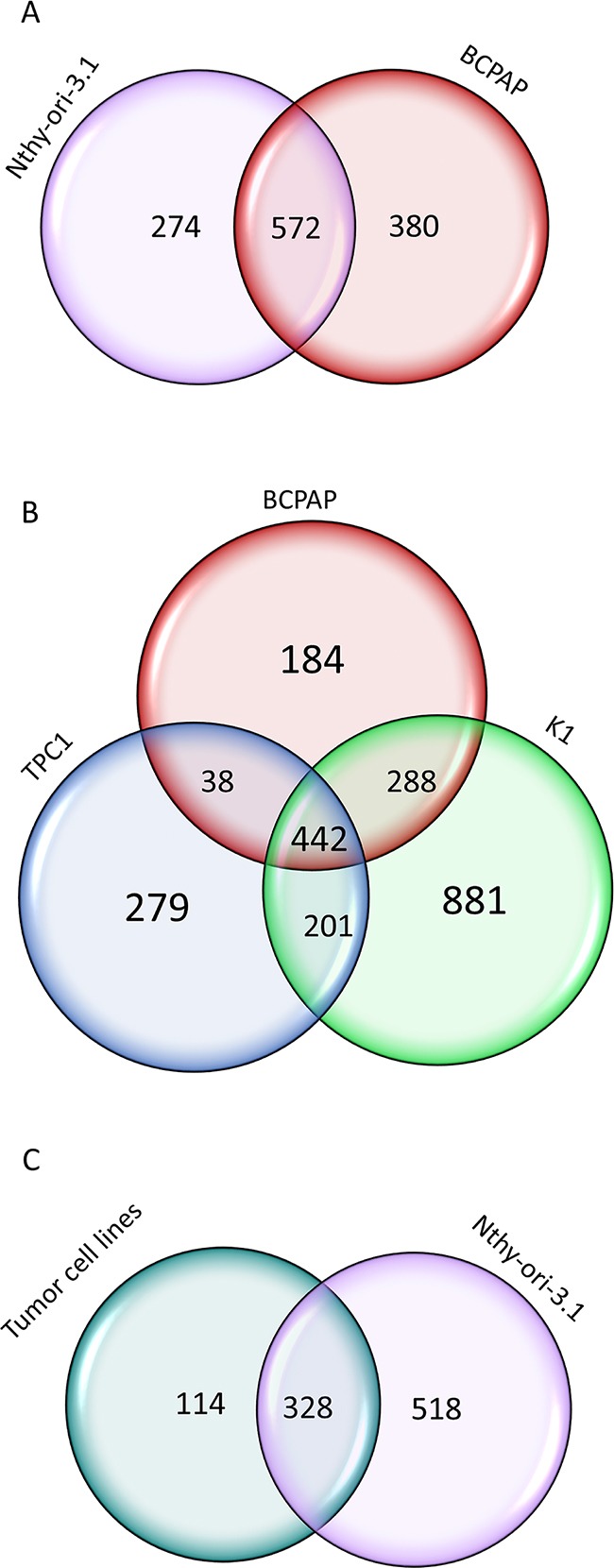
HuR-bound RNAs in thyroid cell lines **Panel A.** Venn diagrams representing the comparison of HuR RNA target identified in Nthy-ori-3.1 and BCPAP cells by RIP-seq analysis. **Panel B.** Venn diagrams representing the comparison of HuR RNA target identified in three PTC cell lines: BPCAP, K1 and TPC1 by RIP-seq analysis. **Panel C.** Venn diagrams representing the comparison of HuR RNA target identified in tumorigenic and non-tumorigenic cell lines.

By the high-throughput RIP sequencing analysis, we identified 846 and 952 transcripts significantly bound by HuR in Nthy-ori-3.1 and BCPAP, respectively. Among them, we identified several type of RNA: mRNA, miRNA and lncRNA. The HuR-bound RNA profiles identified by RIP-seq turned out to be quite different between non-tumorigenic and cancer cells. Comparing them, we identified 572 transcripts bound in both cell lines (Supplementary Table S3), while 274 RNAs were specifically bound only in Nthy-ori-3.1 cells (Supplementary Table S4) and 380 RNAs in BCPAP cells (Supplementary Table S5). In Figure 6 (Panel A), we provide a broad overview of RIP-seq results in terms of number of genes identified as significantly bound by HuR.

In order to find RNAs bound by HuR in a larger set of tumorigenic cells, we extended the RIP-seq analysis to other two PTC cell lines: K1 and TPC1. As shown in Figure 6 (Panel B), comparing the results obtained in the three tumorigenic cell lines, we identified 442 RNAs that were commonly bound by HuR in all of them. Comparing these 442 RNAs to those bound by HuR in Nthy-ori-3.1, we observed that 114 RNAs were specifically bound by HuR only in tumorigenic cell lines (Figure 6, Panel C)(Supplementary Table S6). Among these tumor cells specific HuR targets, we identified 13 genes that are already described as involved in tumorigenic processes (Table 3) and that may be related to the HuR tumor-promoting ability in thyroid cancer cells.

**Table 3:** Tumor specific HuR bound RNAs involved in tumorigenic processes

Gene	Function	Reference number
**TM4SF1**	Member of the transmembrane 4 superfamily, which mediate signal transduction events. This protein is highly expressed in different carcinomas.	[51]
**ZKSCAN3**	Transcription activator and promoter of cancer cell progression and/or migration in various tumors and myelomas.	[52]
**EREG**	Ligand of the EGF receptor/EGFR and ERBB4. Stimulates cell proliferation.	[53]
**MAP3K1**	Component of a protein kinase signal transduction cascade involved in tumorigenesis.	[54]
**PKN2**	PKC-related serine/threonine-protein kinase and Rho/Rac effector protein that participates in cell migration, cell adhesion and tumor cell invasion.	[55]
**AXL**	It transduces signals from the extracellular matrix into the cytoplasm. It is involved in proliferation, migration, resistance to apoptosis and survival.	[56]
**FGF5**	The protein encoded by this gene is a member of the fibroblast growth factor (FGF) family. This gene was identified as an oncogene, which confers transforming potential to mammalian cells.	[57]
**THBS1**	This protein is an adhesive glycoprotein that mediates cell-to-cell and cell-to-matrix interactions; it plays roles in platelet aggregation, angiogenesis, and tumorigenesis.	[58]
**EZR**	This protein plays a key role in cell surface structure adhesion, migration and organization, and it has been implicated in various human cancers.	[59]
**CPEB4**	CPEB4 regulates a range of biological processes involved in tumor and progression,. Moreover, CPEB4 is overexpressed in some human tumor types,	[60]
**ITGB1**	ITGB1 has been identified as a potential prognosis biomarker in triple negative breast cancer.	[61]
**SUPV3L1**	Major helicase player in mitochondrial RNA metabolism. May protect cells from apoptosis.	[62]
**RYK**	Co-receptor of Wnt proteins. RYK is essential for Wnt-5a-dependent invasiveness in human glioma.	[63]

In order to support the good outcome of our RIP approach, we matched our results with the BioGRID public database (http://thebiogrid.org/, accessed 07/13/2015), in which published genetic and protein interaction data are archived. Comparing our RIP-seq results with BioGRID records, we observed a matching of 51%, 46%, 42% and 45% of HuR targets in Nthy-ori-3.1, BCPAP, K1 and TPC1 respectively, with the database (data not shown). Among interesting HuR targets, already known in other tissues, our data include *i.e. HuR, eIF4E, BCL2, TP53, XIAP, MDM2, VHL, MYC*.

**Figure 7:**
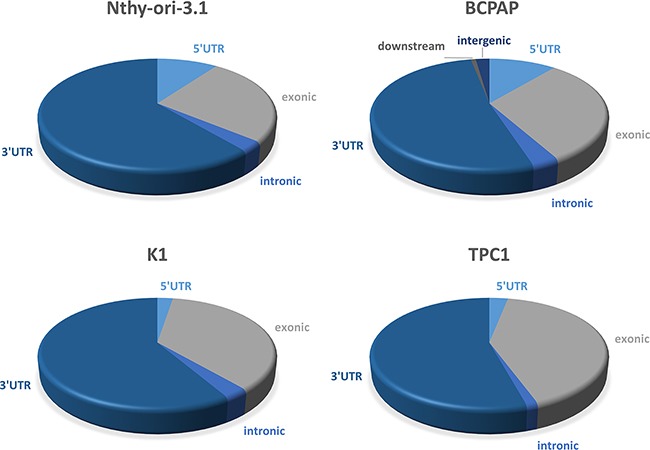
Localization of HuR-RNAs interaction region Graphical representation of Nthy-ori-3.1, BCPAP, K1 and TPC1 RIP-seq count distribution across different genomic features (UTR3, intronic, exonic, UTR5, intergenic and downstream). The count distribution was normalized for the number of each region present.

A large amount of data indicates that HuR often interacts with its cognate RNAs at 3′UTR level [5, 9, 15]. To evaluate if this behavior occurs also in Nthy-ori-3.1, BCPAP, K1 and TPC1 cell lines, we analyzed the count distribution across different genomic regions, setting a minimum coverage per feature of 500 reads, and then normalizing count distribution for the number of each region present. In this way, we observed that, also in our thyroid cells, the 3′UTRs were the regions with the highest coverage (Figure 7).

## DISCUSSION

HuR is a RNA-binding protein that plays a major role in regulation of gene expression [10] and that can contribute to tumorigenesis [7]. The HuR function is controlled at multiple levels. For example, HuR transcription, is positively regulated by the nuclear factor (NF)-κB and by Smad, while the abundance of HuR mRNA and protein are conditioned upon multiple regulatory mechanisms [10]. HuR is able to positively regulate its own mRNA modulating cytoplasmic export, stabilization, and translation. Furthermore, HuR protein is subjected to several post-translational modification, i.e. phosphorylation, methylation and ubiquitination, which affects HuR cytoplasmic levels, stability and interactions with, its targets [8, 10].

**Figure 8:**
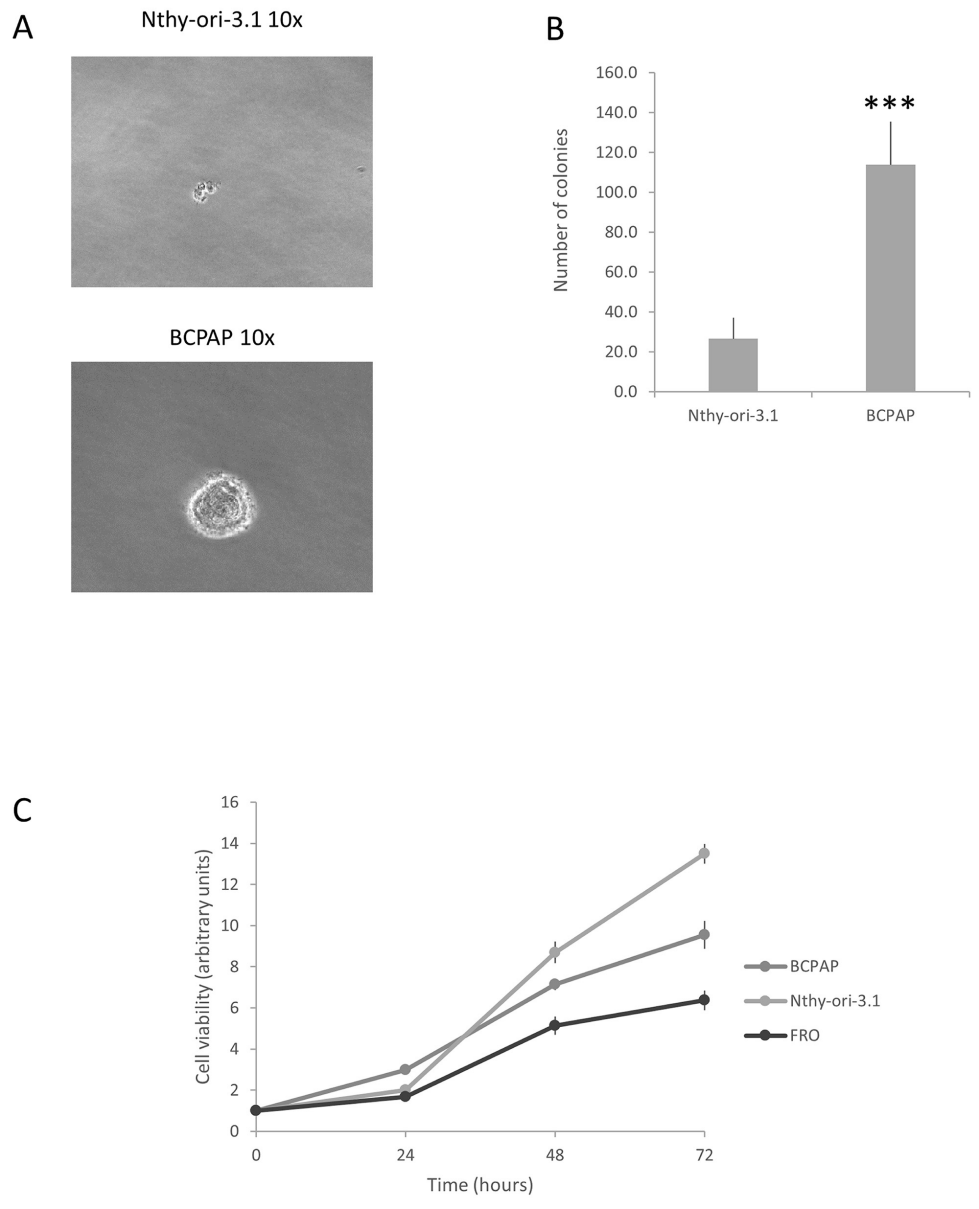
Nthy-ori-3.1 and BCPAP cell lines growth *in vitro* **Panel A.** Colony formation assay of Nthy-ori-3.1, BCPAP and K1. **Panel B.** Histogram representing the number of colonies per cell line. Results are shown as mean ± SD of three different experiments. *** p < 0.0001 by Student t test. **Panel C.** Cell viability of Nthy-ori-3.1, BCPAP and FRO cells was determined by MTT assay after 0, 24, 48 and 72 h and expressed as fold change respect t0. All samples were run in quadruplicate.

Several data indicate that HuR expression and localization are modified in cancer cells. In particular, a very frequent observation is the increase of HuR cytoplasmic localization [4, 16]. The relevance of HuR in tumorigenesis is highlighted by the evidence that this protein is currently explored as a target for anticancer treatment [17, 18]. Nothing is known, so far, about HuR expression and function in thyroid follicular cells. Therefore, investigation on this protein could provide innovative information on molecular mechanisms contributing to thyroid cancer. Coherently with data obtained in different neoplasia [19–22], we show that HuR is over-expressed in thyroid cancer. Moreover, the amount of cytoplasmic HuR appears to be significantly higher in thyroid malignancies (PTC, FTC and ATC) compared to NT or FA (Figure 1). When thyroid cancer cell lines were investigated, HuR over-expression was confirmed (Figure 2). Consistently with data obtained in tissues, both in Nthy-ori-3.1 and BCPAP cells, HuR localization was mostly nuclear, with a major expression in BCPAP. Nthy-ori-3.1 are non-tumorigenic cells, derived from normal thyroid follicular cells immortalized by the SV40 large T antigen [23], while BCPAP cells are derived from PTC bearing the BRAF V600E and TP53 D259Y mutations [24]. Although only BCPAP cells present features of invasiveness (i.e. growth in soft agar assay), Nthy-ori-3.1 and BCPAP grow *in vitro* with a similar high rate (Figure 8); therefore, both cell lines must be considered a model of thyroid hyper-proliferating cells. In this light, it is not surprising that HuR silencing reduced cell growth parameters in both cell lines. These results are coherent with available data on other cellular models; in fact, HuR silencing reduces cell growth of a wide range of cultured cells [25–28].

Considering HuR overexpression in thyroid cancer tissues as well as the results obtained by HuR silencing, indicating a HuR-dependent mechanism of thyroid cancer cell survival, this RBP might be consider an innovative therapeutic target for thyroid cancer.

How HuR silencing induces similar biological effects in Nthy-ori-3.1 and BCPAP cells, though the majority of molecular modifications are specific for the single cell line? Two, not mutually exclusive, possibilities can be put forward. The first is that effects of HuR silencing are due to genes modified in both cell lines. Indeed, *BIRC3* is up-regulated in both Nthy-ori-3.1 and BCPAP cells and is known to be involved in signaling of cell proliferation [29]. The second possibility is that HuR silencing-derived biological effects are due to expression modification of distinct genes between the two cell lines. For example, in BCPAP HuR silencing up-regulates *IFIT3*, which is an inhibitor of cell proliferation [30]. Conversely, in Nthy-ori-3.1, HuR silencing induces overexpression of *FOSL1*, which is involved in control of cell proliferation [31].

A major conclusion of our research is that in two cell lines originating from the same cell type (thyroid follicular cells) and both having a high proliferation rate, HuR shows significantly different spectra of both functional and interaction targets. Here, functional targets are defined as those whose expression is modified by HuR silencing, while interaction targets are those delineated by the RIP-seq assay. Our experiments indicate that among functional targets, only 96 out of 1115 are shared by Nthy-ori-3.1 and BCPAP cells, while among interaction targets 572 out of 1226 are commonly bound in the two cell lines. The notion that HuR is able to recognize distinct RNA spectra, by using RIP-chip experiments, has been described also during T cells activation. It has been shown, in fact, that, when Jurkat cells are stimulated by the phorbol ester PMA, only 405 among 1219 probes remain constant after 4 and 12 hours treatment [32]. Altogether, it could be envisaged that variation of HuR bound RNA species among cells with distinct biological properties is a common phenomenon. Our data represent a proof of concept that this variation exists between cells of the same origin but with different tumorigenic potential. Indeed, by comparison of RIP-seq data obtained in all cell lines, we observed a set of 114 HuR-bound RNAs common for tumorigenic cell lines and not for non-tumorigenic cells. An open question is to understand which phenotypic characteristics are responsible for this HuR behavior in non-tumorigenic and tumorigenic cells. Our immunohistochemical data indicate that the differences observed between the two cell lines are not due to a different protein localization (Figure 2, Panel C). Thus, a possible explanation of HuR plasticity, in terms of both functional and interaction targets, could be related to a difference its post-translational modifications in the two cell lines. HuR, indeed, is subjected to several post-translational modifications that influence its functions and its subcellular localization. For example, HuR phosphorylation by Chk2, Cdk1 or p38 and HuR methylation by CARM1 regulate both its cytoplasmic levels as well as the protein-mRNA interactions [9].

Another possible event that could influence HuR-mRNA binding is the presence of different RNA post-transcriptional modifications on its targets. Nowadays more than 100 kinds of RNA modifications have been identified and this discovery highlights the hypotesis that RNA modifications may act as epigenetic markers [33]. The most abundant mRNA modification is N^6^-methyl-adenosine (m^6^A). This RNA modification is present in more than three sites per mRNA molecule on average and is enriched at the 3′ UTR [34]. Wang and colleagues have demonstrated that HuR regulates the stability of many mRNAs in embryonic stem cells in a m^6^A-dependent manner, proving in particular, that the m^6^A methylation loss enhances HuR mRNA binding [35].

**Figure 9:**
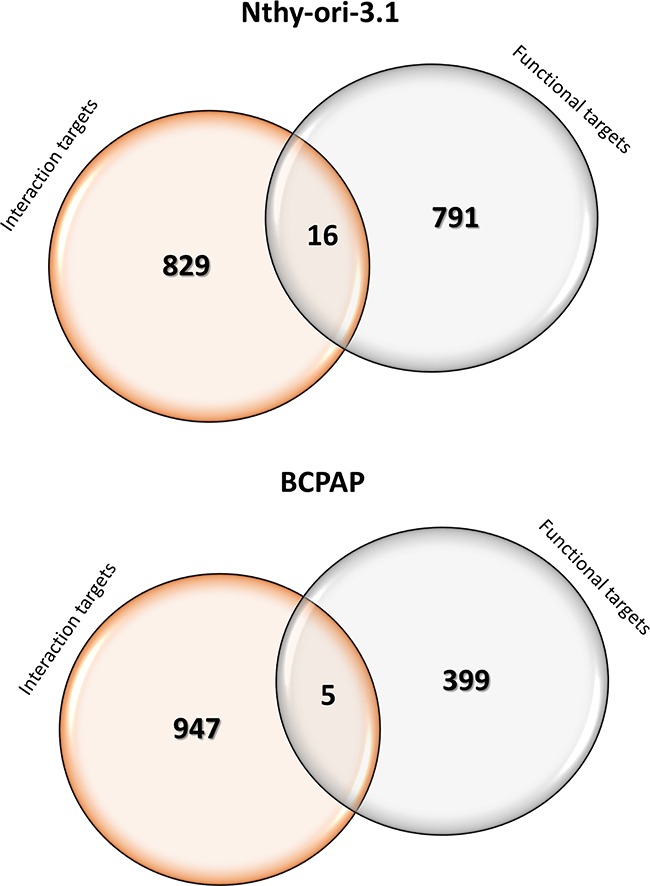
Functional and interaction HuR target Venn diagrams representing the comparison between RNA-Seq (Functional) and RIP-Seq (Interaction) HuR-target genes identified in Nthy-ori-3.1 and BCPAP cells. In the circle overlapping area genes that were both Functional and Interaction HuR targets.

Our data indicate that in both cell lines, only a small fraction of functional targets is also interaction targets. In fact, in Nthy-ori-3.1 only 16 functional targets are bounded by HuR, while in BCPAP cells only 5 RNAs modified by HuR silencing are interaction targets (Figure 9). The notion of a minimal overlap between HuR functional and interaction targets has been also recently observed using a HuR conditional knockout model in CD4+ T cell activation [36]. In that study, in fact, 2068 “functional” and 271 “interaction” HuR targets have been identified, but only 30 were shared by the two groups. Even if only few data are today available, the minimal overlap between “functional” and “interaction” targets seems to be a common phenomenon of RBPs. In fact, for the yeast Puf3p protein, it has been shown that the expression of only 82 out of 1132 interaction targets was modified when transcriptomics of parental and Puf3p-deleted strains were compared [37]. We have to consider that these technological approaches are still new and major comprehension of their potential could improve the interpretation of data obtained so far. However, the accuracy of our RNA-seq analysis is demonstrated by the consistency for all tested genes, whose expression has been evaluated by qPCR. Moreover, the RIP-seq analysis quality is proved by its overlap with the BIOGRID database: in fact, 51%, 46%, 42% and 45% of RNAs found as HuR targets in Nthy-ori-3.1, BCPAP, K1 and TPC1, respectively, are also listed in this database. Furthermore, the HuR RNA targets identified by RIP-seq and also listed in the BIOGRID database include several known HuR targets, i.e. *HuR, eIF4E, BCL2, TP53, XIAP, MDM2, VHL, MYC*.

In order to explain the slight overlap between functional and interaction HuR targets, the relevance of miRNAs as HuR co-actors and/or antagonists must be considered [38]. In fact, our RNA-seq analysis showed that miRNAs are the most misregulated transcripts after HuR silencing. Therefore, we could hypothesize that HuR knockdown caused a switch to an alternative gene expression program, in order to overcome the RBP absence. This may occur by increasing the expression of miRNAs operating as HuR co-actors, and by down-regulating HuR antagonist miRNAs. Such a possibility is positively supported by our transcriptomic data. In fact, expression of the LET-7, a HuR miRNA co-actor [39, 40], was up-regulated in both cell lines upon HuR silencing and the opposite phenomenon occurs for miR34a expression, a HuR antagonist [39, 41]. miRNAs whose expression is modified by HuR silencing might play a role in thyroid tumorigenesis. For example, miR181b is downregulated upon HuR silencing and it is known that its downregulation is associated to promote apoptosis in PTCs [42]. Thus, further investigations on the relationship between HuR and noncoding RNAs (both lnRNAs and miRNAs) could reveal important aspects of thyroid tumorigenesis.

In conclusion, our findings indicate that RBP targets may be different between cells with the same origin but with different aggressiveness. Therefore, investigation on this phenomenon could provide relevant information to understand the molecular derangements occurring in cancer cells.

## MATERIALS AND METHODS

### Human tissues and cell lines

A series of 12 normal thyroid glands (NTs), 25 follicular adenomas (FAs), 23 follicular thyroid carcinomas (FTCs), 36 papillary thyroid carcinomas (PTCs) and 8 anaplastic thyroid carcinomas (ATCs) were selected from the files of the Institution of Anatomic Pathology of the University of Udine and the most representative blocks of each lesion were retrieved from the archive. All samples were diagnosed by referral pathologists of institutions and then reviewed by a single experienced pathologist, thus including only patients with a confirmed diagnosis. The study has been approved by the “Sapienza” University Ethical Committee.

In this study, we used 8 different thyroid cell lines: Nthy-ori-3.1, derived from normal thyroid follicular epithelial cells and immortalized by the SV40 large T gene; BCPAP, K1 and TPC1, derived from papillary thyroid carcinoma; FTC133 and WRO, from follicular thyroid carcinoma; FRO and SW1736, from anaplastic thyroid cancer. All cell lines have been validated by short tandem repeat and tested for being mycoplasma-free.

FRO, TPC1 and FTC133 cells were grown in DMEM medium (EuroClone, Milan, Italy) while the others were grown in RPMI 1640 medium (EuroClone). Both media were supplemented with 10% fetal bovine serum (Gibco Invitrogen, Milan, Italy), 2 mM L-glutamine (EuroClone) and 50 mg/ml gentamicin (Gibco Invitrogen). Cells were grown in a humidified incubator (5% CO_2_ in air at 37°C) (Eppendorf AG, Hamburg, Germany).

### Immunohistochemistry

A tissue microarray was created with representative tumor-bearing areas of the selected thyroid tissues. Briefly, 5 μM formalin-fixed paraffin tissue sections mounted on SuperFrost Plus slides (Menzel-Gläser, Braunschweig, Germany) were placed in the PT Link pre-treatment module (DAKO A/S, Glostrup, Denmark), which performs automatically the entire pre-treatment process of de-paraffinization, rehydration, and epitope retrieval using the Low pH Target Retrieval Solution (0.001 M citrate buffer pH 6.0) at 98°C for 40 min from DAKO. Endogenous peroxidase activity was blocked by incubation in the Peroxidase Block solution (DAKO) for 5 minutes.

As for cell cultures, Nthy-ori-3.1 and BCPAP cells were fixed with 5% glacial acetic acid in ethanol for 10 min at 4°C, and rinsed with 0.1% saponin (Sigma Chemical Co, St Louis, Missouri, USA) in phosphate-buffered saline (PBS). This PBS–saponin solution was used for all subsequent washing steps.

The tissue microarray and the cell cultures slides were incubated with primary rabbit polyclonal antiserum to HuR (Millipore) diluted 1:50 for 60 minutes at room temperature. After washing, slides were incubated with the DAKO EnVision FLEX System (DAKO) according to manufacturer's guidelines. For reaction visualization, 3-3 diaminobenzidine tetrahydrochloride was used as chromogen. The sections were counterstained with Mayer's hematoxylin. Using light microscopy, the entire section was scanned at high-power magnification (400x) and nuclear immunostaining was evaluated by using the H-score method. The H-score is a well-established semi-quantitative evaluation of immunohistochemical data [43]. It is calculated by multiplying the percentage of immunoreactive cells and the intensity of the staining, which is graded as low (score 1), moderate (score 2) or strong (score 3). Thus, the range of possible scores was from 0 to 300. Each cellular location (cytoplasm and nucleus) was separately scored. H-score was determined by two experienced pathologists: for each sample the final H-score corresponds to the mean value of these two independent evaluations.

### Protein extraction and western blot

Total protein extraction was performed as previously described [44]. Briefly, Nthy-ori-3.1, SW1736, FRO, WRO, FTC133, TPC1 and BCPAP cells were harvested by scraping and lysed with total lysis buffer (Tris HCl 50 mM pH8, NaCl 120 mM, EDTA 5 mM, Triton 1%, NP40 1%, protease inhibitors).

For nuclear and cytoplasmic protein extraction, Nthy-ori-3.1 and BCPAP cells were lysed by using two buffer with different ionic force: B1 (HEPES pH 7.9 10 mM, KCl 10 mM, MgCl_2_ 0.1 mM, EDTA pH 8 0.1 mM) for cytoplasmic proteins extraction and B2 (HEPES pH 7.9 20 mM, NaCl 420 mM, MgCl_2_ 1.5 mM, EDTA pH 8 0.1 mM, glicerolo 5%) for nuclear proteins extraction.

For Western Blot analysis, proteins were electrophoresed on 12% SDS-PAGE and then transferred to nitrocellulose membranes (GE Healthcare, Little Chalfont, UK), saturated with 5% non-fat dry milk in PBS/0.1% Tween 20. The membranes were then incubated overnight with rabbit polyclonal anti-HuR antibody 1:500 (Millipore) or rabbit anti-β-actin antibody 1:1000 (Abcam, Cambridge, UK). The day after, membranes were incubated for 2 h with anti-rabbit immunoglobulin coupled to peroxidase 1:4000 (Sigma-Aldrich). Blots were developed using UVITEC Alliance LD (UVITec Limited, Cambridge, UK) with the SuperSignal Technology (Thermo Scientific Inc).

### HuR silencing

For transient silencing of endogenous HuR, TriFECTa RNAi Kit (Integrated DNA Technologies Inc, Coralville, IA, USA) was used following manufacturer's instructions and a previously published procedure [45]. Briefly, a ‘universal’ negative control duplex, that targets a site absent in human genome, was used. Three different siRNA oligonucleotides (siRNA1, siRNA2 and siRNA3) were transfected at a concentration of 5 nM using DharmaFECT 1 Transfection reagent (Thermo Scientific Inc, Waltham, MA, USA), according to manufacturer's instructions. The concentration of 5 nM was set performing dose-response studies (data not shown). The day before transfection, cells were plated in antibiotics-free medium. Cells were harvested 72 h after transfection and gene-silencing efficiency was evaluated by protein levels analysis.

### Annexin V assay

Nthy-ori-3.1 and BCPAP cells, transfected with siRNA oligonucleotides or control, were washed with cold PBS, transferred to a polystyrene round-bottomed flow tube (Falcon, Becton Dickinson, Franklin Lakes, NY) and resuspended in 195 μL of 1x binding buffer (BB 10 mM; HEPES/NaOH, pH 7.4, 140 mM NaCl, and 2.5 mM CaCl_2_). 5 μL of Fluorescein-conjugated Annexin V (Annexin V-FITC; Bender Med Systems, Vienna, Austria) was added and samples were incubated for 10 minutes at room temperature. After washing, cells were resuspended in 190 μL of BB in which 10 μL of propidium iodide stock solution (final concentration 1 μg/mL) was added. Flow cytometry analysis was performed on CyAN, Dako Cytomation using the Summit software (Flow cytometer, Beckman Coulter). Forward scatter (FSC) and side scatter (SSC) were acquired in linear mode. FITC and PI fluorescent signals derived from 488 nm excitation were detected in logarithmic mode at FL1/PMT3 and FL2/PMT4, with FITC 530/30 nm filters, and PI 585/42 nm filters, respectively, and a FL1/2 560 nm short pass dichroic filter. Signals for forward and side scatter and fluorescence were collected for 10^4^ cells using the forward light scatter parameter as the master signal. Data are expressed as mean fluorescence intensity (FI) values.

### Library preparation and sequencing

Total RNA from Nthy-ori-3.1 and BCPAP cell lines was extracted with RNeasy mini kit according to manufacturer's instructions (Qiagen, Hilden, Germany). TruSeq Stranded Total RNA with Ribo-Zero Human/Mouse/Rat kit (Illumina, San Diego, CA) has been used for library preparation following the manufacturer's instructions, starting with 200 ng of good quality RNA (R.I.N. >7) as input. Both RNA samples and final libraries were quantified by using the Qubit 2.0 Fluorometer (Invitrogen, Carlsbad, CA) and quality tested by Agilent 2100 Bioanalyzer RNA Nano assay (Agilent technologies, Santa Clara, CA). Libraries were then processed with Illumina cBot for cluster generation on the flowcell, following the manufacturer's instructions and sequenced on 50 bp single-end mode at the on HiSeq2500 (Illumina, San Diego, CA). The CASAVA 1.8.2 version of the Illumina pipeline was used to processed raw data for both format conversion and de-multiplexing.

### RNA-Seq bioinformatics analysis

Raw sequence files were subjected to quality control analysis using FastQC (http://www.bioinformatics.babraham.ac.uk/projects/fastqc/). In order to avoid low quality data, adapters were removed by Cutadapt [46] and lower quality bases were trimmed by ERNE [47]. For the analysis of differentially expressed genes, the quality-checked reads were processed using the TopHat version 2.0.0 package (Bowtie 2 version 2.2.0) as FASTQ files [48–50]. The reads were mapped to the human reference genome GRCh37/hg19. Reads abundance was evaluated and normalized by using Cufflinks [49] for each gene and FPKM (Fragments Per Kilobase Of Exon Per Million Fragments Mapped) values were obtained.

Cuffdiff from the Cufflinks 2.2.0 package was used to calculate the differential expression levels and to evaluate the statistical significance of detected alterations between control and silenced samples, in both cell lines. For further analysis, we selected effective data using three criteria: a) FPKM values > 0.5; b) log2 fold-change >2 and c) q-value (false discovery rate (FDR)) <0.005.

### Gene expression assays

150 ng of total RNA of Nthy-ori-3.1 and BCPAP cells, transfected with siRNA1 or control, were reverse transcribed to cDNA using random exaprimers and SuperScript III reverse transcriptase (Life Technologies, Carlsbad, CA, USA). Real-time PCR was performed using Platinum Sybr Green QPCR supermix (Life Technologies) with the ABI Prism 7300 Sequence Detection Systems (Applied Biosystems). The ΔΔCT method, by means of the SDS software (Applied Biosystems), was used to calculate mRNA levels. Oligonucleotide primers were purchased from Sigma-Aldrich and their sequences are available upon request.

### RNA-binding protein immunoprecipitation

The RNA-binding protein immunoprecipitation (RIP) was performed using EZ-Magna RIP kit (Millipore) according to manufacturer's instructions.

In order to perform RIP assay, Nthy-ory-3.1, BCPAP, K1 and TPC1 cells were scraped in PBS containing protease inhibitors and then resuspended in RIP lysis buffer (Millipore) containing protease and RNase inhibitors.

Magnetic Beads Protein A/G were incubated overnight with 5 μg of rabbit polyclonal anti-HuR RIPAb+ antibody (Millipore) or normal Rabbit IgG (Millipore) as negative control. The day after, samples were added to antibody-bead complexes and incubated overnight. 10% of samples was stored as total input.

After washings, immune-complexes and input were eluted and treated with proteinase K and heated at 55°C for 30 minutes to digest the protein. RNA was purified with phenol/chloroform extraction followed by ethanol precipitation. RNA obtained from RIP was sequenced and analyzed as described above.

### Cell viability

Methylthiazolyldiphenyl-tetrazolium bromide (MTT) was applied to test cell viability. Nthy-ori-3.1, BCPAP and FRO cells (3000 cells/well) were plated onto 96-well plates in 200 μl medium/well and were allowed to attach to the plate for 24 h (t0). Plates were then incubated for 24, 48 and 72 h. The cell medium was then replaced with 200 μl fresh medium/well containing 0.5 mg/ml MTT (Sigma-Aldrich) and cultivated for another 4 h darkened in the cells incubator. The supernatant was removed, 100 μl/well of DMSO (Sigma-Aldrich) were added and the absorbance at 570 nm was measured. All experiments were run in quadruplicate and cell viability was expressed as a fold change respect t0.

### Soft agar assay

Nthy-ori-3.1 and BCPAP cell lines clonogenic activity was evaluated by soft agar assay. Briefly, 10000 cells per plate were suspended in 4 ml of complete medium containing 0.25% agarose and then seeded to the top of a 1% agarose complete medium layer in 6 cm plates. The colonies were counted by the inverted microscope Leica DMI-600B (Leica Microsystems Ltd., Heerbrugg, Switzerland). Data are representative of three independent experiments.

### Statistical analysis

mRNA and protein levels, cell viability and apoptosis levels were expressed as means ± SD, and significances were analyzed with either the Student's t-test or one-way ANOVA, both performed with GraphPAD Software for Science (San Diego, CA, USA).

## SUPPLEMENTARY TABLES














