# Supplementary material for: Identification of tumorigenesis-related mRNAs associated with RNA-binding protein HuR in thyroid cancer cells
Source: Oncotarget. 2016 Aug 12;7(39):63388–407. doi: 10.18632/oncotarget.11255 (PMC5325372; doi:10.18632/oncotarget.11255)
Supplement: Supplementary file 1 [file oncotarget-07-63388-s001.pdf]

Identification of tumorigenesis-related mRNAs associated with RNA-binding protein HuR in thyroid cancer cells

SUPPLEMENTARY TABLES

Supplementary Table S1: Nthy-ori-3.1 genes modified by HuR silencing.

See Supplementary File 1

Supplementary Table S2: BCPAP genes modified by HuR silencing.

See Supplementary File 2

Supplementary Table S3: Nthy-ori-3.1 and BCPAP common HuR interaction target RNA.

See Supplementary File 3

Supplementary Table S5: BCPAP specific HuR interaction target RNA.

See Supplementary File 4

Supplementary Table S4: Nthy-ori-3.1 specific HuR interaction target RNA.

See Supplementary File 5

Supplementary Table S6: Tumor specific HuR interaction target RNA.

See Supplementary File 6
