# Supplementary material for: Identification of tumorigenesis-related mRNAs associated with RNA-binding protein HuR in thyroid cancer cells
Source: Oncotarget. 2016 Aug 12;7(39):63388–407. doi: 10.18632/oncotarget.11255 (PMC5325372; doi:10.18632/oncotarget.11255)
Supplement: Supplementary file 2 [file oncotarget-07-63388-s002.docx]

*Supplementary Table 1. Nthy-ori-3.1 genes modified by HuR silencing*

| gene | CN FPKM | siRNA1 FPKM | Fold Change (log2) |
| --- | --- | --- | --- |
| ABCA4 | 0.098703 | 0.675841 | 2.775518034 |
| ACADS | 5.03527 | 1.24038 | -2.021286971 |
| ACKR1 | 0 | 1.18703 | Infinite |
| ACOX2 | 0.0294955 | 0.613603 | 4.378738766 |
| ACSL5 | 2.28111 | 16.2586 | 2.833395112 |
| ACTN2 | 0.216302 | 1.44844 | 2.743381014 |
| ACVRL1 | 0.194655 | 1.72734 | 3.149560775 |
| ADAM12 | 0.554779 | 2.91312 | 2.392580048 |
| ADAM8 | 0.657825 | 4.38831 | 2.737889702 |
| ADAMTS14 | 0.900975 | 0.178901 | -2.332325623 |
| ADAMTS15 | 4.52817 | 0.0855483 | -5.726045126 |
| ADAMTSL4 | 14.5493 | 3.06826 | -2.245457098 |
| ADORA2A-AS1 | 0.22123 | 0.940161 | 2.087360799 |
| ADRB2 | 5.80426 | 28.4292 | 2.292189451 |
| ADTRP | 0.522587 | 3.14275 | 2.588284372 |
| AIM1L | 2.8417 | 0.356442 | -2.99501501 |
| AIM2 | 0.0109435 | 2.12674 | 7.602425639 |
| AK5 | 0.383666 | 2.64578 | 2.785770279 |
| ALPP | 15.3717 | 0.604738 | -4.667822679 |
| ALPPL2 | 0.782002 | 0.0157099 | -5.637426395 |
| AMIGO2 | 14.4144 | 89.1109 | 2.628091126 |
| AMOT | 8.78837 | 1.67505 | -2.39139145 |
| ANGPTL4 | 1.0179 | 8.13592 | 2.998709657 |
| ANKRD1 | 126.276 | 677.618 | 2.423891732 |
| ANKRD2 | 3.31267 | 0.260584 | -3.668174083 |
| ANO2 | 11.2179 | 1.21985 | -3.201026966 |
| ANPEP | 1.20455 | 5.47019 | 2.183096664 |
| ANTXR2 | 0.309451 | 6.18 | 4.319823949 |
| APOE | 1.27663 | 0.144692 | -3.141283393 |
| ARHGAP28 | 0.835169 | 0.166624 | -2.325471946 |
| ARHGAP33 | 1.13386 | 0.121726 | -3.219533261 |
| ARL14EPL | 0.181433 | 1.03927 | 2.518061722 |
| ARRB2 | 23.7098 | 5.909 | -2.004497589 |
| ARVCF | 3.5507 | 0.804831 | -2.141345691 |
| ASB2 | 0.0937678 | 0.693574 | 2.886885328 |
| ASIC3 | 1.54849 | 0.362652 | -2.094204356 |
| ATP6V1B1 | 2.54982 | 0.126121 | -4.337514987 |
| BAALC | 0.0903826 | 0.607317 | 2.748332789 |
| BAALCOS | 0 | 1.47597 | Infinite |
| BAIAP2L2 | 0.115177 | 0.513119 | 2.155440797 |
| BATF2 | 7.60846 | 33.5457 | 2.140451471 |
| BCAM | 19.9366 | 1.35348 | -3.880673918 |
| BCL2A1 | 0.0458629 | 1.29077 | 4.81476056 |
| BIRC3 | 5.40496 | 141.331 | 4.708650194 |
| BLID | 0.0467343 | 0.502907 | 3.427737944 |
| BLK | 0.0185916 | 0.628344 | 5.078831769 |
| BLOC1S5-TXNDC5 | 4.64796 | 0.00138625 | -11.71119448 |
| BMP4 | 4.0955 | 0.72287 | -2.502231471 |
| BMPER | 0.575778 | 4.82204 | 3.066059048 |
| BPIFA2 | 0.603065 | 0.140683 | -2.099865503 |
| BRICD5 | 3.45445 | 0.655533 | -2.397715717 |
| C10orf32-ASMT | 5.25439 | 0.00428957 | -10.25847454 |
| C10orf54 | 8.85184 | 1.98293 | -2.158343625 |
| C11orf91 | 0.126564 | 0.956579 | 2.918017018 |
| C15orf48 | 14.3186 | 304.122 | 4.408687839 |
| C1QL1 | 9.48229 | 1.96179 | -2.2730649 |
| C1QTNF1 | 1.91783 | 10.0141 | 2.384486019 |
| C1QTNF1-AS1 | 0.242253 | 1.03363 | 2.093133412 |
| C1R | 16.1801 | 75.9829 | 2.231454251 |
| C1S | 6.4106 | 101.231 | 3.981047952 |
| C3 | 30.0606 | 128.013 | 2.090344616 |
| C3AR1 | 0.213453 | 2.25317 | 3.399965824 |
| C6orf141 | 0.0515759 | 0.528914 | 3.358264167 |
| C6orf58 | 0.0773125 | 0.593268 | 2.939910373 |
| C8orf37-AS1 | 0.693094 | 0 | Infinite |
| C8orf44-SGK3,SGK3 | 0.256912 | 1.37251 | 2.417470477 |
| CACFD1 | 3.5335 | 0.801328 | -2.140633116 |
| CACNG4 | 0.804704 | 0.135416 | -2.571059994 |
| CADM4 | 34.5339 | 6.34251 | -2.444887477 |
| CASC8 | 0 | 1.47896 | Infinite |
| CASP1 | 3.24396 | 20.9297 | 2.689723697 |
| CASP4 | 12.8 | 61.444 | 2.263128328 |
| CASS4 | 0.361836 | 2.78204 | 2.942735305 |
| CCDC146 | 1.53721 | 0.215599 | -2.833891875 |
| CCDC153 | 1.69666 | 0.401151 | -2.080480188 |
| CCDC169-SOHLH2 | 0.714223 | 0.000695878 | -10.00332448 |
| CCDC19 | 0.138172 | 1.05 | 2.925852134 |
| CCDC88B | 1.4047 | 0.187671 | -2.90398441 |
| CCHCR1 | 0.530178 | 0.104839 | -2.338301308 |
| CCL17 | 0 | 18.9987 | Infinite |
| CCL2 | 2.43452 | 57.5103 | 4.562111106 |
| CCL20 | 0.816319 | 64.4856 | 6.303700188 |
| CCL26 | 1.79493 | 8.21247 | 2.193888613 |
| CCL27 | 0.992388 | 0.173362 | -2.517116588 |
| CCL3 | 0 | 94.901 | Infinite |
| CCL4 | 0 | 4.53494 | Infinite |
| CCL5 | 9.94911 | 384.456 | 5.27210721 |
| CCR7 | 0.349176 | 7.90795 | 4.501277443 |
| CD163L1 | 0.0525149 | 0.711282 | 3.759622934 |
| CD200 | 0.314038 | 1.61253 | 2.360314954 |
| CD22 | 0.595836 | 2.43308 | 2.029796559 |
| CD274 | 4.38979 | 27.7373 | 2.659603529 |
| CD34 | 0.284534 | 1.28037 | 2.169887818 |
| CD38 | 0.0268056 | 0.590118 | 4.460397132 |
| CD44 | 12.2539 | 134.378 | 3.454984075 |
| CD68 | 5.971 | 56.0974 | 3.231889433 |
| CD82 | 6.28994 | 29.6122 | 2.235073517 |
| CDC42BPG | 2.29614 | 0.353545 | -2.699244847 |
| CDCP1 | 6.61953 | 32.535 | 2.297191862 |
| CDH1 | 3.57517 | 0.328189 | -3.445413054 |
| CDH13 | 9.95053 | 41.073 | 2.045345051 |
| CEACAM1 | 0.271767 | 1.60365 | 2.560917116 |
| CELSR3-AS1 | 3.76876 | 0.593231 | -2.66742403 |
| CFH | 1.75061 | 7.66346 | 2.13013819 |
| CFI | 0.650802 | 0.121814 | -2.417538734 |
| CGB7 | 0.988478 | 0.20092 | -2.298587677 |
| CGN | 2.43781 | 0.585582 | -2.057642575 |
| CH25H | 0.105739 | 6.1627 | 5.864983069 |
| CHST11 | 0.847459 | 5.68933 | 2.747043287 |
| CHST2 | 0.593389 | 2.63682 | 2.151749002 |
| CIDEA | 0.0539364 | 0.508278 | 3.236286651 |
| CLDN1 | 12.111 | 72.2147 | 2.575974549 |
| CLDN16 | 0.924302 | 4.24056 | 2.197818587 |
| CMAHP | 0.16801 | 0.681337 | 2.019821449 |
| CNIH3 | 0.422025 | 2.85051 | 2.755819693 |
| CNN1 | 5.86113 | 1.09683 | -2.4178389 |
| COL13A1 | 0.172302 | 0.77377 | 2.166965347 |
| COL26A1 | 0.588216 | 0.110695 | -2.409755969 |
| CORO2B | 0.176007 | 0.793621 | 2.172817394 |
| CPNE5 | 0.119867 | 0.574277 | 2.26031225 |
| CR1L | 0.193928 | 2.9651 | 3.934487737 |
| CRB2 | 4.11946 | 0.18765 | -4.456339039 |
| CSF1 | 10.3872 | 75.4493 | 2.860700706 |
| CSF2 | 0.355361 | 163.868 | 8.849033081 |
| CSF3 | 0.0340134 | 3.55643 | 6.708182732 |
| CTB-178M22.2 | 1.28879 | 0.232908 | -2.468185105 |
| CTB-7E3.1 | 0.804082 | 0.136495 | -2.55849453 |
| CTSE | 0 | 1.78839 | Infinite |
| CTSS | 2.85962 | 49.629 | 4.117288033 |
| CTSW | 0.264677 | 1.07099 | 2.016640272 |
| CXCL1 | 1.18888 | 165.136 | 7.117907751 |
| CXCL10 | 0.369584 | 45.5583 | 6.945667803 |
| CXCL11 | 0.443635 | 73.1218 | 7.364784585 |
| CXCL2 | 2.09241 | 64.8034 | 4.952832033 |
| CXCL3 | 0.395697 | 27.0914 | 6.097295012 |
| CXCL8 | 2.6259 | 330.975 | 6.977766462 |
| CXCR4 | 0.139241 | 0.665732 | 2.257357436 |
| CYP1B1 | 2.44576 | 12.1489 | 2.312470948 |
| CYP27B1 | 0.522781 | 2.68561 | 2.3609712 |
| CYP2J2 | 0.0997932 | 0.608934 | 2.609272451 |
| CYP4F2 | 0.987998 | 0.144504 | -2.773398693 |
| DAPP1 | 0.13724 | 0.693369 | 2.336922305 |
| DDO | 0.41695 | 3.19153 | 2.936301916 |
| DENND2A | 33.9888 | 8.30345 | -2.033276636 |
| DHDH | 0.715858 | 0.0723439 | -3.306730158 |
| DLL4 | 0.159243 | 0.756802 | 2.248685945 |
| DNASE1L2 | 1.45932 | 0.193971 | -2.911383392 |
| DNER | 0.165602 | 1.84055 | 3.474344941 |
| DNM3 | 0.789528 | 0.164125 | -2.26619542 |
| DOCK4 | 0.709157 | 7.84814 | 3.468173812 |
| DOCK9-AS1 | 0.210384 | 1.28593 | 2.611715216 |
| DOK7 | 2.00813 | 0.358677 | -2.485095527 |
| DUSP10 | 5.14645 | 24.6476 | 2.259797659 |
| DUSP5 | 33.0616 | 153.872 | 2.218502279 |
| DUSP6 | 0.702752 | 4.38098 | 2.640166069 |
| EBI3 | 1.51332 | 12.468 | 3.042441069 |
| EDIL3 | 89.9832 | 18.2989 | -2.297898747 |
| EDN2 | 18.667 | 0.997937 | -4.225397538 |
| EEF1DP3 | 2.75312 | 0.566398 | -2.281179418 |
| EFCAB4A | 1.30361 | 0.179929 | -2.857012688 |
| EFHD1 | 2.40829 | 0.252789 | -3.252003536 |
| ELTD1 | 0.102425 | 1.03934 | 3.343027883 |
| EMID1 | 0.591261 | 0.120689 | -2.292500929 |
| EMR1 | 0.0680329 | 1.0576 | 3.958417684 |
| ENTPD8 | 0.513995 | 0.0740993 | -2.794222506 |
| EPHA5 | 1.27091 | 0.310366 | -2.033819442 |
| EPHA5-AS1 | 1.58685 | 0.350319 | -2.179424619 |
| EPPK1 | 1.25646 | 0.291478 | -2.107905841 |
| EPS8L2 | 57.1864 | 13.6888 | -2.062676107 |
| ERG | 0.167144 | 1.07682 | 2.687613638 |
| ESM1 | 0.0749382 | 39.2336 | 9.03217268 |
| EVI2B | 0.201942 | 0.950489 | 2.234728936 |
| FABP6 | 0.229021 | 4.86689 | 4.409448371 |
| FADS2 | 90.8227 | 16.5031 | -2.460315875 |
| FAIM3 | 1.03281 | 26.5119 | 4.681993286 |
| FAM101A,ZNF664-FAM101A | 3.7569 | 24.1239 | 2.68284854 |
| FAM167A | 1.09894 | 10.7775 | 3.293838037 |
| FAM171A2 | 2.54384 | 0.425526 | -2.579688743 |
| FAM196B | 0.184417 | 3.59576 | 4.285253173 |
| FAM198B | 4.65999 | 0.558925 | -3.059600248 |
| FAT3 | 2.31167 | 0.375306 | -2.622796202 |
| FBXO32 | 1.40284 | 11.6486 | 3.053734196 |
| FCER1G | 0.153033 | 6.68026 | 5.44798956 |
| FDCSP | 0 | 2.02571 | Infinite |
| FGF18 | 2.54954 | 0.582983 | -2.128711253 |
| FGFR2 | 22.3702 | 1.39747 | -4.000688937 |
| FHDC1 | 1.79869 | 0.400612 | -2.166669021 |
| FKSG29 | 0.165271 | 0.901671 | 2.447767524 |
| FLJ36000 | 1.17767 | 0.0220514 | -5.738921269 |
| FOLR1 | 2.18511 | 0.119817 | -4.188801386 |
| FOSL1 | 13.2219 | 117.538 | 3.152125842 |
| FOXO6 | 1.72781 | 0.351858 | -2.295879359 |
| FOXQ1 | 0.149293 | 0.598249 | 2.002599558 |
| FOXS1 | 0.851775 | 0.128853 | -2.72474626 |
| FRMD6-AS2 | 0.222899 | 1.28504 | 2.527351218 |
| FRY | 1.87629 | 0.122072 | -3.9420786 |
| FST | 3.75499 | 16.0354 | 2.094379371 |
| G0S2 | 1.87938 | 129.321 | 6.104555957 |
| GAL | 0.271023 | 7.77231 | 4.84185625 |
| GAS6-AS1 | 0.70356 | 0.145494 | -2.273713803 |
| GBP1P1 | 0.0318296 | 1.54775 | 5.603659622 |
| GBP4 | 0.302645 | 12.9668 | 5.421052163 |
| GBP5 | 0.333496 | 5.38417 | 4.0129826 |
| GCHFR | 16.8094 | 4.06388 | -2.048338522 |
| GIMAP2 | 0.679151 | 3.77594 | 2.475031561 |
| GIPR | 0.555874 | 0.138273 | -2.007238429 |
| GLI1 | 1.35718 | 0.104841 | -3.69433715 |
| GLP2R | 2.38223 | 0.137291 | -4.117003751 |
| GNG7 | 0.979541 | 0.218657 | -2.163436345 |
| GNGT2 | 0.112706 | 1.09677 | 3.282624789 |
| GPC1 | 20.5861 | 4.18107 | -2.299726437 |
| GPR162 | 26.0881 | 3.81121 | -2.775070867 |
| GPR17 | 1.38721 | 0.256451 | -2.435431102 |
| GPRIN2 | 2.46417 | 0.389415 | -2.661721427 |
| GRAMD2 | 1.27906 | 0.275836 | -2.213201278 |
| GREM1 | 118.279 | 498.217 | 2.074580298 |
| HBEGF | 8.91297 | 36.9907 | 2.053184447 |
| HCK | 0.158347 | 1.24003 | 2.969213584 |
| HILS1 | 0.521114 | 0 | Infinite |
| HIST1H3H | 489.69 | 95.5387 | -2.357711584 |
| HIST1H4A | 213.626 | 47.2765 | -2.175892107 |
| HIST1H4F | 59.4108 | 11.2035 | -2.406775711 |
| HIST1H4J | 14.4842 | 3.51799 | -2.041656716 |
| HIST1H4K | 57.7015 | 7.33257 | -2.97621798 |
| HIST4H4 | 295.064 | 52.5327 | -2.489740271 |
| HLA-DQA1 | 0.0255259 | 1.39548 | 5.772655809 |
| HMGA2 | 10.6755 | 94.1056 | 3.139976935 |
| HMOX1 | 9.2481 | 42.391 | 2.196529097 |
| HOXC5 | 0.786828 | 0.174505 | -2.172779924 |
| HOXC-AS1 | 0.925295 | 0.0771992 | -3.583255593 |
| HS3ST3B1 | 0.0216519 | 0.555966 | 4.682431123 |
| HSPA6 | 0.455287 | 2.29185 | 2.331664453 |
| HTR7 | 0.231254 | 1.66978 | 2.852107809 |
| ICAM5 | 2.49042 | 0.560968 | -2.150398687 |
| IDO1 | 2.21286 | 39.6886 | 4.164740588 |
| IFIT2 | 61.2075 | 342.779 | 2.485498379 |
| IFNB1 | 0.694528 | 20.231 | 4.864390965 |
| IFNL1 | 0.28582 | 6.57667 | 4.524178505 |
| IFNL2 | 0.181799 | 7.09257 | 5.285892315 |
| IFNL3 | 0.174845 | 2.17216 | 3.63498193 |
| IGFBP5 | 0.5025 | 0.0989896 | -2.34377473 |
| IL11 | 1.87087 | 11.1012 | 2.568934416 |
| IL11RA | 4.68927 | 1.02513 | -2.193556475 |
| IL15RA | 7.17877 | 29.7205 | 2.049649806 |
| IL1A | 0.113224 | 4.12471 | 5.187040987 |
| IL1B | 0.103604 | 3.84556 | 5.214042093 |
| IL1R1 | 1.55959 | 7.15298 | 2.197377598 |
| IL1RAPL1 | 0.152882 | 1.02167 | 2.740438818 |
| IL24 | 0.713556 | 57.1297 | 6.323070487 |
| IL32 | 6.79623 | 41.1608 | 2.59846444 |
| IL4I1 | 0.479841 | 9.65991 | 4.331381409 |
| IL6 | 6.85459 | 325.02 | 5.567314311 |
| IL7 | 0.184381 | 0.739488 | 2.003836739 |
| IL7R | 7.72144 | 69.2296 | 3.164447181 |
| INHA | 3.124 | 0.267085 | -3.548023595 |
| INHBA | 0.860741 | 31.6145 | 5.198863402 |
| IRAK2 | 1.59142 | 16.7734 | 3.397788615 |
| ITGA2 | 1.39977 | 14.9758 | 3.419371375 |
| ITGA2B | 0.984227 | 0.180034 | -2.450721704 |
| ITGB4 | 8.13408 | 1.71915 | -2.242283751 |
| JMJD1C-AS1 | 1.4299 | 0.22519 | -2.666699587 |
| KBTBD8 | 0.24037 | 1.26938 | 2.400795264 |
| KCNH7 | 0.89343 | 0.109059 | -3.034245867 |
| KCNIP1 | 2.49902 | 0.213322 | -3.550257783 |
| KCNJ15 | 0.150562 | 1.11958 | 2.894528018 |
| KCNK15 | 0.605142 | 0.127578 | -2.245894152 |
| KCNK3 | 1.53584 | 6.64621 | 2.113503951 |
| KCTD19 | 0.996939 | 0.235669 | -2.080743234 |
| KHDC1L | 0.136389 | 0.590771 | 2.114871715 |
| KIAA0226L | 0.785764 | 0.0605531 | -3.697823347 |
| KRT17 | 5.08058 | 0.924687 | -2.457956193 |
| KRT34 | 1.02763 | 13.6633 | 3.732913151 |
| KRT4 | 1.68891 | 0.0116145 | -7.184021592 |
| KRT81 | 2.78931 | 12.3302 | 2.144216013 |
| KRTAP2-3 | 0.537615 | 6.56434 | 3.610004669 |
| KYNU | 0.0394899 | 3.01208 | 6.253132563 |
| LAMC2 | 14.7416 | 340.642 | 4.530291302 |
| LCE1B | 0.74569 | 0.0533057 | -3.80621428 |
| LCE1C | 1.0703 | 0.0636974 | -4.070636938 |
| LCN15 | 0.911342 | 0 | Infinite |
| LCP1 | 0.364753 | 2.12943 | 2.545475556 |
| LGI2 | 0.119007 | 0.966183 | 3.021250033 |
| LIME1 | 1.24498 | 0.269414 | -2.208225839 |
| LIMS2 | 3.8378 | 0.282931 | -3.761757367 |
| LINC00239 | 0.694789 | 0.0498556 | -3.800747445 |
| LINC00263 | 23.0091 | 4.2775 | -2.427364794 |
| LINC00460 | 0.250493 | 1.33785 | 2.417074177 |
| LINC00520 | 0.0810016 | 0.585145 | 2.852771861 |
| LINC00525 | 0.263577 | 1.30871 | 2.311849053 |
| LINC00592 | 0.608326 | 0.0656356 | -3.212294233 |
| LINC00941 | 1.16278 | 7.07893 | 2.605953147 |
| LINC01111 | 10.9407 | 2.47317 | -2.145271731 |
| LINC01119 | 0.478614 | 2.55519 | 2.416496069 |
| LINC01204 | 0.386963 | 2.0666 | 2.416991643 |
| LINC01260 | 0.14795 | 0.601155 | 2.022627323 |
| LINC01341 | 0.621331 | 0.148422 | -2.065657084 |
| LINC01358 | 0.972341 | 4.3565 | 2.163635282 |
| LINC-PINT | 3.64834 | 22.7726 | 2.641986927 |
| LIPG | 0.0580109 | 2.82271 | 5.604613104 |
| LLGL2 | 13.6709 | 2.6872 | -2.346932618 |
| LMO1 | 2.63977 | 0.489268 | -2.431715402 |
| LMO2 | 0.145658 | 1.36754 | 3.230926186 |
| LMOD1 | 0.648672 | 0.0647311 | -3.324958241 |
| LOC100127888 | 3.0112 | 15.3022 | 2.345328646 |
| LOC100128076 | 0.882148 | 0.133409 | -2.725164724 |
| LOC100130093 | 2.7326 | 0.628585 | -2.120094542 |
| LOC100130476 | 0.0828862 | 1.21184 | 3.869923499 |
| LOC100505474 | 0 | 0.555068 | Infinite |
| LOC100506499 | 0.217912 | 1.08628 | 2.317578471 |
| LOC100507144 | 0.0388459 | 0.784195 | 4.335378201 |
| LOC100996266 | 0.0351067 | 0.506373 | 3.850382186 |
| LOC101927497 | 1.0156 | 27.7066 | 4.769825477 |
| LOC101927642 | 1.57873 | 9.0476 | 2.518770691 |
| LOC101927780 | 0.155798 | 1.63516 | 3.391683191 |
| LOC101928417 | 0.0981845 | 0.541593 | 2.463641897 |
| LOC101928545 | 0.608557 | 0.108268 | -2.490785499 |
| LOC101929539 | 0.845888 | 0 | Infinite |
| LOC101929613 | 0.15287 | 1.46019 | 3.255778887 |
| LOC101929655 | 1.1531 | 0.111344 | -3.37242191 |
| LOC101929723 | 1.15541 | 6.24091 | 2.43335152 |
| LOC102031319 | 2.75558 | 0.395898 | -2.799155328 |
| LOC102724084 | 1.38869 | 0.281139 | -2.304369074 |
| LOC102724550 | 0.148534 | 1.52107 | 3.356221435 |
| LOC152225 | 0.101484 | 3.71884 | 5.195528484 |
| LOC389602 | 0.106772 | 1.46579 | 3.779073159 |
| LOC645638 | 28.1946 | 3.08627 | -3.19148269 |
| LOC653513 | 0.21038 | 0.918686 | 2.126574284 |
| LOC728463 | 3.06375 | 0.619533 | -2.306045543 |
| LOX | 2.24712 | 18.8193 | 3.066063893 |
| LPAR3 | 0.282633 | 1.41285 | 2.321606477 |
| LRRC2-AS1 | 0.157302 | 0.66156 | 2.072334993 |
| LRRC8C | 0.534477 | 2.51718 | 2.235608614 |
| LSP1 | 0.788746 | 0.0711807 | -3.470002758 |
| LURAP1L | 0.519955 | 2.14684 | 2.045755999 |
| LZTS3 | 5.47538 | 1.34369 | -2.026758758 |
| MAP1LC3C | 41.6576 | 0.587599 | -6.147603974 |
| MARCH4 | 0.294678 | 2.13872 | 2.859536357 |
| MCTP1 | 0.886595 | 3.62499 | 2.031629884 |
| MFSD2A | 4.64593 | 29.9664 | 2.689306454 |
| MGARP | 6.17252 | 1.30797 | -2.238530155 |
| MIR100HG | 0.933009 | 7.52611 | 3.011941473 |
| MIR103A2 | 26.5339 | 0 | Infinite |
| MIR103B2 | 205.721 | 0 | Infinite |
| MIR1178 | 0 | 4.85641 | Infinite |
| MIR1182 | 3.59119 | 0.00157045 | -11.15906826 |
| MIR1207 | 0 | 7.28431 | Infinite |
| MIR1224 | 0 | 5.21292 | Infinite |
| MIR1227 | 8.62761 | 0 | Infinite |
| MIR1229 | 0 | 15.8234 | Infinite |
| MIR1231 | 10.4932 | 0 | Infinite |
| MIR1260B | 12.4813 | 0 | Infinite |
| MIR1279 | 314.849 | 0 | Infinite |
| MIR1287 | 10.4528 | 0.000130123 | -16.29365394 |
| MIR1304 | 11.153 | 0 | Infinite |
| MIR130B | 0 | 32.2831 | Infinite |
| MIR1322 | 0 | 47.2498 | Infinite |
| MIR1324 | 8.07932 | 0 | Infinite |
| MIR135A1 | 0 | 20.841 | Infinite |
| MIR147B | 327.386 | 4902.64 | 3.904494294 |
| MIR150 | 0 | 51.0195 | Infinite |
| MIR155HG | 8.2497 | 51.0822 | 2.630407098 |
| MIR15A | 47.2911 | 0 | Infinite |
| MIR181B2 | 12.2844 | 0 | Infinite |
| MIR191 | 13.0607 | 0 | Infinite |
| MIR192 | 5.77371 | 0 | Infinite |
| MIR194-1 | 16.5081 | 0 | Infinite |
| MIR197 | 0 | 10.8069 | Infinite |
| MIR199A1 | 0 | 42.4831 | Infinite |
| MIR221 | 0 | 5.72433 | Infinite |
| MIR2278 | 14.206 | 0 | Infinite |
| MIR27B | 10.2988 | 0 | Infinite |
| MIR298 | 0 | 34.5826 | Infinite |
| MIR29C | 0 | 65.1418 | Infinite |
| MIR302C | 25.7186 | 0 | Infinite |
| MIR3074 | 0 | 24.9904 | Infinite |
| MIR30A | 0 | 39.3509 | Infinite |
| MIR3176 | 43.9872 | 0 | Infinite |
| MIR3189 | 0 | 77.0884 | Infinite |
| MIR3190 | 22.5441 | 0 | Infinite |
| MIR3192 | 17.6192 | 0 | Infinite |
| MIR320B2 | 2.41478 | 0 | Infinite |
| MIR329-1 | 11.7051 | 0 | Infinite |
| MIR34A | 5.95962 | 0 | Infinite |
| MIR3609 | 18.1274 | 0 | Infinite |
| MIR3619 | 19.3103 | 0 | Infinite |
| MIR3620 | 0 | 11.714 | Infinite |
| MIR3648 | 0.965413 | 0 | Infinite |
| MIR3650 | 107.179 | 0 | Infinite |
| MIR3653 | 6.301 | 2.65124E-10 | -34.46819448 |
| MIR3656 | 76.0139 | 0 | Infinite |
| MIR3671 | 14.6142 | 0 | Infinite |
| MIR3682 | 0 | 46.6665 | Infinite |
| MIR3689C | 0 | 18.4876 | Infinite |
| MIR3689D2 | 0 | 13.5317 | Infinite |
| MIR376B | 0 | 9.68464 | Infinite |
| MIR378J | 21.0736 | 2.39244 | -3.138882142 |
| MIR3909 | 3.60883 | 0 | Infinite |
| MIR3941 | 6.98609 | 0 | Infinite |
| MIR3942 | 5.84024 | 0 | Infinite |
| MIR3978 | 5.82313 | 0 | Infinite |
| MIR4269 | 18.2114 | 0 | Infinite |
| MIR4284 | 0 | 26.9353 | Infinite |
| MIR4292 | 92.3783 | 0 | Infinite |
| MIR4296 | 6.9911 | 0 | Infinite |
| MIR4312 | 10.5851 | 0 | Infinite |
| MIR433 | 0 | 11.2879 | Infinite |
| MIR4434 | 0 | 90.4902 | Infinite |
| MIR4439 | 0 | 19.0225 | Infinite |
| MIR4440 | 0 | 14.8071 | Infinite |
| MIR4441 | 13.2683 | 0 | Infinite |
| MIR4480 | 0 | 27.4845 | Infinite |
| MIR4505 | 0 | 27.386 | Infinite |
| MIR450A1 | 13.3929 | 0 | Infinite |
| MIR4516 | 0 | 10.7962 | Infinite |
| MIR452 | 16.8869 | 0 | Infinite |
| MIR454 | 4.25061 | 0 | Infinite |
| MIR4639 | 41.924 | 0 | Infinite |
| MIR4653 | 16.0658 | 0 | Infinite |
| MIR4658 | 56.2106 | 0 | Infinite |
| MIR4678 | 24.4662 | 0 | Infinite |
| MIR4685 | 44.7202 | 0 | Infinite |
| MIR4723 | 0 | 7.99896 | Infinite |
| MIR4728 | 30.6542 | 0 | Infinite |
| MIR4730 | 28.7834 | 0 | Infinite |
| MIR4742 | 76.8445 | 0 | Infinite |
| MIR4746 | 21.6241 | 0 | Infinite |
| MIR4755 | 70.4873 | 0 | Infinite |
| MIR4759 | 14.8663 | 0 | Infinite |
| MIR492 | 0 | 5.57963 | Infinite |
| MIR497 | 6.26701 | 0 | Infinite |
| MIR499A | 16.4991 | 0 | Infinite |
| MIR5006 | 2.39713 | 0 | Infinite |
| MIR505 | 0 | 8.63078 | Infinite |
| MIR5094 | 16.7616 | 0 | Infinite |
| MIR5193 | 0 | 1.34717 | Infinite |
| MIR5194 | 7.17231 | 0 | Infinite |
| MIR542 | 14.5492 | 0 | Infinite |
| MIR548D2 | 0 | 16.9379 | Infinite |
| MIR548F2 | 8.711 | 0 | Infinite |
| MIR548I1 | 1.89409 | 0 | Infinite |
| MIR548I2 | 0 | 2.75126 | Infinite |
| MIR553 | 0 | 90.4502 | Infinite |
| MIR554 | 9.36582 | 0 | Infinite |
| MIR5572 | 0 | 2.08364 | Infinite |
| MIR568 | 0 | 28.2662 | Infinite |
| MIR570 | 6.23922 | 35.3484 | 2.502207329 |
| MIR573 | 34.0404 | 8.02209 | -2.085197933 |
| MIR577 | 0 | 7.13819 | Infinite |
| MIR589 | 0 | 12.2719 | Infinite |
| MIR591 | 14.9126 | 0 | Infinite |
| MIR600 | 1.61006E-06 | 12.3351 | 22.86915162 |
| MIR601 | 24.7049 | 0 | Infinite |
| MIR6070 | 12.8052 | 0 | Infinite |
| MIR6080 | 293.772 | 0 | Infinite |
| MIR609 | 6.09036 | 0 | Infinite |
| MIR6124 | 3.29864 | 0 | Infinite |
| MIR6129 | 3.41488 | 0 | Infinite |
| MIR613 | 9.26529 | 0 | Infinite |
| MIR6133 | 0 | 15.124 | Infinite |
| MIR622 | 16.7041 | 0 | Infinite |
| MIR623 | 9.44093 | 0 | Infinite |
| MIR630 | 0 | 14.0568 | Infinite |
| MIR631 | 39.6105 | 0 | Infinite |
| MIR637 | 29.658 | 0 | Infinite |
| MIR641 | 109.559 | 5.19009 | -4.399804637 |
| MIR643 | 0 | 14.5325 | Infinite |
| MIR644A | 11.7502 | 0 | Infinite |
| MIR645 | 16.6703 | 0 | Infinite |
| MIR6505 | 33.4294 | 0 | Infinite |
| MIR6515 | 44.1115 | 0 | Infinite |
| MIR655 | 6.02463 | 0 | Infinite |
| MIR659 | 0 | 9.8647 | Infinite |
| MIR661 | 11.5545 | 0 | Infinite |
| MIR671 | 0.703699 | 1.16122E-06 | -19.20895761 |
| MIR6731 | 0 | 53.0004 | Infinite |
| MIR6733 | 65.486 | 0 | Infinite |
| MIR6738 | 29.8508 | 0 | Infinite |
| MIR6745 | 7.68352 | 0 | Infinite |
| MIR6747 | 31.1393 | 0 | Infinite |
| MIR6750 | 0 | 32.3787 | Infinite |
| MIR6757 | 49.8274 | 10.7812 | -2.20842153 |
| MIR6758 | 38.3937 | 0 | Infinite |
| MIR6761 | 0 | 71.2066 | Infinite |
| MIR6768 | 27.7579 | 0 | Infinite |
| MIR6772 | 0 | 33.8669 | Infinite |
| MIR6784 | 27.5733 | 0 | Infinite |
| MIR6796 | 37.9296 | 0 | Infinite |
| MIR6803 | 18.8281 | 0 | Infinite |
| MIR6810 | 21.1905 | 0 | Infinite |
| MIR6816 | 30.0862 | 0 | Infinite |
| MIR6817 | 0 | 39.3321 | Infinite |
| MIR6819 | 22.5989 | 0 | Infinite |
| MIR6837 | 0 | 48.8573 | Infinite |
| MIR6848 | 59.2383 | 0 | Infinite |
| MIR6849 | 13.1303 | 0 | Infinite |
| MIR6857 | 0 | 7.72807 | Infinite |
| MIR6858 | 25.8539 | 0 | Infinite |
| MIR6865 | 23.5543 | 0 | Infinite |
| MIR6878 | 39.1538 | 0 | Infinite |
| MIR6880 | 0 | 18.7007 | Infinite |
| MIR6892 | 8.93137 | 0 | Infinite |
| MIR708 | 0 | 15.9738 | Infinite |
| MIR7-1 | 0 | 9.21682 | Infinite |
| MIR765 | 3.86693 | 0 | Infinite |
| MIR8058 | 0 | 15.5951 | Infinite |
| MIR8083 | 12.8969 | 0 | Infinite |
| MIR922 | 5.7107E-152 | 52.0315 | 508.1207137 |
| MIR933 | 25.3256 | 0 | Infinite |
| MIR937 | 21.6188 | 0 | Infinite |
| MIR939 | 10.1877 | 0 | Infinite |
| MIR942 | 0 | 34.8698 | Infinite |
| MIR98 | 1.99713 | 0 | Infinite |
| MIR99A | 18.0057 | 0 | Infinite |
| MIRLET7G | 0 | 13.8776 | Infinite |
| MLXIPL | 0.600758 | 0.081715 | -2.878111119 |
| MME | 0.0374092 | 0.594048 | 3.989114489 |
| MMP1 | 1.30709 | 11.7855 | 3.172582579 |
| MMP3 | 0.174464 | 11.7992 | 6.079615864 |
| MMP9 | 0.416064 | 6.54612 | 3.97576268 |
| MOK | 5.80825 | 55.2458 | 3.249689333 |
| MORN3 | 0.51407 | 0.0824896 | -2.639680676 |
| MPP4 | 0.183463 | 2.64776 | 3.851211317 |
| MPZ | 0.157087 | 1.15915 | 2.883431573 |
| MRGPRX3 | 0.505243 | 0.0625925 | -3.012915724 |
| MSX1 | 0.473327 | 2.01714 | 2.091402093 |
| MTHFS | 0.599019 | 0.00680501 | -6.459860672 |
| MTSS1 | 0.617276 | 2.94382 | 2.253701856 |
| MTUS1 | 12.4449 | 2.99559 | -2.054642554 |
| MYEOV | 0.66204 | 4.5304 | 2.774648144 |
| MYH16 | 0.189834 | 2.28402 | 3.588764971 |
| MYL3 | 1.62345 | 0.0653738 | -4.634206583 |
| MYO7A | 2.40938 | 0.285428 | -3.077463176 |
| MYT1 | 0.655524 | 0.0866007 | -2.92019801 |
| NAMPT | 47.4768 | 214.065 | 2.172754328 |
| NAV3 | 0.571134 | 6.98013 | 3.611352729 |
| NCEH1 | 9.30857 | 37.6565 | 2.016267454 |
| NCF2 | 0.601925 | 28.8468 | 5.58268374 |
| NCOA7 | 6.4645 | 25.9807 | 2.006829608 |
| NDUFB7 | 54.9506 | 13.4764 | -2.027700078 |
| NDUFC2-KCTD14 | 1.96445 | 0.0789951 | -4.636218471 |
| NEURL1 | 1.01729 | 0.242174 | -2.070615119 |
| NEURL3 | 0.781115 | 12.9158 | 4.047458231 |
| NFE2 | 1.03625 | 0.181962 | -2.509663 |
| NFKBIA | 30.675 | 150.025 | 2.290067681 |
| NFKBIZ | 5.26058 | 32.4794 | 2.626231205 |
| NIPAL4 | 1.21791 | 6.55641 | 2.428498549 |
| NKAIN4 | 0.655657 | 0.0568744 | -3.527089956 |
| NLRP10 | 0.296906 | 1.77794 | 2.582128485 |
| NLRP3 | 0.363962 | 4.0493 | 3.475812795 |
| NMU | 38.4216 | 6.46076 | -2.57214181 |
| NOTCH3 | 0.505455 | 0.0944927 | -2.419307873 |
| NOTUM | 1.68483 | 0.208074 | -3.017434422 |
| NOXA1 | 0.530215 | 0.0669415 | -2.985604701 |
| NPNT | 17.9297 | 3.05071 | -2.5551344 |
| NPPB | 54.6328 | 9.86989 | -2.468661454 |
| NR2F1-AS1 | 0.285084 | 2.31451 | 3.021247819 |
| NR5A2 | 0.0273173 | 0.845031 | 4.951117466 |
| NRP2 | 2.16553 | 9.5704 | 2.143859065 |
| NT5E | 3.80296 | 153.787 | 5.337666979 |
| NTM | 1.21696 | 0.0911356 | -3.73912322 |
| OBSL1 | 78.4266 | 14.415 | -2.44377222 |
| ODF3B | 0.748779 | 0.0842708 | -3.151435248 |
| OR5H14 | 0 | 0.70181 | Infinite |
| PALM | 11.2241 | 1.87282 | -2.583315616 |
| PAQR9 | 0.187776 | 1.05347 | 2.488064644 |
| PC | 2.17589 | 0.464869 | -2.226709497 |
| PCOLCE | 6.2486 | 1.0727 | -2.542286333 |
| PCSK1N | 0.54222 | 0.109291 | -2.310703727 |
| PDCD1LG2 | 2.78554 | 32.9712 | 3.565177457 |
| PDZK1 | 9.88048 | 1.12124 | -3.139486012 |
| PDZK1IP1 | 0.121762 | 2.55887 | 4.393370989 |
| PHLDA1 | 6.27976 | 51.2058 | 3.027525903 |
| PHOSPHO2-KLHL23 | 0.651548 | 3.34519E-05 | -14.24949569 |
| PI3 | 0.17772 | 26.148 | 7.200950746 |
| PINLYP | 0.991116 | 5.20313 | 2.392253928 |
| PIP5KL1 | 0.935705 | 0.0449347 | -4.380151887 |
| PKP2 | 35.4949 | 7.71929 | -2.201071686 |
| PLA2G4B | 3.31987 | 0.799505 | -2.053947788 |
| PLAT | 16.9061 | 70.3723 | 2.057463777 |
| PLAU | 39.4481 | 347.168 | 3.137606254 |
| PLAUR | 16.4314 | 104.161 | 2.664287892 |
| PLCE1-AS1 | 11.9902 | 1.02707 | -3.545249306 |
| PLXNA2 | 0.17812 | 0.72626 | 2.027636606 |
| PP14571 | 0.731296 | 0.160994 | -2.183448549 |
| PPL | 13.2451 | 1.0477 | -3.660161158 |
| PPP4R4 | 0.321671 | 1.73871 | 2.434359543 |
| PRDM8 | 0.0388241 | 0.673148 | 4.115899348 |
| PRKAG2-AS1 | 0.204516 | 1.0016 | 2.292020848 |
| PRKCH | 0.268109 | 1.2274 | 2.194713934 |
| PRODH | 2.49178 | 0.404776 | -2.621981041 |
| PRR24 | 5.9441 | 1.41956 | -2.066014559 |
| PRSS27 | 0.509126 | 0.125392 | -2.021577436 |
| PSAT1 | 22.3547 | 114.068 | 2.351244033 |
| PSG2 | 0.119695 | 0.851499 | 2.830641948 |
| PTCD1 | 5.84548 | 0.819397 | -2.83468698 |
| PTGER2 | 0.519297 | 11.8767 | 4.515430331 |
| PTGER4 | 1.29458 | 6.97653 | 2.430025523 |
| PTGIR | 0 | 1.39204 | Infinite |
| PTPN22 | 0.53043 | 2.64923 | 2.320338822 |
| PTPRQ | 1.13886 | 0.0204981 | -5.795956407 |
| PTX3 | 1.60439 | 48.8078 | 4.92701494 |
| PZP | 2.4361 | 0.606213 | -2.00667666 |
| QSOX1 | 45.8359 | 195.928 | 2.095773681 |
| RARRES2 | 16.3041 | 1.35238 | -3.591662314 |
| RASA4 | 2.93699 | 0.554928 | -2.40396585 |
| RASAL1 | 3.68579 | 0.826657 | -2.156613125 |
| RASSF10 | 0.10753 | 0.526294 | 2.291129731 |
| RGMB-AS1 | 1.3298 | 6.49065 | 2.28715368 |
| RGS4 | 0.265313 | 3.54301 | 3.739208265 |
| RHBDL1 | 1.40735 | 0.281529 | -2.321625718 |
| RNF175 | 2.06066 | 0.487501 | -2.079629403 |
| RNU6-19P | 0 | 105.183 | Infinite |
| RNU86 | 602.733 | 108.373 | -2.475513686 |
| ROBO4 | 0.0397757 | 0.952138 | 4.581211463 |
| ROCK1P1 | 0.794021 | 0.188577 | -2.074023437 |
| RPLP0P2 | 0.856892 | 3.69848 | 2.109747186 |
| RPSAP52 | 0.370792 | 2.41778 | 2.705000953 |
| RTP4 | 16.1107 | 67.5675 | 2.068310297 |
| SAA1 | 10.6749 | 243.149 | 4.509546198 |
| SAA2 | 0.657116 | 24.0625 | 5.19449466 |
| SAA4 | 0.0332177 | 2.72265 | 6.35691554 |
| SAMD11 | 0.533082 | 0.0312858 | -4.090777569 |
| SAP25 | 0.891962 | 0.123035 | -2.857913469 |
| SCARA3 | 26.7536 | 5.41218 | -2.305451309 |
| SCART1 | 0.513578 | 0.0887165 | -2.533309048 |
| SCD | 101.442 | 19.5181 | -2.37777057 |
| SCG5 | 0.30447 | 6.19871 | 4.347596022 |
| SCN3A | 0.047287 | 1.40445 | 4.892417837 |
| SCN9A | 1.70942 | 0.237969 | -2.844661355 |
| SCNN1A | 2.60947 | 0.481621 | -2.437786612 |
| SEMA3B-AS1 | 1.80267 | 0.366194 | -2.29945526 |
| SEMA3C | 18.3604 | 124.295 | 2.759098868 |
| SEMA3E | 2.08111 | 0.227914 | -3.19079177 |
| SEPT5-GP1BB | 1.45623 | 0.26483 | -2.45909977 |
| SERPINA1 | 0.86727 | 31.1473 | 5.166482093 |
| SERPINB10 | 0 | 0.507119 | Infinite |
| SERPINB2 | 0.405688 | 82.3319 | 7.664937081 |
| SERPINB7 | 0.658636 | 39.1193 | 5.892255375 |
| SERPINB8 | 2.18797 | 14.8488 | 2.762681483 |
| SERPINE1 | 48.5465 | 428.942 | 3.143343393 |
| SERPINE2 | 2.13972 | 32.1565 | 3.909616463 |
| SGK1 | 2.64267 | 17.106 | 2.694434258 |
| SHF | 2.71485 | 0.650982 | -2.060182931 |
| SIGLEC15 | 0.388841 | 17.171 | 5.464649904 |
| SLC16A2 | 0.662112 | 4.04614 | 2.611399056 |
| SLC16A9 | 0.199387 | 1.15311 | 2.53188689 |
| SLC28A3 | 0.0630646 | 1.79677 | 4.832431529 |
| SLC29A2 | 4.10142 | 0.799934 | -2.35817061 |
| SLC29A4 | 1.35879 | 0.338606 | -2.004643063 |
| SLC38A4 | 1.02009 | 0.231648 | -2.138690312 |
| SLC40A1 | 3.73195 | 0.760881 | -2.294186915 |
| SLC43A3 | 0.217693 | 4.24553 | 4.285577744 |
| SLC44A5 | 0.125508 | 0.738052 | 2.55594314 |
| SLC6A15 | 1.138 | 4.93452 | 2.116409197 |
| SLCO4A1 | 11.0059 | 98.2118 | 3.157619247 |
| SLIT2 | 0.215205 | 1.37394 | 2.674535501 |
| SMIM1 | 1.80095 | 0.17868 | -3.333308063 |
| SMIM22 | 0.723676 | 0.109033 | -2.730579079 |
| SNAI3 | 0.578112 | 0.0842093 | -2.779297542 |
| SNAP25 | 5.4056 | 22.4949 | 2.057071288 |
| SNORA16B | 0 | 5.03774 | Infinite |
| SNORA36C | 3.03511 | 0 | Infinite |
| SNORA47 | 45.9437 | 4.59041 | -3.323172125 |
| SNORA56 | 5.2564E-132 | 2.52113 | 437.4345177 |
| SNORA70B | 0 | 7.42942 | Infinite |
| SNORA79 | 0 | 6.68022 | Infinite |
| SNORD105 | 16.4722 | 112.465 | 2.771370936 |
| SNORD111B | 8.22614 | 73.5694 | 3.160818295 |
| SNORD113-1 | 0 | 35.9599 | Infinite |
| SNORD113-6 | 0 | 23.223 | Infinite |
| SNORD114-14 | 60.3191 | 537.182 | 3.154724156 |
| SNORD114-20 | 0 | 36.1472 | Infinite |
| SNORD114-29 | 0 | 136.971 | Infinite |
| SNORD114-3 | 0 | 92.4601 | Infinite |
| SNORD114-31 | 0 | 24.5928 | Infinite |
| SNORD114-4 | 0 | 18.9051 | Infinite |
| SNORD114-7 | 77.6078 | 0 | Infinite |
| SNORD116-1 | 22.4171 | 0 | Infinite |
| SNORD116-10 | 0 | 16.4496 | Infinite |
| SNORD116-12 | 11.7261 | 0 | Infinite |
| SNORD116-13 | 60.7226 | 0 | Infinite |
| SNORD116-15 | 11.8647 | 0 | Infinite |
| SNORD116-2 | 10.0128 | 0 | Infinite |
| SNORD116-20 | 23.6962 | 0 | Infinite |
| SNORD116-26 | 0 | 23.8267 | Infinite |
| SNORD116-8 | 0 | 10.1904 | Infinite |
| SNORD11B | 0 | 24.0273 | Infinite |
| SNORD121B | 12.0676 | 0 | Infinite |
| SNORD123 | 24.4569 | 0 | Infinite |
| SNORD124 | 10.8984 | 0 | Infinite |
| SNORD125 | 6.17159E-19 | 27.1488 | 65.25380811 |
| SNORD16 | 24.6711 | 140.547 | 2.510158712 |
| SNORD18B | 26.46 | 0 | Infinite |
| SNORD18C | 0 | 77.5934 | Infinite |
| SNORD1B | 15.2867 | 75.1345 | 2.297198512 |
| SNORD20 | 18.053 | 0 | Infinite |
| SNORD23 | 7.08711 | 0 | Infinite |
| SNORD34 | 97.6702 | 0 | Infinite |
| SNORD42B | 165.331 | 36.0471 | -2.197402155 |
| SNORD48 | 27.8189 | 0 | Infinite |
| SNORD51 | 0 | 110.654 | Infinite |
| SNORD56 | 256.628 | 0 | Infinite |
| SNORD61 | 70.6943 | 0 | Infinite |
| SNORD64 | 0.654607 | 83.9171 | 7.002191982 |
| SNORD68 | 32.9002 | 207.211 | 2.654932333 |
| SNORD7 | 42.5869 | 9.33368 | -2.189891805 |
| SNORD85 | 0 | 60.1911 | Infinite |
| SNORD88B | 0 | 41.0505 | Infinite |
| SNORD90 | 5.19667 | 0 | Infinite |
| SNORD91B | 46.8564 | 369.774 | 2.980325767 |
| SNORD93 | 20.7881 | 350.835 | 4.076962872 |
| SNORD98 | 0 | 143.757 | Infinite |
| SNX15 | 5.52202 | 0.000309943 | -14.12090557 |
| SOCS2-AS1 | 0.150872 | 0.78417 | 2.377841366 |
| SOD2 | 38.0942 | 242.838 | 2.672350933 |
| SOGA3 | 0.255202 | 1.13423 | 2.15200168 |
| SOHLH2 | 0.039367 | 0.561257 | 3.833602853 |
| SORL1 | 2.07182 | 0.515338 | -2.007307784 |
| SOX9 | 2.52129 | 14.871 | 2.560267692 |
| SP140 | 2.16532 | 21.3933 | 3.304506888 |
| SPECC1L-ADORA2A | 1.56427 | 6.93022 | 2.147411602 |
| SPINK2 | 0.89119 | 0.128763 | -2.791014949 |
| SPOCD1 | 0.644172 | 5.03747 | 2.967181484 |
| SPOCK1 | 1.19496 | 5.74573 | 2.265527874 |
| SPOCK2 | 1.19544 | 0.0669182 | -4.158999272 |
| SPRY4 | 0.398554 | 4.56502 | 3.517774068 |
| SRCRB4D | 3.53869 | 0.657584 | -2.427968281 |
| SRD5A3-AS1 | 0.540912 | 2.96351 | 2.453841117 |
| SRGN | 0.457277 | 23.5937 | 5.689189515 |
| SRPK3 | 1.00074 | 0.198199 | -2.336045611 |
| SSTR2 | 0.774731 | 6.69196 | 3.110661448 |
| SSX1 | 4.59992 | 0.505309 | -3.18637099 |
| ST8SIA6 | 0.111651 | 1.545 | 3.79053876 |
| STARD8 | 0.383116 | 1.66833 | 2.122551503 |
| STC1 | 2.1016 | 12.3847 | 2.558998911 |
| STEAP1 | 0.4252 | 5.06886 | 3.575447816 |
| STMND1 | 0.597174 | 0.132467 | -2.172518352 |
| STON1 | 0.682038 | 0.151613 | -2.16945866 |
| SULT1E1 | 1.67634 | 0.19917 | -3.073242527 |
| SULT2B1 | 1.75995 | 0.0772036 | -4.510722511 |
| SUSD2 | 1.00109 | 0.214855 | -2.220136425 |
| SUSD4 | 0.259632 | 3.29327 | 3.664980683 |
| SYNPO | 4.87687 | 1.15296 | -2.080613054 |
| SYT12 | 1.17313 | 0.0452658 | -4.695797632 |
| SYT17 | 8.25998 | 1.36384 | -2.598463885 |
| TAC3 | 0.0711819 | 0.576536 | 3.017828348 |
| TBX15 | 0.152557 | 0.874484 | 2.519083609 |
| TCEA2 | 26.2825 | 5.9385 | -2.145932039 |
| TFPI2 | 4.80136 | 280.042 | 5.866056293 |
| TGFA | 5.53621 | 28.1449 | 2.345902949 |
| TGFB2 | 31.6633 | 7.74822 | -2.0308748 |
| TGM1 | 2.07058 | 0.462857 | -2.161396499 |
| TGM2 | 6.09907 | 123.496 | 4.33973123 |
| THBD | 1.45722 | 9.03025 | 2.631547228 |
| TIMP3 | 3.02619 | 12.1305 | 2.003064542 |
| TJP3 | 3.24925 | 0.575995 | -2.495978557 |
| TM4SF1 | 0.0237848 | 3.87187 | 7.34684681 |
| TMC3 | 0.68859 | 0.0728333 | -3.240975111 |
| TMC6 | 3.93394 | 0.892138 | -2.140636159 |
| TMEM154 | 0.432384 | 2.94352 | 2.767157387 |
| TMEM156 | 0.621507 | 3.9795 | 2.678744631 |
| TMEM158 | 5.12131 | 54.4624 | 3.410675764 |
| TMEM171 | 2.10645 | 13.807 | 2.712514307 |
| TMEM200A | 0.058853 | 8.85652 | 7.233480163 |
| TNC | 1.29963 | 8.16357 | 2.651099242 |
| TNFAIP3 | 1.77212 | 38.1694 | 4.428868303 |
| TNFAIP6 | 0.0129518 | 4.57381 | 8.464100013 |
| TNFRSF11B | 0.402688 | 3.48448 | 3.113208988 |
| TNFRSF1B | 3.45471 | 15.8031 | 2.193571075 |
| TNFRSF21 | 0.684373 | 3.76652 | 2.460377441 |
| TNFRSF8 | 0.158057 | 1.5491 | 3.292913443 |
| TNFRSF9 | 1.35093 | 9.09728 | 2.751482336 |
| TNFSF10 | 6.02702 | 89.4911 | 3.892227454 |
| TNFSF13B | 0.26398 | 18.5867 | 6.137698207 |
| TNNC1 | 76.2125 | 2.71301 | -4.81206137 |
| TNNT2 | 6.54038 | 0.146888 | -5.476586015 |
| TNNT3 | 0.591941 | 0.0157636 | -5.230784435 |
| TPPP3 | 0.558579 | 0.132576 | -2.074941705 |
| TRAF1 | 1.9834 | 43.0911 | 4.441342361 |
| TREX2 | 1.9743 | 0.352379 | -2.486141373 |
| TRIB1 | 1.23501 | 6.29249 | 2.349108295 |
| TRIM22 | 16.0393 | 68.5842 | 2.096265076 |
| TRIM36 | 0.185483 | 0.920255 | 2.310746717 |
| TRIM55 | 2.15406 | 8.83922 | 2.036860632 |
| TRIM74 | 0.715065 | 0.106567 | -2.746313634 |
| TSPAN12 | 11.5931 | 2.68457 | -2.110503467 |
| TSPAN18 | 0.146212 | 0.769536 | 2.395927098 |
| TTC9 | 2.60981 | 0.563929 | -2.210359339 |
| TTLL6 | 0.238547 | 1.01752 | 2.092711704 |
| UCN | 0.509677 | 0 | Infinite |
| UPB1 | 0.0361766 | 0.685544 | 4.244120535 |
| URGCP-MRPS24 | 0.0488694 | 9.04727 | 7.532407325 |
| VAX2 | 1.47318 | 0.340792 | -2.111970342 |
| VCAM1 | 0.025587 | 5.48416 | 7.743715846 |
| VEGFC | 8.23432 | 48.3142 | 2.552725853 |
| VGF | 0.846367 | 3.44627 | 2.025680452 |
| VNN1 | 0.00873363 | 1.01574 | 6.861734033 |
| VSTM1 | 2.03537 | 9.76452 | 2.262258048 |
| VTN | 4.09004 | 0.906075 | -2.174412574 |
| WBP1 | 20.966 | 3.72889 | -2.491233499 |
| WISP2 | 0.975463 | 0.0959223 | -3.346148995 |
| WNT9A | 0.571996 | 0.0489476 | -3.54669503 |
| WTAPP1 | 0.761681 | 0.115113 | -2.726136139 |
| XDH | 0.758309 | 4.21754 | 2.475544001 |
| XXYLT1-AS2 | 0.0919377 | 0.812044 | 3.142829421 |
| ZCCHC5 | 0.100098 | 0.579508 | 2.533415429 |
| ZNF385B | 0.510211 | 0.0591349 | -3.109012272 |
