# Supplementary material for: Identification of tumorigenesis-related mRNAs associated with RNA-binding protein HuR in thyroid cancer cells
Source: Oncotarget. 2016 Aug 12;7(39):63388–407. doi: 10.18632/oncotarget.11255 (PMC5325372; doi:10.18632/oncotarget.11255)
Supplement: Supplementary file 3 [file oncotarget-07-63388-s003.docx]

*Supplementary Table 2. BCPAP genes modified by HuR silencing*

| gene | CN FPKM | siRNA1 FPKM | Fold Change (log2) |
| --- | --- | --- | --- |
| ABCG1 | 3.57186 | 0.86536 | -2.045 |
| ADAM12 | 0.915828 | 4.60648 | 2.331 |
| ADIRF | 43.2213 | 5.88937 | -2.876 |
| AHNAK2 | 19.0908 | 4.23569 | -2.172 |
| AIM2 | 1.71697 | 10.0488 | 2.549 |
| ALOX5AP | 0.611019 | 4.01674 | 2.717 |
| ANGPTL4 | 4.15598 | 18.3485 | 2.142 |
| ARL14EPL | 19.0476 | 96.6016 | 2.342 |
| ASS1 | 9.79544 | 2.44239 | -2.004 |
| AURKC | 1.07632 | 4.55973 | 2.083 |
| BATF2 | 1.85342 | 7.97112 | 2.105 |
| BCL2A1 | 8.27193 | 45.1201 | 2.447 |
| BIRC3 | 7.99648 | 35.4618 | 2.149 |
| BLACAT1 | 3.34317 | 0.593181 | -2.495 |
| BLOC1S5-TXNDC5 | 3.05858 | 0.220519 | -3.794 |
| BTC | 0.49726 | 2.23783 | 2.170 |
| C15orf48 | 32.5528 | 151.691 | 2.220 |
| C1S | 0.33821 | 6.01402 | 4.152 |
| CAPN14 | 0.785558 | 0.182066 | -2.109 |
| CCDC144NL | 2.06393 | 9.44473 | 2.194 |
| CCL20 | 3.08679 | 36.6394 | 3.569 |
| CCL3 | 0.165378 | 4.02424 | 4.605 |
| CCL5 | 2.84772 | 20.834 | 2.871 |
| CCR4 | 0.012792 | 0.523017 | 5.354 |
| CD300C | 0.249205 | 1.22676 | 2.299 |
| CH25H | 0.8121 | 4.97647 | 2.615 |
| CHKB-CPT1B | 0.189269 | 0.828664 | 2.130 |
| CLDN3 | 0.567624 | 0 | Infinite |
| CLYBL | 0.742867 | 0.094341 | -2.977 |
| CMPK2 | 1.55802 | 54.9281 | 5.140 |
| CR1L | 0.055373 | 0.554168 | 3.323 |
| CSAG1 | 0.475491 | 2.48623 | 2.386 |
| CSF2 | 17.0138 | 76.3955 | 2.167 |
| CSF3 | 3.74644 | 20.1777 | 2.429 |
| CXCL10 | 0.576156 | 18.1624 | 4.978 |
| CXCL11 | 0.191901 | 14.1487 | 6.204 |
| CXCL2 | 20.6712 | 116.098 | 2.490 |
| CXCL3 | 7.87471 | 38.3015 | 2.282 |
| CXCL5 | 68.9654 | 371.096 | 2.428 |
| CXCL8 | 545.536 | 2303.27 | 2.078 |
| CYSLTR1 | 5.27178 | 1.25131 | -2.075 |
| DAPP1 | 0.204811 | 0.820331 | 2.002 |
| DBP | 12.3533 | 2.25822 | -2.452 |
| DDX26B-AS1 | 0 | 0.608756 | Infinite |
| DDX58 | 7.18685 | 37.4908 | 2.383 |
| DDX60 | 17.0816 | 87.4903 | 2.357 |
| DDX60L | 3.81638 | 18.7814 | 2.299 |
| DHRS2 | 3.24542 | 98.8791 | 4.929 |
| EEF1A1 | 0 | 1884.3 | Infinite |
| EEF1E1-BLOC1S5 | 1.14E-07 | 0.856014 | 22.837 |
| EPSTI1 | 0.2939 | 9.31949 | 4.987 |
| ESM1 | 26.5175 | 126.448 | 2.254 |
| ETV7 | 0.916517 | 7.40492 | 3.014 |
| FAM196B | 0.42732 | 1.99406 | 2.222 |
| FAM25A | 1.13478 | 0 | Infinite |
| FAM9B | 0.107203 | 0.516186 | 2.268 |
| FGFBP1 | 2.46074 | 0.3766 | -2.708 |
| FRMPD4 | 0.148121 | 0.757653 | 2.355 |
| G0S2 | 37.13 | 162.576 | 2.130 |
| GBP1 | 11.1684 | 45.1814 | 2.016 |
| GBP1P1 | 0.200713 | 1.98186 | 3.304 |
| GBP5 | 0.762629 | 11.5259 | 3.918 |
| GNG8 | 0 | 1.24169 | Infinite |
| HERC6 | 4.22161 | 32.4434 | 2.942 |
| HIST1H1T | 0.036823 | 0.593448 | 4.010 |
| HIST1H2AG | 589.509 | 143.594 | -2.038 |
| HIST1H2AH | 459.027 | 114.478 | -2.004 |
| HIST1H2AI | 582.889 | 136.087 | -2.099 |
| HIST1H2BA | 0.185949 | 1.78488 | 3.263 |
| HIST1H4D | 878.245 | 179.857 | -2.288 |
| HIST1H4G | 0 | 0.532946 | Infinite |
| HIST1H4I | 147.446 | 33.5251 | -2.137 |
| HIST1H4J | 15.8121 | 3.75564 | -2.074 |
| HRASLS2 | 0.154004 | 0.861737 | 2.484 |
| HSH2D | 0.959366 | 7.97311 | 3.055 |
| IFI27 | 424.867 | 2733.98 | 2.686 |
| IFI44L | 5.28199 | 101.565 | 4.265 |
| IFI6 | 58.4661 | 2663.46 | 5.510 |
| IFIH1 | 9.39767 | 48.6271 | 2.371 |
| IFIT1 | 7.99551 | 140.548 | 4.136 |
| IFIT2 | 8.02075 | 52.7916 | 2.719 |
| IFIT3 | 22.009 | 252.818 | 3.522 |
| IFITM1 | 83.1673 | 1225.23 | 3.881 |
| IFITM3 | 299.982 | 1208.31 | 2.010 |
| IFNB1 | 0 | 1.61468 | Infinite |
| IFNL1 | 0 | 2.12471 | Infinite |
| IFNL2 | 0 | 1.24617 | Infinite |
| IL23A | 1.8409 | 8.7793 | 2.254 |
| IL24 | 6.41025 | 28.2767 | 2.141 |
| IL36G | 0.630183 | 3.47719 | 2.464 |
| IRF9 | 14.5807 | 68.8436 | 2.239 |
| ISG15 | 26.9468 | 375.076 | 3.799 |
| ITGB4 | 2.79917 | 0.62633 | -2.160 |
| IVL | 0.639804 | 3.66231 | 2.517 |
| KCTD14 | 2.11327 | 19.1713 | 3.181 |
| KISS1 | 0 | 0.723924 | Infinite |
| KRT19 | 2.08293 | 0.503911 | -2.047 |
| LAMP3 | 0.862205 | 5.65372 | 2.713 |
| LGALS9 | 0.114484 | 3.92563 | 5.100 |
| LINC00161 | 0.040275 | 0.544205 | 3.756 |
| LINC00607 | 0.127891 | 0.513294 | 2.005 |
| LINC00911 | 0.214686 | 1.32687 | 2.628 |
| LINC01260 | 0.410316 | 2.10884 | 2.362 |
| LMO2 | 0.041109 | 0.544214 | 3.727 |
| LOC100188947 | 0.357198 | 1.45212 | 2.023 |
| LOC101927571 | 0.104874 | 0.799895 | 2.931 |
| LOC101927769 | 0.173059 | 0.758223 | 2.131 |
| LOC101927865 | 0.157154 | 0.712638 | 2.181 |
| LOC101928122 | 0.332274 | 4.46817 | 3.749 |
| LOC101929217 | 0.126298 | 0.70157 | 2.474 |
| LOC101929505 | 0.134755 | 0.724592 | 2.427 |
| LOC154872 | 0.135316 | 0.577547 | 2.094 |
| LOC643201 | 0.209875 | 3.14308 | 3.905 |
| LRRC38 | 5.15689 | 35.7485 | 2.793 |
| MAGEB6 | 0.107808 | 0.783911 | 2.862 |
| MIR100 | 0 | 20.6199 | Infinite |
| MIR103A2 | 25.5209 | 0 | Infinite |
| MIR106B | 0 | 17.8395 | Infinite |
| MIR10A | 7.72137 | 0 | Infinite |
| MIR1182 | 0 | 10.147 | Infinite |
| MIR1226 | 0 | 19.3593 | Infinite |
| MIR1227 | 9.44447 | 0 | Infinite |
| MIR1301 | 9.88131 | 0 | Infinite |
| MIR1304 | 0 | 10.8409 | Infinite |
| MIR130B | 0 | 21.4794 | Infinite |
| MIR1322 | 0 | 39.2097 | Infinite |
| MIR135A1 | 0 | 12.8539 | Infinite |
| MIR135A2 | 7.66949 | 0 | Infinite |
| MIR142 | 11.3619 | 0 | Infinite |
| MIR143HG | 0.589862 | 0.045255 | -3.704 |
| MIR146A | 14.7154 | 0 | Infinite |
| MIR147B | 531.519 | 2433.04 | 2.195 |
| MIR17 | 0 | 5.12914 | Infinite |
| MIR181B1 | 11.0349 | 0 | Infinite |
| MIR181B2 | 11.5108 | 0 | Infinite |
| MIR182 | 0 | 4.47617 | Infinite |
| MIR1909 | 0 | 20.9626 | Infinite |
| MIR195 | 0 | 15.8965 | Infinite |
| MIR196A1 | 31.7281 | 0 | Infinite |
| MIR1972-1 | 19.2111 | 0 | Infinite |
| MIR199A1 | 0 | 29.7053 | Infinite |
| MIR2278 | 0 | 6.74268 | Infinite |
| MIR2682 | 12.9952 | 0 | Infinite |
| MIR27A | 20.5223 | 0 | Infinite |
| MIR298 | 9.29533 | 0 | Infinite |
| MIR302C | 0 | 25.6116 | Infinite |
| MIR31 | 0 | 31.5261 | Infinite |
| MIR3136 | 12.9168 | 0 | Infinite |
| MIR3142 | 0 | 17.3699 | Infinite |
| MIR3143 | 75.4561 | 0 | Infinite |
| MIR3157 | 16.3502 | 0 | Infinite |
| MIR3176 | 0 | 13.6445 | Infinite |
| MIR320C1 | 0 | 11.2893 | Infinite |
| MIR330 | 0 | 9.0069 | Infinite |
| MIR345 | 8.48498 | 0 | Infinite |
| MIR3656 | 40.14 | 0 | Infinite |
| MIR3661 | 48.3699 | 10.3829 | -2.220 |
| MIR3662 | 26.641 | 0 | Infinite |
| MIR3685 | 0 | 114.021 | Infinite |
| MIR3687 | 0 | 85.517 | Infinite |
| MIR3689B | 0 | 1.68354 | Infinite |
| MIR3689C | 0 | 16.0406 | Infinite |
| MIR3689D2 | 0 | 11.3275 | Infinite |
| MIR3689E | 0 | 15.9033 | Infinite |
| MIR378E | 0 | 15.14 | Infinite |
| MIR3918 | 0 | 48.8171 | Infinite |
| MIR3935 | 0 | 4.8789 | Infinite |
| MIR3939 | 0 | 5.27765 | Infinite |
| MIR3940 | 0 | 4.58093 | Infinite |
| MIR3942 | 17.2058 | 0 | Infinite |
| MIR3975 | 28.4401 | 0 | Infinite |
| MIR423 | 0 | 7.63536 | Infinite |
| MIR4271 | 0 | 37.6396 | Infinite |
| MIR4305 | 0 | 4.96876 | Infinite |
| MIR431 | 56.003 | 13.7069 | -2.031 |
| MIR4319 | 15.1244 | 0 | Infinite |
| MIR4439 | 0 | 14.4518 | Infinite |
| MIR4441 | 7.85461 | 0 | Infinite |
| MIR4453 | 0 | 5.87261 | Infinite |
| MIR4458 | 8.26E-05 | 12.3531 | 17.191 |
| MIR4489 | 49.091 | 0 | Infinite |
| MIR4505 | 0 | 44.8466 | Infinite |
| MIR450A2 | 0 | 14.4998 | Infinite |
| MIR4516 | 10.6995 | 0 | Infinite |
| MIR454 | 0 | 4.07835 | Infinite |
| MIR4636 | 17.9029 | 0 | Infinite |
| MIR4644 | 0 | 13.3356 | Infinite |
| MIR4649 | 20.126 | 0 | Infinite |
| MIR4678 | 41.7876 | 0 | Infinite |
| MIR4685 | 20.0848 | 0 | Infinite |
| MIR4712 | 13.9822 | 0 | Infinite |
| MIR4725 | 0 | 13.0625 | Infinite |
| MIR4742 | 62.2667 | 12.5658 | -2.309 |
| MIR4761 | 36.4783 | 0 | Infinite |
| MIR4784 | 0 | 31.2591 | Infinite |
| MIR4793 | 1.77E-07 | 14.9313 | 26.331 |
| MIR499B | 0 | 25.3103 | Infinite |
| MIR5000 | 0 | 9.99315 | Infinite |
| MIR5087 | 0 | 17.2164 | Infinite |
| MIR5189 | 0 | 3.71696 | Infinite |
| MIR5191 | 0 | 3.47902 | Infinite |
| MIR5193 | 0 | 3.6848 | Infinite |
| MIR548H2 | 0 | 14.8786 | Infinite |
| MIR553 | 0 | 35.2875 | Infinite |
| MIR5572 | 1.78875 | 0 | Infinite |
| MIR5579 | 0 | 83.2815 | Infinite |
| MIR558 | 0 | 6.4579 | Infinite |
| MIR567 | 7.80215 | 0 | Infinite |
| MIR568 | 9.04164 | 0 | Infinite |
| MIR569 | 7.29154 | 0 | Infinite |
| MIR573 | 12.2478 | 0 | Infinite |
| MIR589 | 0 | 8.87214 | Infinite |
| MIR604 | 0 | 10.8352 | Infinite |
| MIR6081 | 9.8037 | 0 | Infinite |
| MIR6124 | 0 | 7.64755 | Infinite |
| MIR6129 | 0 | 3.32076 | Infinite |
| MIR6130 | 0 | 6.20248 | Infinite |
| MIR621 | 14.5871 | 1.67E-26 | -89.494 |
| MIR631 | 0 | 19.8916 | Infinite |
| MIR635 | 0 | 14.4956 | Infinite |
| MIR647 | 19.2408 | 0 | Infinite |
| MIR648 | 0 | 6.73409 | Infinite |
| MIR6513 | 45.2367 | 0 | Infinite |
| MIR6514 | 0 | 105.755 | Infinite |
| MIR6515 | 0 | 88.7754 | Infinite |
| MIR6730 | 0 | 14.9946 | Infinite |
| MIR6739 | 0 | 23.0445 | Infinite |
| MIR6750 | 79.3763 | 0 | Infinite |
| MIR6756 | 0 | 19.4048 | Infinite |
| MIR6757 | 22.1283 | 0 | Infinite |
| MIR6759 | 136.765 | 22.7385 | -2.588 |
| MIR6768 | 28.972 | 0 | Infinite |
| MIR6780B | 15.6883 | 118.334 | 2.915 |
| MIR6784 | 0 | 28.244 | Infinite |
| MIR6785 | 7.8709 | 0 | Infinite |
| MIR6791 | 64.2395 | 0 | Infinite |
| MIR6805 | 0 | 17.4009 | Infinite |
| MIR6809 | 0 | 8.19108 | Infinite |
| MIR6814 | 34.4412 | 0 | Infinite |
| MIR6819 | 20.8195 | 0 | Infinite |
| MIR6820 | 0 | 51.8409 | Infinite |
| MIR6826 | 0 | 10.0408 | Infinite |
| MIR6827 | 0 | 49.143 | Infinite |
| MIR6831 | 15.4652 | 0 | Infinite |
| MIR6845 | 0 | 167.733 | Infinite |
| MIR6847 | 0 | 25.7414 | Infinite |
| MIR6848 | 19.4798 | 0 | Infinite |
| MIR6849 | 0 | 13.4301 | Infinite |
| MIR6853 | 0 | 16.6746 | Infinite |
| MIR6856 | 60.8781 | 0 | Infinite |
| MIR6864 | 21.7125 | 0 | Infinite |
| MIR6867 | 0 | 21.7569 | Infinite |
| MIR6875 | 0 | 38.9134 | Infinite |
| MIR6892 | 0 | 4.80768 | Infinite |
| MIR6894 | 92.2197 | 0 | Infinite |
| MIR711 | 0 | 16.2799 | Infinite |
| MIR7110 | 7.27895 | 0 | Infinite |
| MIR7113 | 60.1395 | 0 | Infinite |
| MIR7114 | 0 | 38.7836 | Infinite |
| MIR758 | 0 | 12.7403 | Infinite |
| MIR765 | 6.58279 | 0 | Infinite |
| MIR7703 | 0 | 9.93849 | Infinite |
| MIR8055 | 9.05038 | 0 | Infinite |
| MIR8058 | 0 | 39.6419 | Infinite |
| MIR8064 | 0 | 14.1058 | Infinite |
| MIR8076 | 11.7897 | 0 | Infinite |
| MIR8085 | 13.3413 | 0 | Infinite |
| MIR922 | 4.89E-237 | 54.8374 | 790.785 |
| MIR936 | 0 | 5.50491 | Infinite |
| MIR937 | 11.9976 | 55.6145 | 2.213 |
| MIR938 | 0 | 17.1834 | Infinite |
| MIR940 | 9.20876 | 0 | Infinite |
| MIR941-2,MIR941-3 | 5.45884 | 0 | Infinite |
| MIR98 | 3.20991 | 0 | Infinite |
| MIRLET7G | 0 | 10.9747 | Infinite |
| MIRLET7I | 83.5605 | 0 | Infinite |
| MMP13 | 2.69214 | 29.6309 | 3.460 |
| MMP7 | 0.116574 | 0.663613 | 2.509 |
| MMP9 | 1.09821 | 6.26319 | 2.512 |
| MT1JP | 0.532431 | 0 | Infinite |
| MTHFS | 7.02088 | 0.542058 | -3.695 |
| MUC13 | 0.125937 | 1.28657 | 3.353 |
| MX1 | 9.46166 | 328.607 | 5.118 |
| MX2 | 11.736 | 68.6083 | 2.547 |
| MYOCD | 0.878656 | 4.29521 | 2.289 |
| NDUFC2-KCTD14 | 20.5079 | 4.99312 | -2.038 |
| NKAIN4 | 1.72663 | 0.221773 | -2.961 |
| NLRP10 | 0.10739 | 0.642186 | 2.580 |
| NME1-NME2 | 3.55268 | 0.589233 | -2.592 |
| NOX3 | 0.986019 | 0.217595 | -2.180 |
| NOXO1 | 0.152254 | 0.719655 | 2.241 |
| NPIPA5 | 0.178342 | 0.804539 | 2.174 |
| NUDT3 | 1.98697 | 0.285241 | -2.800 |
| OAS1 | 15.9326 | 236.574 | 3.892 |
| OAS2 | 6.90686 | 169.683 | 4.619 |
| OAS3 | 16.2752 | 123.836 | 2.928 |
| OASL | 14.0237 | 73.6695 | 2.393 |
| ODAM | 0.750432 | 0.147645 | -2.346 |
| OR51M1 | 0.132506 | 0.615376 | 2.215 |
| P2RX6P | 0 | 3.72641 | Infinite |
| PAGE2B | 0.142846 | 0.973138 | 2.768 |
| PARP9 | 4.78161 | 55.8251 | 3.545 |
| PATL2 | 0.283907 | 1.24093 | 2.128 |
| PCP2 | 1.88174 | 0.379863 | -2.309 |
| PI3 | 3.18439 | 19.3912 | 2.606 |
| PLEKHA4 | 0.150654 | 1.43933 | 3.256 |
| PLSCR1 | 16.7094 | 133.603 | 2.999 |
| POC1B-GALNT4 | 0.179998 | 0.769401 | 2.096 |
| PPL | 10.5067 | 1.23099 | -3.093 |
| PPP1R14A | 0.616519 | 0 | Infinite |
| PPP1R36 | 0.352727 | 1.4161 | 2.005 |
| PRDM1 | 0.473027 | 2.25116 | 2.251 |
| PSAT1 | 8.33774 | 38.0789 | 2.191 |
| PSG9 | 0.577534 | 3.02632 | 2.390 |
| RAB40A | 0.263177 | 1.12193 | 2.092 |
| RASGRP3 | 0.073142 | 1.87529 | 4.680 |
| RCAN2 | 0.506059 | 0.104156 | -2.281 |
| RGS9 | 1.88271 | 0.273263 | -2.784 |
| RIIAD1 | 0.520255 | 0 | Infinite |
| RNASE1 | 0.140822 | 0.598766 | 2.088 |
| RND3 | 60.6205 | 305.159 | 2.332 |
| RNF103-CHMP3 | 0.845336 | 0.139154 | -2.603 |
| RNU11 | 107.092 | 26.5952 | -2.010 |
| RNU5D-1 | 61.2635 | 0 | Infinite |
| RNU86 | 888.506 | 85.4113 | -3.379 |
| RPPH1 | 8887.65 | 1937.37 | -2.198 |
| RSAD2 | 4.94353 | 50.1809 | 3.344 |
| RTP4 | 1.91276 | 17.6089 | 3.203 |
| SAMD9 | 4.34336 | 32.347 | 2.897 |
| SAMD9L | 1.73993 | 16.3553 | 3.233 |
| SAMSN1 | 0.118897 | 0.974293 | 3.035 |
| SCARNA10 | 420.286 | 92.3966 | -2.185 |
| SCARNA11 | 0 | 2.17693 | Infinite |
| SERPINB2 | 4.7651 | 33.7411 | 2.824 |
| SLC26A9 | 0.155351 | 0.765372 | 2.301 |
| SLC38A5 | 0.157765 | 0.751223 | 2.251 |
| SLITRK6 | 1.57783 | 0.268795 | -2.553 |
| SMR3B | 47.271 | 10.3007 | -2.198 |
| SNORA19 | 15.4882 | 3.31686 | -2.223 |
| SNORA47 | 15.7049 | 0 | Infinite |
| SNORA56 | 7.17E-272 | 0.696548 | 900.200 |
| SNORA70E | 0 | 12.3063 | Infinite |
| SNORA70F | 2.46831 | 0 | Infinite |
| SNORA75 | 29.3747 | 4.84702 | -2.599 |
| SNORA76A | 2.57398 | 12.6552 | 2.298 |
| SNORD105 | 0 | 15.9101 | Infinite |
| SNORD105B | 0 | 46.3713 | Infinite |
| SNORD107 | 0.014724 | 1.54041 | 6.709 |
| SNORD114-14 | 28.3383 | 122.797 | 2.115 |
| SNORD114-21 | 0 | 83.8106 | Infinite |
| SNORD114-22 | 0 | 33.9933 | Infinite |
| SNORD114-23 | 0 | 30.936 | Infinite |
| SNORD114-24 | 0 | 31.2285 | Infinite |
| SNORD116-1 | 0 | 7.85975 | Infinite |
| SNORD116-10 | 0 | 6.27678 | Infinite |
| SNORD116-13 | 0 | 11.9501 | Infinite |
| SNORD116-2 | 9.29975 | 0 | Infinite |
| SNORD116-23 | 0 | 21.651 | Infinite |
| SNORD116-24 | 21.3899 | 0 | Infinite |
| SNORD116-29 | 15.5772 | 0 | Infinite |
| SNORD116-6 | 0 | 8.64291 | Infinite |
| SNORD116-8 | 24.9725 | 0 | Infinite |
| SNORD11B | 0 | 5.95041 | Infinite |
| SNORD19 | 0 | 81.5795 | Infinite |
| SNORD1B | 0 | 14.7074 | Infinite |
| SNORD20 | 0 | 18.6808 | Infinite |
| SNORD23 | 8.07816 | 0 | Infinite |
| SNORD30 | 34.8221 | 180.376 | 2.373 |
| SNORD31 | 41.7501 | 171.906 | 2.042 |
| SNORD41 | 0 | 29.5851 | Infinite |
| SNORD43 | 59.3912 | 264.871 | 2.157 |
| SNORD45B | 23.8422 | 134.45 | 2.495 |
| SNORD4B | 121.71 | 0 | Infinite |
| SNORD51 | 0 | 85.7304 | Infinite |
| SNORD53 | 103.988 | 0 | Infinite |
| SNORD56 | 102.7 | 0 | Infinite |
| SNORD59B | 53.6361 | 231.371 | 2.109 |
| SNORD64 | 1.23E-11 | 199.661 | 43.880 |
| SNORD67 | 12.275 | 0 | Infinite |
| SNORD7 | 0 | 25.4416 | Infinite |
| SNORD70 | 0 | 54.9986 | Infinite |
| SNORD77 | 259.163 | 55.3123 | -2.228 |
| SNORD90 | 0 | 5.01308 | Infinite |
| SNORD98 | 32.0836 | 0 | Infinite |
| SPRR2D | 0.772065 | 4.48546 | 2.538 |
| STAT1 | 51.4228 | 276.339 | 2.426 |
| SULT1E1 | 0.538134 | 0.100378 | -2.423 |
| SYNDIG1 | 0.641182 | 0.13579 | -2.239 |
| TM7SF2 | 13.6908 | 3.28164 | -2.061 |
| TMEM215 | 0.591632 | 0.124512 | -2.248 |
| TNFSF13B | 0.424893 | 7.03268 | 4.049 |
| TRAF1 | 1.64323 | 13.378 | 3.025 |
| TRIM22 | 2.82232 | 34.7208 | 3.621 |
| TRPV3 | 0.165017 | 0.945918 | 2.519 |
| TXNIP | 12.2637 | 56.3798 | 2.201 |
| UBXN10-AS1 | 0.89694 | 0.188507 | -2.250 |
| URGCP-MRPS24 | 0.045204 | 2.09423 | 5.534 |
| USP18 | 3.0109 | 45.3325 | 3.912 |
| USP30-AS1 | 0.334488 | 1.91527 | 2.518 |
| VTRNA1-2 | 0 | 14.31 | Infinite |
| VTRNA2-1 | 0 | 9.30402 | Infinite |
| XAF1 | 4.69242 | 64.863 | 3.789 |
| ZBP1 | 0.187323 | 4.97014 | 4.730 |
| ZP4 | 0 | 0.610854 | Infinite |
