# Supplementary material for: Identification of tumorigenesis-related mRNAs associated with RNA-binding protein HuR in thyroid cancer cells
Source: Oncotarget. 2016 Aug 12;7(39):63388–407. doi: 10.18632/oncotarget.11255 (PMC5325372; doi:10.18632/oncotarget.11255)
Supplement: Supplementary file 4 [file oncotarget-07-63388-s004.docx]

*Supplementary Table 3. Nthy-ori-3.1 and BCPAP common HuR interaction target RNA*

|  | Nthy-ori-3.1 | | | | BCPAP | | | |
| --- | --- | --- | --- | --- | --- | --- | --- | --- |
| gene | **HuR FPKM** | **IgG FPKM** | **Fold**  **Change**  **(log2)** | **q value** | **HuR FPKM** | **IgG FPKM** | **Fold**  **Change**  **(log2)** | **q value** |
| NECAB1 | 14.85 | 0.84 | 4.14 | 0.00029 | 22.86 | 0.78 | 4.87 | 0.00483 |
| EIF2S3 | 2081.24 | 160.15 | 3.70 | 0.00207 | 3220.20 | 148.81 | 4.44 | 0.00031 |
| UFM1 | 287.29 | 28.67 | 3.32 | 0.00029 | 388.11 | 18.98 | 4.35 | 0.00031 |
| LIN7C | 113.84 | 13.59 | 3.07 | 0.00029 | 349.86 | 19.51 | 4.16 | 0.00031 |
| FMR1 | 189.02 | 20.26 | 3.22 | 0.00029 | 153.25 | 9.80 | 3.97 | 0.00031 |
| CMC4 | 100.93 | 22.03 | 2.20 | 0.00029 | 33.75 | 2.16 | 3.96 | 0.00221 |
| RAB33B | 25.57 | 3.77 | 2.76 | 0.00029 | 49.52 | 3.25 | 3.93 | 0.00031 |
| SNHG16 | 1901.90 | 122.48 | 3.96 | 0.00029 | 2729.94 | 179.41 | 3.93 | 0.00031 |
| RAB21 | 117.25 | 14.22 | 3.04 | 0.00029 | 276.09 | 18.21 | 3.92 | 0.00031 |
| CXXC4 | 2.54 | 0.45 | 2.49 | 0.00029 | 6.39 | 0.42 | 3.91 | 0.00031 |
| DCUN1D1 | 99.24 | 16.74 | 2.57 | 0.00029 | 94.35 | 6.36 | 3.89 | 0.00031 |
| PAFAH1B2 | 324.49 | 34.19 | 3.25 | 0.00029 | 588.51 | 40.02 | 3.88 | 0.00031 |
| MARVELD2 | 22.00 | 4.32 | 2.35 | 0.00029 | 18.31 | 1.25 | 3.88 | 0.00031 |
| OSTF1 | 225.92 | 35.02 | 2.69 | 0.00029 | 119.05 | 8.20 | 3.86 | 0.00031 |
| SRSF6 | 763.21 | 56.05 | 3.77 | 0.00029 | 528.21 | 36.51 | 3.85 | 0.00031 |
| UBE2N | 358.16 | 24.91 | 3.85 | 0.00029 | 525.47 | 36.43 | 3.85 | 0.00031 |
| PTCHD4 | 5.17 | 0.87 | 2.57 | 0.00029 | 10.59 | 0.77 | 3.78 | 0.00031 |
| VMA21 | 311.41 | 37.33 | 3.06 | 0.00029 | 419.15 | 31.58 | 3.73 | 0.00031 |
| ZNF22 | 134.06 | 14.52 | 3.21 | 0.00029 | 164.82 | 12.49 | 3.72 | 0.00031 |
| ZNF625 | 27.47 | 5.73 | 2.26 | 0.00029 | 30.83 | 2.39 | 3.69 | 0.00031 |
| TMOD3 | 265.37 | 30.83 | 3.11 | 0.00029 | 224.51 | 17.59 | 3.67 | 0.00031 |
| FAM76A | 47.67 | 4.76 | 3.32 | 0.00029 | 36.92 | 2.91 | 3.66 | 0.00031 |
| TGFB3 | 3.94 | 0.31 | 3.66 | 0.00053 | 3.38 | 0.27 | 3.63 | 0.01835 |
| GRPEL2 | 125.14 | 13.39 | 3.22 | 0.00029 | 193.71 | 15.78 | 3.62 | 0.00031 |
| PDGFA | 70.40 | 4.39 | 4.00 | 0.00029 | 88.40 | 7.36 | 3.59 | 0.00031 |
| ALG10B | 14.54 | 2.93 | 2.31 | 0.00029 | 13.78 | 1.16 | 3.57 | 0.00031 |
| CD9 | 1060.24 | 89.21 | 3.57 | 0.00029 | 146.88 | 12.39 | 3.57 | 0.00031 |
| NAPG | 86.61 | 8.93 | 3.28 | 0.00029 | 133.97 | 11.39 | 3.56 | 0.00031 |
| PTBP2 | 35.03 | 7.81 | 2.17 | 0.00029 | 51.94 | 4.42 | 3.55 | 0.00031 |
| ANGEL2 | 55.81 | 9.04 | 2.63 | 0.00029 | 62.95 | 5.37 | 3.55 | 0.00031 |
| MPV17L | 4.11 | 0.96 | 2.10 | 0.00474 | 20.76 | 1.79 | 3.53 | 0.00031 |
| PNPO | 170.64 | 16.13 | 3.40 | 0.00029 | 183.05 | 16.00 | 3.52 | 0.00031 |
| PARD6B | 56.96 | 9.60 | 2.57 | 0.00029 | 99.79 | 8.77 | 3.51 | 0.00031 |
| PPP1CB | 911.44 | 67.94 | 3.75 | 0.00029 | 745.41 | 65.80 | 3.50 | 0.00031 |
| LYSMD1 | 47.96 | 5.75 | 3.06 | 0.00337 | 28.18 | 2.49 | 3.50 | 0.01846 |
| TOB1 | 92.95 | 10.99 | 3.08 | 0.00029 | 106.21 | 9.38 | 3.50 | 0.00031 |
| ZNF121 | 98.00 | 16.44 | 2.58 | 0.00029 | 311.26 | 27.56 | 3.50 | 0.00031 |
| WDR36 | 60.73 | 10.74 | 2.50 | 0.00029 | 155.96 | 13.89 | 3.49 | 0.00031 |
| DHRS3 | 362.36 | 30.36 | 3.58 | 0.00029 | 642.75 | 57.56 | 3.48 | 0.00031 |
| CDC42 | 1386.15 | 127.75 | 3.44 | 0.00029 | 1314.08 | 117.91 | 3.48 | 0.00031 |
| CRIPT | 70.06 | 9.96 | 2.81 | 0.00029 | 68.54 | 6.16 | 3.48 | 0.00031 |
| SLC30A7 | 47.83 | 8.13 | 2.56 | 0.00029 | 136.50 | 12.32 | 3.47 | 0.00031 |
| GPBP1L1 | 169.51 | 21.70 | 2.97 | 0.00029 | 268.08 | 24.33 | 3.46 | 0.00031 |
| BCL2L2-PABPN1 | 610.58 | 43.10 | 3.82 | 0.00029 | 553.49 | 50.24 | 3.46 | 0.00031 |
| CBFB | 193.81 | 21.39 | 3.18 | 0.00029 | 109.87 | 9.99 | 3.46 | 0.00031 |
| EBF1 | 3.33 | 0.58 | 2.52 | 0.00075 | 3.20 | 0.29 | 3.45 | 0.00308 |
| MARCKS | 250.52 | 24.35 | 3.36 | 0.00029 | 127.28 | 11.75 | 3.44 | 0.00031 |
| CMPK1 | 405.90 | 51.23 | 2.99 | 0.00029 | 513.33 | 47.42 | 3.44 | 0.00031 |
| SNRPF | 790.25 | 87.27 | 3.18 | 0.00029 | 718.22 | 66.97 | 3.42 | 0.00031 |
| HNRNPH1 | 1033.84 | 202.49 | 2.35 | 0.00029 | 933.87 | 87.55 | 3.42 | 0.00031 |
| TMEM64 | 44.52 | 7.08 | 2.65 | 0.00029 | 24.29 | 2.31 | 3.39 | 0.00031 |
| SAR1A | 534.61 | 96.51 | 2.47 | 0.00029 | 265.21 | 25.30 | 3.39 | 0.00031 |
| LCLAT1 | 87.66 | 11.31 | 2.95 | 0.00029 | 77.54 | 7.41 | 3.39 | 0.00031 |
| PDCL | 65.47 | 14.18 | 2.21 | 0.00029 | 36.29 | 3.48 | 3.38 | 0.00031 |
| SRRM1 | 266.33 | 23.10 | 3.53 | 0.00029 | 359.63 | 34.54 | 3.38 | 0.00031 |
| UGDH | 126.15 | 17.03 | 2.89 | 0.00029 | 220.75 | 21.68 | 3.35 | 0.00031 |
| ZNF280C | 19.82 | 3.96 | 2.32 | 0.00029 | 33.44 | 3.33 | 3.33 | 0.00031 |
| SIKE1 | 151.81 | 22.41 | 2.76 | 0.00029 | 104.32 | 10.40 | 3.33 | 0.00031 |
| SMS | 1023.01 | 134.26 | 2.93 | 0.00029 | 1154.07 | 115.13 | 3.33 | 0.00031 |
| MOB1B | 31.21 | 5.85 | 2.42 | 0.00029 | 55.24 | 5.54 | 3.32 | 0.00031 |
| TRAPPC2 | 88.63 | 9.80 | 3.18 | 0.00029 | 79.76 | 8.04 | 3.31 | 0.00031 |
| IFIT5 | 103.86 | 12.91 | 3.01 | 0.00029 | 45.16 | 4.59 | 3.30 | 0.00031 |
| TCP11L2 | 8.03 | 1.28 | 2.65 | 0.00029 | 119.42 | 12.19 | 3.29 | 0.00031 |
| TFPI | 58.95 | 9.09 | 2.70 | 0.00029 | 84.75 | 8.67 | 3.29 | 0.00031 |
| C3orf58 | 51.25 | 9.90 | 2.37 | 0.00029 | 19.86 | 2.04 | 3.29 | 0.00031 |
| DNAJA2 | 144.96 | 22.39 | 2.69 | 0.00029 | 156.76 | 16.07 | 3.29 | 0.00031 |
| ACTB | >10^6^ | 1628.45 | 9.26 | 1.00000 | 14675.0 | 1511.03 | 3.28 | 0.01310 |
| AFMID | 66.83 | 8.61 | 2.96 | 0.00029 | 55.79 | 5.76 | 3.28 | 0.00031 |
| PPP1R2 | 108.92 | 16.73 | 2.70 | 0.00029 | 135.77 | 14.09 | 3.27 | 0.00031 |
| TMED2 | 1010.18 | 103.11 | 3.29 | 0.00029 | 2107.31 | 218.76 | 3.27 | 0.00031 |
| EIF5A | 2431.89 | 192.42 | 3.66 | 0.00029 | 1689.15 | 175.95 | 3.26 | 0.00031 |
| RLIM | 83.23 | 20.33 | 2.03 | 0.00029 | 116.75 | 12.19 | 3.26 | 0.00031 |
| RAP2B | 55.74 | 4.57 | 3.61 | 0.00029 | 121.65 | 12.71 | 3.26 | 0.00031 |
| TBL1XR1 | 233.98 | 33.55 | 2.80 | 0.00029 | 253.14 | 26.46 | 3.26 | 0.00031 |
| NSUN6 | 28.61 | 6.80 | 2.07 | 0.00029 | 17.15 | 1.80 | 3.26 | 0.00031 |
| OGFRL1 | 102.24 | 22.75 | 2.17 | 0.00029 | 66.42 | 6.96 | 3.25 | 0.00031 |
| NUP62CL | 16.38 | 1.63 | 3.33 | 0.00029 | 3.95 | 0.41 | 3.25 | 0.01941 |
| CDC42SE2 | 73.17 | 9.64 | 2.92 | 0.00029 | 86.13 | 9.05 | 3.25 | 0.00031 |
| CCNI | 1238.52 | 65.75 | 4.24 | 0.00029 | 2284.69 | 241.44 | 3.24 | 0.00031 |
| TFAP4 | 105.83 | 6.80 | 3.96 | 0.00029 | 167.85 | 17.77 | 3.24 | 0.00031 |
| NABP1 | 40.26 | 6.87 | 2.55 | 0.00029 | 42.22 | 4.48 | 3.24 | 0.00031 |
| RSRC1 | 124.02 | 25.21 | 2.30 | 0.00029 | 38.25 | 4.10 | 3.22 | 0.00031 |
| LGALSL | 21.61 | 3.17 | 2.77 | 0.00029 | 4.96 | 0.53 | 3.22 | 0.00081 |
| RAB12 | 283.35 | 30.20 | 3.23 | 0.00029 | 249.74 | 27.00 | 3.21 | 0.00031 |
| RND3 | 173.06 | 29.65 | 2.54 | 0.00029 | 925.59 | 100.20 | 3.21 | 0.00031 |
| RAB2B | 97.56 | 8.45 | 3.53 | 0.00029 | 149.32 | 16.18 | 3.21 | 0.00031 |
| TGIF2 | 132.86 | 11.59 | 3.52 | 0.00029 | 137.71 | 15.02 | 3.20 | 0.00031 |
| GNAZ | 17.64 | 2.93 | 2.59 | 0.00029 | 3.13 | 0.34 | 3.20 | 0.00308 |
| KPNA4 | 90.15 | 13.78 | 2.71 | 0.00029 | 188.58 | 20.74 | 3.18 | 0.00240 |
| SKIL | 50.30 | 10.16 | 2.31 | 0.00029 | 118.12 | 13.00 | 3.18 | 0.00031 |
| MEIS1 | 6.52 | 0.89 | 2.87 | 0.00029 | 8.02 | 0.89 | 3.17 | 0.00057 |
| SKA1 | 115.28 | 15.08 | 2.93 | 0.00029 | 49.65 | 5.50 | 3.17 | 0.00031 |
| SDC4 | 1450.37 | 116.74 | 3.64 | 0.00029 | 883.67 | 98.26 | 3.17 | 0.00031 |
| PHKG1 | 69.37 | 12.84 | 2.43 | 0.00785 | 61.64 | 6.86 | 3.17 | 0.00341 |
| CAPZA2 | 231.35 | 49.32 | 2.23 | 0.00029 | 120.19 | 13.50 | 3.15 | 0.00031 |
| CCDC117 | 81.44 | 16.06 | 2.34 | 0.00029 | 39.75 | 4.48 | 3.15 | 0.00031 |
| NEIL2 | 15.70 | 2.17 | 2.86 | 0.00029 | 26.00 | 2.95 | 3.14 | 0.00031 |
| RBMS3 | 73.36 | 5.55 | 3.73 | 0.00029 | 124.62 | 14.17 | 3.14 | 0.00031 |
| PDCD4 | 105.11 | 20.98 | 2.32 | 0.00029 | 74.58 | 8.50 | 3.13 | 0.00031 |
| DTD2 | 29.09 | 4.48 | 2.70 | 0.00029 | 46.03 | 5.25 | 3.13 | 0.00031 |
| NUTF2 | 781.75 | 93.19 | 3.07 | 0.00029 | 413.32 | 47.15 | 3.13 | 0.00031 |
| MAK16 | 46.31 | 8.70 | 2.41 | 0.00029 | 138.32 | 15.82 | 3.13 | 0.00031 |
| TRA2A | 159.39 | 18.56 | 3.10 | 0.00029 | 134.15 | 15.43 | 3.12 | 0.00031 |
| DYRK1A | 306.53 | 32.79 | 3.22 | 0.00029 | 144.74 | 16.65 | 3.12 | 0.00031 |
| TMEM248 | 299.12 | 36.48 | 3.04 | 0.00029 | 97.91 | 11.29 | 3.12 | 0.00031 |
| NUDT19 | 16.73 | 2.30 | 2.87 | 0.00029 | 19.75 | 2.28 | 3.11 | 0.00031 |
| CHIC2 | 119.60 | 10.76 | 3.47 | 0.00029 | 159.38 | 18.49 | 3.11 | 0.00031 |
| FOXN3 | 164.08 | 19.27 | 3.09 | 0.00029 | 130.54 | 15.19 | 3.10 | 0.00031 |
| CBR4 | 19.95 | 3.48 | 2.52 | 0.00029 | 19.50 | 2.28 | 3.10 | 0.00031 |
| GINS1 | 464.90 | 39.69 | 3.55 | 0.00029 | 85.27 | 9.95 | 3.10 | 0.00031 |
| BAG4 | 49.78 | 8.45 | 2.56 | 0.00029 | 83.30 | 9.80 | 3.09 | 0.00031 |
| PRIM2 | 65.69 | 15.54 | 2.08 | 0.00029 | 60.79 | 7.17 | 3.08 | 0.00031 |
| TCEAL8 | 380.77 | 53.21 | 2.84 | 0.00029 | 341.11 | 40.34 | 3.08 | 0.00031 |
| SUMO1 | 756.95 | 105.22 | 2.85 | 0.00029 | 311.67 | 36.92 | 3.08 | 0.00031 |
| TMEM106B | 57.03 | 12.15 | 2.23 | 0.00029 | 55.75 | 6.62 | 3.07 | 0.00031 |
| EDA2R | 1.99 | 0.43 | 2.21 | 0.00621 | 11.00 | 1.31 | 3.07 | 0.00031 |
| MDM2 | 126.26 | 25.64 | 2.30 | 0.00029 | 97.44 | 11.63 | 3.07 | 0.00031 |
| ACVR2A | 11.74 | 2.04 | 2.52 | 0.03854 | 26.87 | 3.21 | 3.06 | 0.02844 |
| C18orf54 | 28.02 | 4.67 | 2.59 | 0.00029 | 13.63 | 1.63 | 3.06 | 0.00031 |
| ZNF747 | 30.72 | 5.73 | 2.42 | 0.00029 | 21.55 | 2.59 | 3.06 | 0.00031 |
| TMTC3 | 39.28 | 8.47 | 2.21 | 0.00029 | 54.58 | 6.57 | 3.05 | 0.00031 |
| DNMT3A | 89.97 | 9.22 | 3.29 | 0.00029 | 32.22 | 3.88 | 3.05 | 0.00031 |
| SLC16A7 | 26.71 | 4.67 | 2.52 | 0.00029 | 37.93 | 4.58 | 3.05 | 0.00031 |
| RAB8B | 73.40 | 17.08 | 2.10 | 0.00029 | 57.05 | 6.90 | 3.05 | 0.00031 |
| TRPC1 | 29.24 | 4.82 | 2.60 | 0.00029 | 18.78 | 2.27 | 3.05 | 0.00031 |
| SYNC | 240.05 | 36.71 | 2.71 | 0.00689 | 140.94 | 17.19 | 3.04 | 0.00103 |
| AGPAT9 | 15.92 | 2.10 | 2.92 | 0.00029 | 267.64 | 32.67 | 3.03 | 0.00031 |
| SEC22B | 515.01 | 67.43 | 2.93 | 0.00029 | 418.00 | 51.19 | 3.03 | 0.00031 |
| BCL10 | 55.81 | 8.04 | 2.79 | 0.00029 | 102.84 | 12.61 | 3.03 | 0.00031 |
| TMED7 | 268.60 | 40.89 | 2.72 | 0.00029 | 766.28 | 94.34 | 3.02 | 0.00031 |
| CENPH | 147.72 | 28.48 | 2.37 | 0.00029 | 54.35 | 6.72 | 3.02 | 0.00031 |
| ARRDC3 | 247.45 | 35.28 | 2.81 | 0.00029 | 54.66 | 6.79 | 3.01 | 0.00031 |
| ZNF587B | 12.45 | 2.39 | 2.38 | 0.00029 | 39.45 | 4.90 | 3.01 | 0.00031 |
| BROX | 257.77 | 40.96 | 2.65 | 0.00029 | 234.37 | 29.21 | 3.00 | 0.00031 |
| QKI | 135.46 | 17.31 | 2.97 | 0.00029 | 131.61 | 16.48 | 3.00 | 0.00031 |
| FAM221A | 7.38 | 1.22 | 2.60 | 0.00207 | 19.11 | 2.39 | 3.00 | 0.00031 |
| MZT1 | 102.12 | 23.31 | 2.13 | 0.00029 | 50.73 | 6.36 | 2.99 | 0.00031 |
| BBS10 | 26.42 | 3.85 | 2.78 | 0.00029 | 65.18 | 8.22 | 2.99 | 0.00031 |
| CPEB3 | 5.77 | 0.83 | 2.80 | 0.00029 | 3.28 | 0.41 | 2.99 | 0.00184 |
| PLD6 | 10.78 | 1.88 | 2.52 | 0.00029 | 11.73 | 1.49 | 2.98 | 0.00031 |
| JAK3 | 18.28 | 1.99 | 3.20 | 0.00029 | 5.25 | 0.67 | 2.98 | 0.00031 |
| SLC35F6 | 170.42 | 27.28 | 2.64 | 0.00029 | 89.75 | 11.42 | 2.97 | 0.00031 |
| SLMO2 | 375.80 | 72.62 | 2.37 | 0.00029 | 386.16 | 49.23 | 2.97 | 0.00031 |
| TMEM41B | 80.35 | 15.31 | 2.39 | 0.00029 | 135.57 | 17.29 | 2.97 | 0.00031 |
| SUDS3 | 94.13 | 12.26 | 2.94 | 0.00029 | 151.54 | 19.35 | 2.97 | 0.00031 |
| WTAP | 322.38 | 41.34 | 2.96 | 0.00029 | 307.44 | 39.28 | 2.97 | 0.00031 |
| CDKN2AIPNL | 148.01 | 25.42 | 2.54 | 0.00029 | 112.51 | 14.38 | 2.97 | 0.00031 |
| TWIST1 | 17.57 | 1.80 | 3.28 | 0.00029 | 47.43 | 6.09 | 2.96 | 0.00031 |
| TMEM159 | 49.20 | 6.88 | 2.84 | 0.00029 | 62.33 | 8.01 | 2.96 | 0.00031 |
| ZMPSTE24 | 160.83 | 25.46 | 2.66 | 0.00029 | 194.05 | 24.93 | 2.96 | 0.00031 |
| NUDT4P1 | 47.06 | 9.42 | 2.32 | 0.00029 | 126.68 | 16.31 | 2.96 | 0.00031 |
| TOR1AIP2 | 128.38 | 29.22 | 2.14 | 0.00029 | 152.54 | 19.69 | 2.95 | 0.00031 |
| TMCO1 | 141.79 | 17.61 | 3.01 | 0.00029 | 100.18 | 12.97 | 2.95 | 0.00031 |
| TROVE2 | 123.19 | 27.70 | 2.15 | 0.00029 | 130.13 | 17.00 | 2.94 | 0.00031 |
| GDPGP1 | 7.49 | 1.28 | 2.55 | 0.00029 | 12.88 | 1.68 | 2.93 | 0.00103 |
| FOXP1 | 60.10 | 7.84 | 2.94 | 0.00029 | 139.91 | 18.31 | 2.93 | 0.00031 |
| NHLRC2 | 61.37 | 13.49 | 2.19 | 0.00029 | 31.61 | 4.14 | 2.93 | 0.00031 |
| PELI2 | 1.13 | 0.22 | 2.35 | 0.00563 | 1.98 | 0.26 | 2.92 | 0.02544 |
| PRELID2 | 31.11 | 7.17 | 2.12 | 0.00029 | 23.70 | 3.13 | 2.92 | 0.00031 |
| ZNF273 | 11.87 | 2.62 | 2.18 | 0.00029 | 3.78 | 0.50 | 2.91 | 0.00221 |
| TMEM33 | 63.89 | 14.20 | 2.17 | 0.00029 | 188.22 | 25.07 | 2.91 | 0.00031 |
| FAM76B | 33.56 | 6.36 | 2.40 | 0.00029 | 33.70 | 4.50 | 2.90 | 0.00031 |
| SLC30A4 | 16.76 | 3.72 | 2.17 | 0.00029 | 8.85 | 1.19 | 2.90 | 0.00031 |
| RIT1 | 34.15 | 8.04 | 2.09 | 0.00029 | 33.03 | 4.44 | 2.90 | 0.00031 |
| S1PR2 | 24.45 | 4.17 | 2.55 | 0.00029 | 22.25 | 2.99 | 2.90 | 0.00031 |
| RNF219 | 100.94 | 21.83 | 2.21 | 0.00029 | 17.01 | 2.29 | 2.89 | 0.00031 |
| SLC25A32 | 50.48 | 8.68 | 2.54 | 0.00029 | 120.31 | 16.30 | 2.88 | 0.00057 |
| FAM204A | 82.14 | 19.81 | 2.05 | 0.00029 | 82.39 | 11.18 | 2.88 | 0.00031 |
| COQ7 | 89.61 | 15.54 | 2.53 | 0.00029 | 25.39 | 3.47 | 2.87 | 0.00031 |
| ACBD3 | 158.87 | 30.60 | 2.38 | 0.00029 | 188.03 | 25.68 | 2.87 | 0.00031 |
| MALL | 42.61 | 5.32 | 3.00 | 0.00029 | 43.65 | 5.97 | 2.87 | 0.00031 |
| DCUN1D3 | 72.41 | 5.58 | 3.70 | 0.00029 | 34.10 | 4.66 | 2.87 | 0.00031 |
| SRGAP2C | 67.74 | 13.18 | 2.36 | 0.00275 | 63.31 | 8.67 | 2.87 | 0.00031 |
| WHAMMP2 | 3.66 | 0.87 | 2.07 | 0.00075 | 1.90 | 0.26 | 2.87 | 0.00975 |
| EFNA5 | 24.23 | 3.60 | 2.75 | 0.00029 | 56.82 | 7.82 | 2.86 | 0.00031 |
| PPP6R3 | 238.31 | 46.86 | 2.35 | 0.00029 | 227.60 | 31.33 | 2.86 | 0.00031 |
| C12orf23 | 217.48 | 35.11 | 2.63 | 0.00029 | 218.62 | 30.11 | 2.86 | 0.00031 |
| ANKRD1 | 184.75 | 23.81 | 2.96 | 0.00029 | 23.31 | 3.21 | 2.86 | 0.00031 |
| UHRF1 | 293.24 | 41.67 | 2.82 | 0.00029 | 65.60 | 9.09 | 2.85 | 0.00031 |
| RALGPS2 | 65.02 | 14.64 | 2.15 | 0.00029 | 93.32 | 12.95 | 2.85 | 0.00031 |
| EIF4E | 331.80 | 22.67 | 3.87 | 0.00029 | 241.61 | 33.53 | 2.85 | 0.00031 |
| SFPQ | 614.46 | 100.02 | 2.62 | 0.00029 | 727.09 | 101.02 | 2.85 | 0.00031 |
| SMNDC1 | 133.64 | 15.51 | 3.11 | 0.00029 | 108.22 | 15.06 | 2.84 | 0.00031 |
| RELL1 | 185.20 | 28.23 | 2.71 | 0.00029 | 98.73 | 13.74 | 2.84 | 0.00031 |
| C5orf15 | 415.54 | 65.98 | 2.65 | 0.00029 | 240.90 | 33.56 | 2.84 | 0.00031 |
| HNRNPL | 1068.80 | 111.34 | 3.26 | 0.00029 | 902.08 | 125.70 | 2.84 | 0.00031 |
| KLF9 | 35.44 | 4.00 | 3.15 | 0.00029 | 48.05 | 6.72 | 2.84 | 0.00031 |
| PTBP3 | 147.48 | 28.15 | 2.39 | 0.00029 | 79.14 | 11.09 | 2.84 | 0.00031 |
| MIR100HG | 1.32 | 0.33 | 2.01 | 0.01481 | 77.12 | 10.82 | 2.83 | 0.00031 |
| CTNNB1 | 361.05 | 71.62 | 2.33 | 0.00029 | 496.89 | 69.78 | 2.83 | 0.00031 |
| ALKBH5 | 346.01 | 38.45 | 3.17 | 0.00029 | 220.94 | 31.04 | 2.83 | 0.00031 |
| SAR1B | 77.37 | 13.28 | 2.54 | 0.00029 | 48.14 | 6.77 | 2.83 | 0.00031 |
| CRKL | 300.41 | 35.34 | 3.09 | 0.00029 | 104.18 | 14.67 | 2.83 | 0.00031 |
| ARPP19 | 357.26 | 51.09 | 2.81 | 0.00029 | 215.44 | 30.35 | 2.83 | 0.00031 |
| PABPN1 | 65.45 | 5.05 | 3.70 | 0.02117 | 75.21 | 10.63 | 2.82 | 0.01310 |
| CDKAL1 | 43.50 | 10.19 | 2.09 | 0.00029 | 32.13 | 4.54 | 2.82 | 0.00031 |
| ANP32E | 210.65 | 43.58 | 2.27 | 0.00029 | 71.30 | 10.09 | 2.82 | 0.00031 |
| SPIN1 | 237.81 | 36.01 | 2.72 | 0.00029 | 136.93 | 19.37 | 2.82 | 0.00031 |
| PDE4D | 26.01 | 5.48 | 2.25 | 0.00029 | 25.07 | 3.55 | 2.82 | 0.00031 |
| RBBP4 | 183.30 | 29.88 | 2.62 | 0.00029 | 107.71 | 15.24 | 2.82 | 0.00031 |
| PITPNB | 78.36 | 11.71 | 2.74 | 0.00029 | 68.95 | 9.77 | 2.82 | 0.00031 |
| C6orf62 | 483.01 | 54.74 | 3.14 | 0.00029 | 452.97 | 64.21 | 2.82 | 0.00031 |
| SGMS2 | 50.89 | 11.42 | 2.16 | 0.00029 | 18.97 | 2.69 | 2.82 | 0.00031 |
| NFIA | 8.73 | 1.02 | 3.10 | 0.00029 | 18.68 | 2.65 | 2.82 | 0.00031 |
| CCNJ | 17.42 | 3.10 | 2.49 | 0.00415 | 20.30 | 2.88 | 2.82 | 0.00704 |
| ZFX | 75.33 | 15.44 | 2.29 | 0.00029 | 67.75 | 9.62 | 2.82 | 0.00031 |
| SPCS3 | 208.51 | 37.83 | 2.46 | 0.00029 | 202.38 | 28.76 | 2.81 | 0.00031 |
| SOX9 | 15.14 | 1.90 | 3.00 | 0.00029 | 185.58 | 26.39 | 2.81 | 0.00031 |
| C18orf25 | 39.95 | 6.41 | 2.64 | 0.00029 | 54.83 | 7.81 | 2.81 | 0.00031 |
| ATP1B1 | 203.34 | 22.66 | 3.17 | 0.00029 | 47.43 | 6.77 | 2.81 | 0.00031 |
| FUT8 | 76.03 | 12.24 | 2.64 | 0.00029 | 248.89 | 35.57 | 2.81 | 0.00031 |
| DDX6 | 208.42 | 39.19 | 2.41 | 0.00029 | 197.04 | 28.30 | 2.80 | 0.00031 |
| PAN3 | 54.37 | 8.16 | 2.74 | 0.00029 | 48.50 | 6.98 | 2.80 | 0.00031 |
| ZNF568 | 6.25 | 0.91 | 2.78 | 0.00029 | 19.85 | 2.86 | 2.80 | 0.00031 |
| PCBP2 | 1258.74 | 82.96 | 3.92 | 0.00029 | 1944.27 | 280.18 | 2.79 | 0.00145 |
| RUNX2 | 46.63 | 6.52 | 2.84 | 0.00029 | 123.17 | 17.76 | 2.79 | 0.00031 |
| EIF4G2 | 877.27 | 146.90 | 2.58 | 0.00029 | 2002.79 | 289.19 | 2.79 | 0.00031 |
| RBM15 | 64.87 | 14.73 | 2.14 | 0.00029 | 57.82 | 8.35 | 2.79 | 0.00031 |
| LCOR | 30.58 | 6.96 | 2.14 | 0.00029 | 21.51 | 3.11 | 2.79 | 0.00031 |
| CYP4V2 | 12.72 | 2.34 | 2.44 | 0.00029 | 39.50 | 5.72 | 2.79 | 0.00031 |
| TES | 302.16 | 50.20 | 2.59 | 0.00029 | 185.20 | 26.92 | 2.78 | 0.00031 |
| CHMP1B | 87.68 | 17.09 | 2.36 | 0.00029 | 91.52 | 13.32 | 2.78 | 0.00031 |
| CRADD | 12.55 | 3.07 | 2.03 | 0.00189 | 6.71 | 0.98 | 2.78 | 0.03988 |
| ZNF611 | 15.33 | 3.01 | 2.35 | 0.00029 | 19.82 | 2.89 | 2.78 | 0.00031 |
| KRBA2 | 11.37 | 2.65 | 2.10 | 0.00029 | 11.90 | 1.74 | 2.77 | 0.00057 |
| RC3H1 | 24.99 | 5.58 | 2.16 | 0.00029 | 28.35 | 4.15 | 2.77 | 0.00031 |
| CDC37L1 | 43.51 | 5.75 | 2.92 | 0.00029 | 49.56 | 7.27 | 2.77 | 0.00031 |
| OSBPL8 | 65.63 | 15.86 | 2.05 | 0.00029 | 138.78 | 20.47 | 2.76 | 0.00031 |
| MITF | 20.23 | 3.62 | 2.48 | 0.00029 | 15.40 | 2.28 | 2.76 | 0.00031 |
| CCDC126 | 14.12 | 2.63 | 2.43 | 0.00029 | 14.16 | 2.10 | 2.76 | 0.00031 |
| ALYREF | 1202.57 | 137.53 | 3.13 | 0.00029 | 387.88 | 57.57 | 2.75 | 0.00031 |
| POLR2D | 110.55 | 20.27 | 2.45 | 0.00029 | 91.82 | 13.65 | 2.75 | 0.00031 |
| ARF6 | 187.10 | 22.33 | 3.07 | 0.00029 | 239.48 | 35.68 | 2.75 | 0.00031 |
| PPP6C | 122.33 | 20.90 | 2.55 | 0.00029 | 75.50 | 11.27 | 2.74 | 0.00031 |
| IFI6 | 2516.04 | 303.33 | 3.05 | 0.00029 | 119.19 | 17.80 | 2.74 | 0.00031 |
| DUSP10 | 23.53 | 4.09 | 2.52 | 0.00029 | 189.27 | 28.28 | 2.74 | 0.00031 |
| HMGN2 | 2051.02 | 280.42 | 2.87 | 0.00029 | 914.32 | 136.72 | 2.74 | 0.00031 |
| LOC220729 | 24.39 | 5.99 | 2.03 | 0.00029 | 6.18 | 0.93 | 2.74 | 0.01132 |
| C2orf68 | 117.26 | 11.76 | 3.32 | 0.00029 | 92.45 | 13.88 | 2.74 | 0.00031 |
| CLDND1 | 193.03 | 30.13 | 2.68 | 0.00029 | 313.62 | 47.17 | 2.73 | 0.00031 |
| CSNK1A1 | 366.77 | 80.26 | 2.19 | 0.00029 | 811.80 | 122.36 | 2.73 | 0.00031 |
| FBXO48 | 3.58 | 0.87 | 2.04 | 0.00115 | 2.76 | 0.42 | 2.73 | 0.00812 |
| IMPA1 | 19.86 | 4.71 | 2.08 | 0.00029 | 31.70 | 4.78 | 2.73 | 0.00031 |
| C3orf52 | 10.40 | 2.16 | 2.27 | 0.00029 | 13.50 | 2.04 | 2.73 | 0.00031 |
| RAET1K | 1.02 | 0.20 | 2.33 | 0.04911 | 4.90 | 0.74 | 2.73 | 0.01091 |
| P4HA1 | 342.41 | 80.80 | 2.08 | 0.00029 | 92.10 | 13.94 | 2.72 | 0.00031 |
| BTF3L4 | 102.34 | 13.05 | 2.97 | 0.00029 | 172.24 | 26.12 | 2.72 | 0.00031 |
| TSGA10 | 7.24 | 1.63 | 2.15 | 0.00095 | 4.10 | 0.62 | 2.72 | 0.01846 |
| PTP4A1 | 70.93 | 9.91 | 2.84 | 0.00029 | 115.72 | 17.61 | 2.72 | 0.00031 |
| FBXO36 | 15.52 | 3.27 | 2.25 | 0.00029 | 24.28 | 3.70 | 2.71 | 0.00031 |
| MB21D2 | 27.62 | 4.44 | 2.64 | 0.00029 | 30.02 | 4.59 | 2.71 | 0.00031 |
| ZC2HC1A | 7.15 | 0.96 | 2.90 | 0.00029 | 14.33 | 2.19 | 2.71 | 0.00031 |
| OTUD1 | 12.47 | 1.63 | 2.94 | 0.00029 | 30.91 | 4.75 | 2.70 | 0.00031 |
| HSH2D | 3.24 | 0.74 | 2.14 | 0.00258 | 1.78 | 0.27 | 2.70 | 0.04449 |
| CANX | 958.27 | 232.82 | 2.04 | 0.01780 | 2332.50 | 359.08 | 2.70 | 0.01791 |
| PDIA4 | 646.87 | 111.40 | 2.54 | 0.00029 | 1208.88 | 186.14 | 2.70 | 0.00031 |
| ASIC2 | 2.63 | 0.34 | 2.95 | 0.00384 | 1.14 | 0.18 | 2.69 | 0.02717 |
| TMEM241 | 23.56 | 4.15 | 2.50 | 0.00029 | 32.21 | 5.00 | 2.69 | 0.00031 |
| EPS15 | 68.17 | 15.95 | 2.10 | 0.00029 | 189.70 | 29.62 | 2.68 | 0.00031 |
| ZBTB3 | 14.79 | 3.69 | 2.00 | 0.00029 | 15.98 | 2.50 | 2.68 | 0.00031 |
| DFFB | 11.86 | 1.99 | 2.58 | 0.00029 | 20.19 | 3.15 | 2.68 | 0.00031 |
| EIF2AK2 | 207.27 | 51.53 | 2.01 | 0.00029 | 94.54 | 14.84 | 2.67 | 0.00031 |
| TGFBR1 | 91.32 | 20.42 | 2.16 | 0.00029 | 41.65 | 6.55 | 2.67 | 0.00031 |
| TPM1 | 927.65 | 129.08 | 2.85 | 0.00029 | 610.74 | 96.74 | 2.66 | 0.00031 |
| SET | 1286.32 | 199.87 | 2.69 | 0.00029 | 944.59 | 149.68 | 2.66 | 0.00031 |
| FAM134C | 84.06 | 16.45 | 2.35 | 0.00029 | 105.40 | 16.72 | 2.66 | 0.00031 |
| AP1S2 | 71.66 | 17.78 | 2.01 | 0.00029 | 32.11 | 5.12 | 2.65 | 0.00031 |
| RRP15 | 24.55 | 5.81 | 2.08 | 0.00029 | 28.34 | 4.52 | 2.65 | 0.00031 |
| GTF2A1 | 68.14 | 14.40 | 2.24 | 0.00029 | 82.65 | 13.20 | 2.65 | 0.00031 |
| KPNB1 | 760.27 | 148.01 | 2.36 | 0.00029 | 1489.03 | 238.53 | 2.64 | 0.00031 |
| RNFT2 | 24.63 | 5.17 | 2.25 | 0.00029 | 20.57 | 3.30 | 2.64 | 0.00031 |
| FTO | 60.33 | 11.11 | 2.44 | 0.00029 | 36.64 | 5.90 | 2.63 | 0.00031 |
| MORN4 | 25.53 | 3.57 | 2.84 | 0.00029 | 30.70 | 4.95 | 2.63 | 0.00031 |
| OSR1 | 5.68 | 0.73 | 2.96 | 0.00029 | 3.75 | 0.61 | 2.63 | 0.01310 |
| ZNF808 | 15.13 | 3.02 | 2.33 | 0.00029 | 24.94 | 4.04 | 2.63 | 0.00031 |
| CADM1 | 54.55 | 9.07 | 2.59 | 0.00029 | 205.72 | 33.32 | 2.63 | 0.00031 |
| ATXN3 | 20.96 | 3.29 | 2.67 | 0.00029 | 39.65 | 6.42 | 2.63 | 0.00031 |
| DPYSL2 | 195.34 | 35.76 | 2.45 | 0.00029 | 57.83 | 9.38 | 2.62 | 0.00031 |
| CPEB2 | 5.32 | 0.83 | 2.68 | 0.00029 | 18.00 | 2.92 | 2.62 | 0.00031 |
| CIRBP | 199.27 | 32.99 | 2.59 | 0.00029 | 465.94 | 75.91 | 2.62 | 0.00031 |
| AEBP2 | 51.47 | 11.38 | 2.18 | 0.00029 | 37.66 | 6.14 | 2.62 | 0.00031 |
| ZNF770 | 60.44 | 14.55 | 2.05 | 0.00029 | 132.86 | 21.75 | 2.61 | 0.00031 |
| ACBD7 | 75.30 | 11.06 | 2.77 | 0.00029 | 14.31 | 2.34 | 2.61 | 0.00031 |
| SMU1 | 77.29 | 17.00 | 2.18 | 0.00029 | 49.10 | 8.06 | 2.61 | 0.00031 |
| ELAVL1 | 158.97 | 17.88 | 3.15 | 0.00029 | 93.25 | 15.34 | 2.60 | 0.00031 |
| EIF4E3 | 4.04 | 0.55 | 2.89 | 0.00029 | 5.41 | 0.89 | 2.60 | 0.00031 |
| UBE2E2 | 152.38 | 12.50 | 3.61 | 0.00029 | 89.54 | 14.77 | 2.60 | 0.00031 |
| INO80D | 16.15 | 3.67 | 2.14 | 0.00029 | 21.35 | 3.53 | 2.60 | 0.00031 |
| LOC100505876 | 56.84 | 12.15 | 2.23 | 0.00153 | 44.47 | 7.38 | 2.59 | 0.00124 |
| RMND5A | 102.30 | 18.11 | 2.50 | 0.00053 | 71.20 | 11.83 | 2.59 | 0.00031 |
| DCAKD | 198.23 | 19.77 | 3.33 | 0.00029 | 47.93 | 7.97 | 2.59 | 0.00031 |
| RQCD1 | 218.57 | 28.49 | 2.94 | 0.00029 | 97.23 | 16.18 | 2.59 | 0.00031 |
| SERPINB9 | 27.97 | 5.15 | 2.44 | 0.00029 | 5.52 | 0.92 | 2.58 | 0.00031 |
| MEAF6 | 93.32 | 9.27 | 3.33 | 0.00029 | 109.01 | 18.19 | 2.58 | 0.00031 |
| POLR2M | 137.79 | 20.68 | 2.74 | 0.00029 | 116.23 | 19.42 | 2.58 | 0.00031 |
| SPC24 | 693.03 | 59.97 | 3.53 | 0.00029 | 124.88 | 20.87 | 2.58 | 0.00031 |
| LSM12 | 238.86 | 33.35 | 2.84 | 0.00029 | 327.48 | 54.92 | 2.58 | 0.00031 |
| TCF4 | 14.58 | 2.49 | 2.55 | 0.00029 | 16.19 | 2.72 | 2.57 | 0.00031 |
| STK24 | 185.69 | 29.61 | 2.65 | 0.00029 | 155.45 | 26.18 | 2.57 | 0.00031 |
| POLR2J3 | 10.84 | 1.55 | 2.81 | 0.00029 | 6.81 | 1.15 | 2.57 | 0.00439 |
| VGLL3 | 22.74 | 4.07 | 2.48 | 0.00029 | 12.47 | 2.11 | 2.56 | 0.00031 |
| BICC1 | 110.92 | 21.08 | 2.40 | 0.00029 | 39.88 | 6.74 | 2.56 | 0.00031 |
| LBH | 18.94 | 4.38 | 2.11 | 0.00029 | 2.57 | 0.44 | 2.56 | 0.04180 |
| ETV6 | 27.00 | 4.55 | 2.57 | 0.00029 | 76.87 | 13.00 | 2.56 | 0.00031 |
| SLC16A1 | 138.39 | 29.98 | 2.21 | 0.00029 | 178.28 | 30.23 | 2.56 | 0.00031 |
| C16orf52 | 25.81 | 3.43 | 2.91 | 0.00029 | 19.87 | 3.38 | 2.56 | 0.00031 |
| STYX | 23.76 | 5.04 | 2.24 | 0.00029 | 56.76 | 9.71 | 2.55 | 0.00031 |
| KATNAL1 | 48.77 | 10.80 | 2.17 | 0.00029 | 13.57 | 2.32 | 2.55 | 0.00031 |
| ATP6V1A | 110.89 | 23.19 | 2.26 | 0.00029 | 110.05 | 18.91 | 2.54 | 0.00031 |
| CMTM6 | 119.41 | 22.24 | 2.42 | 0.00029 | 174.80 | 30.05 | 2.54 | 0.00031 |
| RNF168 | 53.92 | 13.14 | 2.04 | 0.00029 | 69.04 | 11.87 | 2.54 | 0.00031 |
| CEPT1 | 71.14 | 14.07 | 2.34 | 0.00095 | 44.73 | 7.71 | 2.54 | 0.00031 |
| PITHD1 | 165.48 | 23.30 | 2.83 | 0.00029 | 204.94 | 35.36 | 2.54 | 0.00031 |
| RNF182 | 3.01 | 0.44 | 2.79 | 0.00075 | 2.62 | 0.45 | 2.53 | 0.00911 |
| STRN3 | 57.04 | 13.85 | 2.04 | 0.00029 | 71.82 | 12.41 | 2.53 | 0.00031 |
| MTFMT | 29.45 | 6.46 | 2.19 | 0.00029 | 17.67 | 3.06 | 2.53 | 0.00031 |
| XXYLT1 | 40.10 | 8.65 | 2.21 | 0.00029 | 20.66 | 3.59 | 2.53 | 0.00031 |
| GLE1 | 155.83 | 25.55 | 2.61 | 0.00029 | 63.50 | 11.03 | 2.53 | 0.00031 |
| MBNL2 | 214.73 | 33.92 | 2.66 | 0.00029 | 68.75 | 11.95 | 2.52 | 0.00031 |
| SEC23A | 286.17 | 60.91 | 2.23 | 0.00029 | 265.19 | 46.20 | 2.52 | 0.00031 |
| MSI2 | 69.70 | 8.81 | 2.98 | 0.00029 | 79.27 | 13.83 | 2.52 | 0.00031 |
| ARL15 | 22.89 | 2.91 | 2.97 | 0.00029 | 15.15 | 2.66 | 2.51 | 0.00031 |
| OSTM1 | 51.67 | 12.08 | 2.10 | 0.00029 | 55.62 | 9.75 | 2.51 | 0.00031 |
| NDNL2 | 83.94 | 15.12 | 2.47 | 0.00029 | 60.60 | 10.64 | 2.51 | 0.00031 |
| PMAIP1 | 40.00 | 9.75 | 2.04 | 0.00029 | 278.88 | 49.01 | 2.51 | 0.00031 |
| UHMK1 | 133.63 | 26.66 | 2.33 | 0.00029 | 128.47 | 22.58 | 2.51 | 0.00031 |
| CAPZA1 | 562.88 | 92.79 | 2.60 | 0.00029 | 439.37 | 77.25 | 2.51 | 0.00031 |
| PDP1 | 103.30 | 25.50 | 2.02 | 0.00029 | 118.46 | 20.83 | 2.51 | 0.00031 |
| LOC441454 | 22.48 | 5.18 | 2.12 | 0.00029 | 9.53 | 1.68 | 2.51 | 0.00561 |
| SNX18 | 53.16 | 10.20 | 2.38 | 0.00029 | 45.05 | 7.93 | 2.51 | 0.00031 |
| GLTP | 113.48 | 18.03 | 2.65 | 0.00029 | 154.00 | 27.13 | 2.50 | 0.00031 |
| SRSF9 | 576.26 | 86.07 | 2.74 | 0.00029 | 535.33 | 94.60 | 2.50 | 0.00031 |
| MTPAP | 39.00 | 9.74 | 2.00 | 0.00029 | 18.78 | 3.32 | 2.50 | 0.00031 |
| SENP5 | 66.41 | 16.03 | 2.05 | 0.00029 | 55.84 | 9.88 | 2.50 | 0.00031 |
| ISCA1 | 107.17 | 24.56 | 2.13 | 0.00029 | 39.02 | 6.91 | 2.50 | 0.00031 |
| ZNF280B | 17.09 | 3.85 | 2.15 | 0.00029 | 3.50 | 0.62 | 2.50 | 0.00031 |
| PDE12 | 30.99 | 7.21 | 2.10 | 0.00029 | 51.83 | 9.20 | 2.49 | 0.00031 |
| SLC18A2 | 15.42 | 3.17 | 2.28 | 0.00029 | 23.12 | 4.11 | 2.49 | 0.00031 |
| ATP13A3 | 168.24 | 37.53 | 2.16 | 0.00029 | 247.82 | 44.00 | 2.49 | 0.00031 |
| CDH2 | 75.34 | 17.79 | 2.08 | 0.00029 | 109.07 | 19.37 | 2.49 | 0.00031 |
| HECA | 23.48 | 4.31 | 2.45 | 0.00029 | 38.52 | 6.84 | 2.49 | 0.00031 |
| TRMT10A | 18.34 | 3.66 | 2.32 | 0.00029 | 12.10 | 2.16 | 2.49 | 0.00031 |
| SPRED1 | 26.00 | 6.14 | 2.08 | 0.00029 | 67.81 | 12.11 | 2.49 | 0.00031 |
| SUMF2 | 231.41 | 40.64 | 2.51 | 0.00029 | 175.43 | 31.40 | 2.48 | 0.00031 |
| NKX3-1 | 9.53 | 1.42 | 2.74 | 0.00029 | 30.68 | 5.49 | 2.48 | 0.00031 |
| EDIL3 | 616.25 | 137.77 | 2.16 | 0.00029 | 61.41 | 11.01 | 2.48 | 0.00031 |
| KRAS | 72.04 | 15.82 | 2.19 | 0.00029 | 54.66 | 9.81 | 2.48 | 0.00031 |
| REEP3 | 144.46 | 32.91 | 2.13 | 0.00029 | 69.65 | 12.58 | 2.47 | 0.00031 |
| ZBTB18 | 44.81 | 5.73 | 2.97 | 0.00029 | 71.67 | 12.96 | 2.47 | 0.00031 |
| PTPRS | 86.03 | 20.85 | 2.04 | 0.00029 | 136.37 | 24.68 | 2.47 | 0.00031 |
| CD70 | 1120.76 | 144.11 | 2.96 | 0.00029 | 242.35 | 44.04 | 2.46 | 0.00031 |
| RSPRY1 | 54.02 | 11.26 | 2.26 | 0.00029 | 49.87 | 9.08 | 2.46 | 0.00031 |
| SLC25A24 | 156.79 | 36.84 | 2.09 | 0.00029 | 110.56 | 20.23 | 2.45 | 0.00031 |
| STX6 | 52.18 | 9.71 | 2.43 | 0.00029 | 47.13 | 8.66 | 2.44 | 0.00031 |
| RNF2 | 68.50 | 13.26 | 2.37 | 0.00029 | 65.36 | 12.04 | 2.44 | 0.00031 |
| GSR | 217.14 | 40.30 | 2.43 | 0.00029 | 157.81 | 29.15 | 2.44 | 0.00031 |
| GABPB1 | 60.46 | 13.78 | 2.13 | 0.00029 | 44.13 | 8.16 | 2.43 | 0.00031 |
| EIF5A2 | 21.78 | 4.41 | 2.30 | 0.00029 | 23.40 | 4.35 | 2.43 | 0.00031 |
| MT1X | 226.63 | 26.44 | 3.10 | 0.00029 | 171.26 | 31.87 | 2.43 | 0.00031 |
| CNOT7 | 201.70 | 44.62 | 2.18 | 0.00029 | 125.22 | 23.45 | 2.42 | 0.00031 |
| TFAP2C | 17.40 | 3.91 | 2.15 | 0.00029 | 3.72 | 0.70 | 2.41 | 0.00827 |
| PMP22 | 159.42 | 24.97 | 2.67 | 0.00029 | 86.41 | 16.24 | 2.41 | 0.00031 |
| CSTF2T | 88.00 | 18.04 | 2.29 | 0.00029 | 32.96 | 6.20 | 2.41 | 0.00031 |
| ZNF652 | 35.67 | 8.07 | 2.14 | 0.00029 | 13.79 | 2.60 | 2.41 | 0.00031 |
| DLG3 | 37.12 | 8.13 | 2.19 | 0.00029 | 24.09 | 4.55 | 2.40 | 0.00031 |
| ZNF362 | 86.67 | 10.01 | 3.11 | 0.00029 | 58.87 | 11.16 | 2.40 | 0.00031 |
| TMEM65 | 57.92 | 13.02 | 2.15 | 0.00029 | 20.20 | 3.83 | 2.40 | 0.00031 |
| STX7 | 48.71 | 9.78 | 2.32 | 0.00029 | 49.95 | 9.47 | 2.40 | 0.00031 |
| NT5DC3 | 11.25 | 2.32 | 2.28 | 0.00029 | 25.14 | 4.77 | 2.40 | 0.00031 |
| NONO | 971.01 | 180.18 | 2.43 | 0.00053 | 998.70 | 189.59 | 2.40 | 0.00031 |
| TAF13 | 128.44 | 19.20 | 2.74 | 0.00029 | 239.70 | 45.60 | 2.39 | 0.00031 |
| SOX4 | 19.60 | 2.69 | 2.86 | 0.00029 | 58.50 | 11.14 | 2.39 | 0.00031 |
| TPM3 | 727.75 | 166.28 | 2.13 | 0.00029 | 389.19 | 74.14 | 2.39 | 0.00031 |
| LOH12CR1 | 9.88 | 2.14 | 2.21 | 0.00053 | 8.07 | 1.54 | 2.39 | 0.00291 |
| LOC284023 | 9.75 | 1.73 | 2.49 | 0.00029 | 8.47 | 1.61 | 2.39 | 0.00308 |
| SETD8 | 132.76 | 22.24 | 2.58 | 0.00029 | 158.09 | 30.16 | 2.39 | 0.00031 |
| MPLKIP | 91.02 | 20.79 | 2.13 | 0.00029 | 61.53 | 11.77 | 2.39 | 0.00031 |
| SERTAD3 | 85.26 | 11.79 | 2.85 | 0.00029 | 56.64 | 10.84 | 2.38 | 0.00031 |
| RBPJ | 136.78 | 31.76 | 2.11 | 0.00029 | 91.79 | 17.58 | 2.38 | 0.00031 |
| SKA2 | 365.58 | 87.79 | 2.06 | 0.00029 | 107.67 | 20.62 | 2.38 | 0.00031 |
| CTTN | 364.42 | 82.90 | 2.14 | 0.00029 | 501.03 | 96.06 | 2.38 | 0.00031 |
| THAP10 | 20.01 | 2.87 | 2.80 | 0.00029 | 9.73 | 1.87 | 2.38 | 0.00124 |
| PAPOLG | 13.41 | 2.97 | 2.17 | 0.00029 | 11.71 | 2.27 | 2.37 | 0.00031 |
| MAL2 | 32.29 | 4.86 | 2.73 | 0.00029 | 41.18 | 7.98 | 2.37 | 0.00031 |
| PTP4A2 | 447.48 | 83.50 | 2.42 | 0.00029 | 506.50 | 98.17 | 2.37 | 0.00031 |
| EXOSC6 | 72.84 | 10.80 | 2.75 | 0.00029 | 136.39 | 26.53 | 2.36 | 0.00031 |
| PCNP | 223.00 | 55.62 | 2.00 | 0.00029 | 194.25 | 37.80 | 2.36 | 0.00031 |
| CNEP1R1 | 37.57 | 4.84 | 2.96 | 0.00029 | 28.30 | 5.53 | 2.36 | 0.00031 |
| TIFA | 26.05 | 3.42 | 2.93 | 0.00029 | 24.70 | 4.83 | 2.36 | 0.00031 |
| C17orf76-AS1 | 1208.24 | 230.10 | 2.39 | 0.00029 | 2008.47 | 393.29 | 2.35 | 0.00031 |
| LOC729013 | 46.12 | 6.49 | 2.83 | 0.00029 | 9.60 | 1.88 | 2.35 | 0.00785 |
| SRPK1 | 100.02 | 23.02 | 2.12 | 0.00029 | 80.52 | 15.78 | 2.35 | 0.00031 |
| TRPS1 | 0.92 | 0.18 | 2.33 | 0.00662 | 19.87 | 3.90 | 2.35 | 0.00031 |
| ZHX2 | 9.54 | 1.81 | 2.40 | 0.00029 | 38.20 | 7.50 | 2.35 | 0.00031 |
| PGGT1B | 32.45 | 7.99 | 2.02 | 0.00029 | 39.18 | 7.70 | 2.35 | 0.00031 |
| PTCHD3P1 | 41.90 | 7.62 | 2.46 | 0.00029 | 12.33 | 2.43 | 2.35 | 0.00031 |
| GPR160 | 10.80 | 1.92 | 2.49 | 0.00029 | 4.23 | 0.83 | 2.34 | 0.02321 |
| FXN | 11.07 | 1.54 | 2.84 | 0.00029 | 6.74 | 1.33 | 2.34 | 0.00031 |
| TGIF1 | 120.93 | 18.96 | 2.67 | 0.00029 | 415.96 | 82.12 | 2.34 | 0.00031 |
| TBP | 30.47 | 5.90 | 2.37 | 0.00029 | 41.79 | 8.27 | 2.34 | 0.00031 |
| CELF1 | 143.62 | 24.37 | 2.56 | 0.00029 | 191.18 | 37.88 | 2.34 | 0.00031 |
| CLDN11 | 54.59 | 10.74 | 2.35 | 0.00029 | 25.85 | 5.14 | 2.33 | 0.00031 |
| ZDHHC2 | 54.88 | 10.90 | 2.33 | 0.00029 | 58.66 | 11.67 | 2.33 | 0.00031 |
| DAAM1 | 18.83 | 4.49 | 2.07 | 0.00029 | 25.99 | 5.17 | 2.33 | 0.00031 |
| RAB8A | 300.08 | 46.63 | 2.69 | 0.00029 | 140.12 | 27.91 | 2.33 | 0.00031 |
| PPP1R3B | 11.66 | 2.90 | 2.01 | 0.00029 | 22.71 | 4.53 | 2.33 | 0.00031 |
| HAUS2 | 104.44 | 15.95 | 2.71 | 0.00029 | 50.66 | 10.12 | 2.32 | 0.00031 |
| PRRG1 | 9.59 | 1.99 | 2.27 | 0.00029 | 16.01 | 3.20 | 2.32 | 0.00031 |
| SS18 | 152.04 | 33.21 | 2.19 | 0.00029 | 105.24 | 21.02 | 2.32 | 0.00031 |
| ARCN1 | 151.73 | 37.78 | 2.01 | 0.00029 | 318.17 | 63.60 | 2.32 | 0.00031 |
| ZEB2 | 17.84 | 4.46 | 2.00 | 0.00029 | 35.53 | 7.11 | 2.32 | 0.00031 |
| TMEM167A | 147.72 | 25.15 | 2.55 | 0.00029 | 73.59 | 14.83 | 2.31 | 0.00031 |
| PSMD10 | 174.25 | 30.23 | 2.53 | 0.00029 | 96.29 | 19.40 | 2.31 | 0.00031 |
| ZNF28 | 25.49 | 4.24 | 2.59 | 0.00029 | 33.42 | 6.74 | 2.31 | 0.00031 |
| LOC100127983 | 63.75 | 7.31 | 3.12 | 0.00029 | 94.81 | 19.14 | 2.31 | 0.00031 |
| CNN3 | 966.88 | 145.28 | 2.73 | 0.00029 | 923.08 | 186.43 | 2.31 | 0.00031 |
| AMFR | 111.56 | 21.90 | 2.35 | 0.00029 | 71.31 | 14.41 | 2.31 | 0.00031 |
| KLF11 | 62.01 | 11.59 | 2.42 | 0.00029 | 11.44 | 2.32 | 2.30 | 0.00031 |
| STARD7 | 460.83 | 83.92 | 2.46 | 0.00029 | 183.92 | 37.31 | 2.30 | 0.00031 |
| ASB16-AS1 | 22.13 | 4.77 | 2.21 | 0.00134 | 8.64 | 1.75 | 2.30 | 0.01976 |
| LARP1 | 238.59 | 45.99 | 2.38 | 0.00029 | 360.24 | 73.17 | 2.30 | 0.00031 |
| CDCA4 | 92.28 | 18.26 | 2.34 | 0.00029 | 77.29 | 15.71 | 2.30 | 0.00031 |
| TMEM189 | 269.76 | 37.84 | 2.83 | 0.00053 | 182.39 | 37.09 | 2.30 | 0.00422 |
| AMMECR1L | 34.18 | 6.02 | 2.50 | 0.00029 | 38.08 | 7.74 | 2.30 | 0.00031 |
| NEK7 | 98.21 | 20.95 | 2.23 | 0.00029 | 112.40 | 22.89 | 2.30 | 0.00031 |
| RCAN3 | 32.25 | 6.86 | 2.23 | 0.00029 | 26.97 | 5.50 | 2.29 | 0.00031 |
| RWDD2B | 74.82 | 18.59 | 2.01 | 0.00029 | 44.89 | 9.18 | 2.29 | 0.00031 |
| UBE2W | 23.57 | 3.71 | 2.67 | 0.00029 | 40.21 | 8.23 | 2.29 | 0.00184 |
| TLK1 | 75.89 | 11.11 | 2.77 | 0.00029 | 87.46 | 17.91 | 2.29 | 0.00031 |
| NHLRC3 | 49.13 | 8.56 | 2.52 | 0.00029 | 7.09 | 1.45 | 2.29 | 0.00081 |
| ERH | 1541.85 | 159.03 | 3.28 | 0.00029 | 1627.04 | 333.40 | 2.29 | 0.00031 |
| ARPC4 | 287.29 | 46.24 | 2.64 | 0.00029 | 330.48 | 67.79 | 2.29 | 0.00031 |
| SLC30A9 | 128.44 | 28.21 | 2.19 | 0.00029 | 111.16 | 22.83 | 2.28 | 0.00031 |
| SLC38A2 | 230.59 | 56.33 | 2.03 | 0.00029 | 626.49 | 128.79 | 2.28 | 0.00031 |
| TOMM22 | 408.43 | 63.94 | 2.68 | 0.00029 | 267.16 | 54.95 | 2.28 | 0.00031 |
| CPNE8 | 16.16 | 3.08 | 2.39 | 0.00029 | 58.38 | 12.02 | 2.28 | 0.00031 |
| SUMO2 | 2286.35 | 391.74 | 2.55 | 0.00029 | 1037.82 | 213.86 | 2.28 | 0.00031 |
| CSTF3 | 118.42 | 25.70 | 2.20 | 0.00029 | 60.74 | 12.52 | 2.28 | 0.00081 |
| MMGT1 | 61.65 | 12.75 | 2.27 | 0.00029 | 65.75 | 13.59 | 2.27 | 0.00031 |
| PRKAR2B | 17.99 | 3.77 | 2.25 | 0.00029 | 13.47 | 2.79 | 2.27 | 0.00031 |
| LYRM9 | 10.87 | 1.27 | 3.10 | 0.00115 | 4.31 | 0.89 | 2.27 | 0.02373 |
| GALNT4 | 4.24 | 1.01 | 2.07 | 0.03237 | 17.55 | 3.64 | 2.27 | 0.00031 |
| GATAD2B | 38.52 | 7.87 | 2.29 | 0.00029 | 28.76 | 5.96 | 2.27 | 0.00031 |
| TRAF3IP1 | 34.62 | 7.28 | 2.25 | 0.00029 | 22.08 | 4.58 | 2.27 | 0.00031 |
| PIAS2 | 29.40 | 7.28 | 2.01 | 0.00029 | 28.25 | 5.87 | 2.27 | 0.00031 |
| SOCS3 | 116.38 | 14.61 | 2.99 | 0.00029 | 67.84 | 14.11 | 2.27 | 0.00031 |
| LDLRAD3 | 12.91 | 2.48 | 2.38 | 0.00029 | 38.81 | 8.10 | 2.26 | 0.00031 |
| YWHAZ | 801.23 | 151.63 | 2.40 | 0.00029 | 925.68 | 193.79 | 2.26 | 0.00031 |
| HMG20A | 71.12 | 13.48 | 2.40 | 0.00029 | 36.85 | 7.74 | 2.25 | 0.00031 |
| DCBLD2 | 470.54 | 96.28 | 2.29 | 0.00029 | 1310.52 | 275.37 | 2.25 | 0.00341 |
| DCAF16 | 106.26 | 18.44 | 2.53 | 0.00029 | 82.42 | 17.38 | 2.25 | 0.00031 |
| FKBP5 | 57.16 | 12.04 | 2.25 | 0.00029 | 10.85 | 2.30 | 2.24 | 0.00031 |
| MEX3D | 75.49 | 11.00 | 2.78 | 0.00029 | 42.51 | 9.03 | 2.24 | 0.00031 |
| RARA | 66.80 | 10.12 | 2.72 | 0.00029 | 76.42 | 16.23 | 2.24 | 0.00031 |
| TP53INP1 | 13.97 | 3.03 | 2.20 | 0.00029 | 7.10 | 1.51 | 2.23 | 0.00031 |
| MBNL1 | 184.28 | 33.62 | 2.45 | 0.00029 | 183.81 | 39.25 | 2.23 | 0.00031 |
| TM9SF3 | 243.85 | 58.13 | 2.07 | 0.00029 | 228.24 | 48.79 | 2.23 | 0.00031 |
| USF1 | 132.89 | 23.71 | 2.49 | 0.00029 | 134.18 | 28.68 | 2.23 | 0.00031 |
| ORC2 | 52.56 | 11.74 | 2.16 | 0.00029 | 36.48 | 7.82 | 2.22 | 0.00031 |
| CTBP1 | 316.61 | 55.76 | 2.51 | 0.00029 | 191.08 | 41.35 | 2.21 | 0.00031 |
| PPP3R1 | 151.01 | 17.48 | 3.11 | 0.00029 | 146.73 | 31.76 | 2.21 | 0.00031 |
| CASP8 | 31.40 | 7.40 | 2.09 | 0.00029 | 43.11 | 9.34 | 2.21 | 0.00031 |
| RAP2C | 84.09 | 13.05 | 2.69 | 0.00029 | 120.35 | 26.12 | 2.20 | 0.00031 |
| UGCG | 412.31 | 94.16 | 2.13 | 0.00029 | 280.50 | 60.93 | 2.20 | 0.00031 |
| RNF144B | 48.75 | 11.54 | 2.08 | 0.00029 | 3.67 | 0.80 | 2.20 | 0.00221 |
| MXD1 | 5.40 | 1.14 | 2.25 | 0.00029 | 14.04 | 3.05 | 2.20 | 0.00031 |
| MORF4L1 | 582.46 | 138.80 | 2.07 | 0.00029 | 351.65 | 76.48 | 2.20 | 0.00031 |
| UBE2D3 | 565.68 | 134.00 | 2.08 | 0.00029 | 483.26 | 105.10 | 2.20 | 0.00031 |
| THOC6 | 145.69 | 28.11 | 2.37 | 0.02721 | 51.83 | 11.28 | 2.20 | 0.00453 |
| PTMA | 6206.69 | 1011.20 | 2.62 | 0.00743 | 2528.35 | 550.51 | 2.20 | 0.00031 |
| PLOD2 | 562.35 | 136.01 | 2.05 | 0.00029 | 175.69 | 38.27 | 2.20 | 0.00031 |
| RHOB | 150.80 | 22.34 | 2.75 | 0.00029 | 72.78 | 15.91 | 2.19 | 0.00031 |
| C1orf174 | 135.39 | 18.97 | 2.84 | 0.00029 | 118.31 | 25.87 | 2.19 | 0.00031 |
| PDZD8 | 106.42 | 21.11 | 2.33 | 0.00029 | 104.21 | 22.82 | 2.19 | 0.00031 |
| ARL6IP1 | 1024.53 | 217.79 | 2.23 | 0.00029 | 169.63 | 37.15 | 2.19 | 0.00031 |
| YWHAG | 408.23 | 78.99 | 2.37 | 0.00029 | 176.92 | 38.78 | 2.19 | 0.00031 |
| YWHAE | 1569.34 | 227.49 | 2.79 | 0.00029 | 1167.68 | 256.56 | 2.19 | 0.00031 |
| CCNY | 115.64 | 23.38 | 2.31 | 0.00029 | 63.48 | 13.95 | 2.19 | 0.00031 |
| TNFRSF1A | 316.31 | 61.82 | 2.36 | 0.00029 | 142.39 | 31.38 | 2.18 | 0.00031 |
| SNRNP27 | 169.10 | 33.88 | 2.32 | 0.00029 | 74.78 | 16.50 | 2.18 | 0.00031 |
| VHL | 50.83 | 9.32 | 2.45 | 0.00029 | 89.38 | 19.74 | 2.18 | 0.00031 |
| CDV3 | 349.94 | 64.76 | 2.43 | 0.00029 | 290.95 | 64.29 | 2.18 | 0.00031 |
| IRF2 | 49.87 | 8.85 | 2.49 | 0.00029 | 101.71 | 22.48 | 2.18 | 0.00031 |
| EGLN1 | 65.92 | 13.96 | 2.24 | 0.00029 | 47.81 | 10.57 | 2.18 | 0.00031 |
| GATAD2A | 105.35 | 20.20 | 2.38 | 0.00029 | 89.77 | 19.87 | 2.18 | 0.00031 |
| LSM14A | 85.55 | 15.20 | 2.49 | 0.00029 | 142.50 | 31.55 | 2.18 | 0.00031 |
| C5orf22 | 93.37 | 14.40 | 2.70 | 0.00029 | 71.40 | 15.85 | 2.17 | 0.00031 |
| PTK7 | 151.10 | 35.35 | 2.10 | 0.00029 | 49.89 | 11.11 | 2.17 | 0.00031 |
| SDAD1 | 62.75 | 14.21 | 2.14 | 0.00029 | 186.83 | 41.61 | 2.17 | 0.00031 |
| ZNF252P | 32.58 | 7.27 | 2.16 | 0.00029 | 42.84 | 9.55 | 2.17 | 0.00031 |
| MTMR4 | 70.02 | 16.81 | 2.06 | 0.00029 | 170.82 | 38.15 | 2.16 | 0.00031 |
| PELI1 | 25.16 | 5.06 | 2.31 | 0.00029 | 13.63 | 3.04 | 2.16 | 0.00031 |
| RIMKLB | 18.99 | 4.60 | 2.05 | 0.00029 | 13.23 | 2.96 | 2.16 | 0.00031 |
| CA13 | 18.40 | 4.27 | 2.11 | 0.00029 | 4.38 | 0.98 | 2.16 | 0.00203 |
| XPR1 | 25.46 | 6.23 | 2.03 | 0.00029 | 26.06 | 5.85 | 2.16 | 0.00031 |
| EBP | 402.50 | 69.06 | 2.54 | 0.00029 | 111.51 | 25.04 | 2.16 | 0.00031 |
| IER3IP1 | 158.85 | 33.65 | 2.24 | 0.00029 | 89.85 | 20.20 | 2.15 | 0.00031 |
| NADKD1 | 46.98 | 11.42 | 2.04 | 0.00029 | 44.60 | 10.03 | 2.15 | 0.00031 |
| TMED4 | 349.40 | 65.82 | 2.41 | 0.00029 | 160.67 | 36.21 | 2.15 | 0.00031 |
| HDGFRP3 | 153.00 | 31.61 | 2.27 | 0.00029 | 68.11 | 15.37 | 2.15 | 0.00031 |
| MTA2 | 412.76 | 72.77 | 2.50 | 0.00029 | 349.21 | 78.90 | 2.15 | 0.00031 |
| UPK3BL | 9.92 | 1.88 | 2.40 | 0.00321 | 11.57 | 2.61 | 2.15 | 0.02577 |
| CPEB1 | 10.55 | 2.08 | 2.34 | 0.00053 | 8.08 | 1.82 | 2.15 | 0.00031 |
| PLCB3 | 100.44 | 20.78 | 2.27 | 0.00029 | 72.52 | 16.41 | 2.14 | 0.00031 |
| SEMA4D | 35.87 | 8.89 | 2.01 | 0.00029 | 15.93 | 3.61 | 2.14 | 0.00031 |
| TSC22D2 | 62.48 | 10.65 | 2.55 | 0.00029 | 66.05 | 15.00 | 2.14 | 0.00031 |
| GLIPR2 | 88.31 | 14.68 | 2.59 | 0.00029 | 15.13 | 3.44 | 2.14 | 0.00031 |
| GSTCD | 31.48 | 7.85 | 2.00 | 0.00029 | 22.53 | 5.13 | 2.14 | 0.00031 |
| ARHGEF11 | 66.55 | 11.10 | 2.58 | 0.00029 | 50.03 | 11.40 | 2.13 | 0.00031 |
| TAF12 | 118.91 | 24.83 | 2.26 | 0.00029 | 156.97 | 35.80 | 2.13 | 0.00031 |
| ATF2 | 129.68 | 25.68 | 2.34 | 0.00029 | 92.10 | 21.03 | 2.13 | 0.00031 |
| RPP14 | 29.46 | 7.02 | 2.07 | 0.00029 | 28.40 | 6.49 | 2.13 | 0.00031 |
| SEH1L | 123.25 | 18.40 | 2.74 | 0.00029 | 162.20 | 37.06 | 2.13 | 0.00031 |
| EIF1AX | 123.04 | 29.41 | 2.06 | 0.00029 | 72.89 | 16.73 | 2.12 | 0.00031 |
| KCNJ2 | 3.30 | 0.59 | 2.47 | 0.00029 | 5.43 | 1.25 | 2.12 | 0.00031 |
| IGFBPL1 | 34.32 | 5.42 | 2.66 | 0.00029 | 4.65 | 1.07 | 2.12 | 0.00704 |
| SLC39A11 | 23.90 | 5.85 | 2.03 | 0.00029 | 12.80 | 2.96 | 2.11 | 0.00031 |
| HOOK3 | 22.13 | 5.20 | 2.09 | 0.00029 | 30.77 | 7.13 | 2.11 | 0.00031 |
| GCA | 16.02 | 3.66 | 2.13 | 0.00029 | 10.56 | 2.45 | 2.11 | 0.00031 |
| MOB1A | 677.36 | 133.29 | 2.35 | 0.00029 | 296.85 | 68.74 | 2.11 | 0.00031 |
| CDON | 5.84 | 1.34 | 2.13 | 0.00029 | 8.16 | 1.89 | 2.11 | 0.00031 |
| FUT2 | 2.51 | 0.63 | 2.00 | 0.00430 | 1.99 | 0.46 | 2.11 | 0.03500 |
| TRAPPC6B | 29.08 | 6.25 | 2.22 | 0.00029 | 43.34 | 10.06 | 2.11 | 0.00031 |
| INO80C | 12.58 | 1.82 | 2.79 | 0.00029 | 17.23 | 4.01 | 2.10 | 0.00840 |
| FBXO46 | 21.32 | 4.78 | 2.16 | 0.00029 | 22.00 | 5.12 | 2.10 | 0.00031 |
| HSPA1B | 136.35 | 22.87 | 2.58 | 0.00029 | 210.86 | 49.31 | 2.10 | 0.00031 |
| VSIG10 | 28.18 | 6.12 | 2.20 | 0.00029 | 28.28 | 6.62 | 2.09 | 0.00031 |
| FCF1 | 76.83 | 11.48 | 2.74 | 0.00029 | 100.26 | 23.52 | 2.09 | 0.00031 |
| YAP1 | 158.70 | 32.25 | 2.30 | 0.00029 | 158.44 | 37.18 | 2.09 | 0.00031 |
| CDK2AP1 | 475.47 | 79.87 | 2.57 | 0.00029 | 269.45 | 63.29 | 2.09 | 0.00031 |
| YIPF6 | 44.80 | 11.02 | 2.02 | 0.00029 | 38.43 | 9.03 | 2.09 | 0.00031 |
| DNAJB9 | 15.98 | 3.95 | 2.02 | 0.00029 | 52.85 | 12.43 | 2.09 | 0.00031 |
| KCTD6 | 19.13 | 4.14 | 2.21 | 0.00029 | 10.35 | 2.44 | 2.09 | 0.00633 |
| PPP3CA | 87.05 | 19.23 | 2.18 | 0.00029 | 76.94 | 18.14 | 2.08 | 0.00031 |
| SLC25A42 | 14.28 | 2.29 | 2.64 | 0.00029 | 9.74 | 2.30 | 2.08 | 0.00031 |
| STARD3NL | 101.76 | 19.35 | 2.39 | 0.00029 | 45.97 | 10.90 | 2.08 | 0.00031 |
| TM6SF1 | 35.17 | 6.70 | 2.39 | 0.00029 | 18.43 | 4.37 | 2.08 | 0.00031 |
| TMEM97 | 279.28 | 41.21 | 2.76 | 0.00029 | 53.30 | 12.67 | 2.07 | 0.00057 |
| TMEM9B | 82.87 | 14.73 | 2.49 | 0.00029 | 85.79 | 20.42 | 2.07 | 0.00031 |
| PACRGL | 41.04 | 9.28 | 2.14 | 0.00029 | 28.77 | 6.85 | 2.07 | 0.00031 |
| MARK2 | 66.98 | 11.82 | 2.50 | 0.00029 | 64.93 | 15.52 | 2.06 | 0.00031 |
| CD44 | 70.99 | 11.24 | 2.66 | 0.00029 | 1263.20 | 302.08 | 2.06 | 0.00184 |
| RABL2B | 9.34 | 2.00 | 2.23 | 0.00029 | 5.39 | 1.29 | 2.06 | 0.01602 |
| TCF7L2 | 27.47 | 4.20 | 2.71 | 0.00029 | 23.58 | 5.66 | 2.06 | 0.00031 |
| KLF3 | 26.36 | 6.00 | 2.13 | 0.00029 | 23.14 | 5.56 | 2.06 | 0.00031 |
| CYB5B | 139.52 | 29.15 | 2.26 | 0.00029 | 38.81 | 9.34 | 2.06 | 0.00031 |
| CNP | 124.91 | 23.89 | 2.39 | 0.00029 | 61.62 | 14.84 | 2.05 | 0.01566 |
| HEXIM1 | 34.57 | 8.12 | 2.09 | 0.00029 | 42.15 | 10.16 | 2.05 | 0.00031 |
| MKS1 | 40.70 | 6.75 | 2.59 | 0.00029 | 26.08 | 6.30 | 2.05 | 0.00031 |
| GMEB1 | 18.13 | 3.76 | 2.27 | 0.00029 | 38.03 | 9.23 | 2.04 | 0.00031 |
| DUSP19 | 10.36 | 1.71 | 2.60 | 0.00029 | 2.13 | 0.52 | 2.04 | 0.00949 |
| SLC50A1 | 165.36 | 33.71 | 2.29 | 0.00029 | 143.77 | 34.91 | 2.04 | 0.00031 |
| DR1 | 98.67 | 19.24 | 2.36 | 0.00029 | 134.72 | 32.72 | 2.04 | 0.00031 |
| GREB1 | 1.07 | 0.26 | 2.03 | 0.00207 | 2.47 | 0.60 | 2.04 | 0.00103 |
| CDC42SE1 | 116.86 | 21.26 | 2.46 | 0.00029 | 106.83 | 26.01 | 2.04 | 0.00031 |
| EVA1A | 20.70 | 4.67 | 2.15 | 0.00029 | 54.56 | 13.29 | 2.04 | 0.00031 |
| SNRNP40 | 366.64 | 51.40 | 2.83 | 0.00029 | 320.65 | 78.12 | 2.04 | 0.00031 |
| RAP2A | 85.43 | 20.13 | 2.09 | 0.00029 | 45.39 | 11.08 | 2.03 | 0.00031 |
| EHD2 | 163.00 | 23.67 | 2.78 | 0.00029 | 110.94 | 27.09 | 2.03 | 0.00031 |
| NET1 | 157.03 | 33.60 | 2.22 | 0.00029 | 53.62 | 13.11 | 2.03 | 0.00031 |
| SLC2A3 | 83.47 | 19.88 | 2.07 | 0.00029 | 40.39 | 9.89 | 2.03 | 0.00031 |
| SRPRB | 85.31 | 20.56 | 2.05 | 0.00029 | 73.31 | 17.97 | 2.03 | 0.00031 |
| PRKAG2 | 61.22 | 11.88 | 2.37 | 0.00029 | 19.71 | 4.83 | 2.03 | 0.00031 |
| RIN2 | 68.99 | 15.93 | 2.12 | 0.00029 | 83.07 | 20.38 | 2.03 | 0.00031 |
| TJAP1 | 52.97 | 12.21 | 2.12 | 0.00029 | 41.15 | 10.12 | 2.02 | 0.00031 |
| ATG16L1 | 53.77 | 12.53 | 2.10 | 0.00029 | 68.87 | 16.97 | 2.02 | 0.00103 |
| GNA11 | 133.94 | 31.18 | 2.10 | 0.00029 | 83.50 | 20.61 | 2.02 | 0.00031 |
| RNF149 | 49.37 | 11.57 | 2.09 | 0.00029 | 42.83 | 10.58 | 2.02 | 0.00031 |
| IKZF5 | 15.92 | 3.23 | 2.30 | 0.00029 | 14.10 | 3.50 | 2.01 | 0.00031 |
| STOML2 | 515.62 | 99.63 | 2.37 | 0.00029 | 265.32 | 66.11 | 2.00 | 0.00031 |
| FLRT2 | 3.68 | 0.73 | 2.33 | 0.00029 | 3.73 | 0.93 | 2.00 | 0.00145 |
| PCBP1 | 236.82 | 34.56 | 2.78 | 0.00029 | 206.27 | 51.50 | 2.00 | 0.00031 |
