# Supplementary material for: Identification of tumorigenesis-related mRNAs associated with RNA-binding protein HuR in thyroid cancer cells
Source: Oncotarget. 2016 Aug 12;7(39):63388–407. doi: 10.18632/oncotarget.11255 (PMC5325372; doi:10.18632/oncotarget.11255)
Supplement: Supplementary file 5 [file oncotarget-07-63388-s005.docx]

*Supplementary Table 4. Nthy-ori-3.1 specific HuR interaction target RNA*

| Gene | HuR FPKM | IgG FPKM | Fold Change (log2) | q value |
| --- | --- | --- | --- | --- |
| ANP32D | 0.84 | 0.00 | Infinite | 0.03574 |
| LINC00266-1 | 0.80 | 0.00 | Infinite | 0.00075 |
| MIR3916 | 81.84 | 0.00 | Infinite | 0.04542 |
| NCOR1P1 | 1.12 | 0.00 | Infinite | 0.01434 |
| MSI1 | 5.45 | 0.22 | 4.65 | 0.00224 |
| EIF5AL1 | 1.68 | 0.11 | 3.94 | 0.01118 |
| WIPF3 | 1.34 | 0.10 | 3.70 | 0.02591 |
| KCNQ2 | 1.19 | 0.10 | 3.64 | 0.01227 |
| JUND | 364.07 | 33.39 | 3.45 | 0.00029 |
| PVRL4 | 4.57 | 0.44 | 3.36 | 0.00029 |
| OSTCP1 | 6.68 | 0.67 | 3.33 | 0.00171 |
| CXCL2 | 6.14 | 0.63 | 3.30 | 0.00189 |
| ITM2A | 3.03 | 0.32 | 3.26 | 0.00771 |
| GNG4 | 30.96 | 3.36 | 3.21 | 0.00029 |
| SERF1B | 1.59 | 0.17 | 3.20 | 0.02151 |
| LOC643770 | 1.85 | 0.20 | 3.18 | 0.04330 |
| TNFRSF13C | 3.29 | 0.37 | 3.13 | 0.02140 |
| EFNA2 | 19.89 | 2.28 | 3.12 | 0.00029 |
| ZNF703 | 6.96 | 0.86 | 3.01 | 0.00029 |
| LOC100131551 | 1.45 | 0.18 | 3.00 | 0.03177 |
| KLK7 | 1.01 | 0.13 | 2.93 | 0.02918 |
| CCL2 | 52.60 | 6.98 | 2.91 | 0.00029 |
| LOC100505783 | 31.93 | 4.33 | 2.88 | 0.00029 |
| PDE7B | 1.38 | 0.19 | 2.88 | 0.00115 |
| ULBP3 | 11.01 | 1.53 | 2.84 | 0.00520 |
| RASSF3 | 76.75 | 10.89 | 2.82 | 0.00029 |
| LOC100505495 | 0.83 | 0.12 | 2.81 | 0.02358 |
| CSNK1E | 240.77 | 34.57 | 2.80 | 0.00029 |
| RNF122 | 3.85 | 0.56 | 2.79 | 0.00400 |
| CAMKMT | 11.43 | 1.68 | 2.77 | 0.00029 |
| SULT1E1 | 8.62 | 1.27 | 2.76 | 0.00153 |
| METTL7A | 10.01 | 1.48 | 2.75 | 0.00029 |
| MGARP | 31.71 | 4.75 | 2.74 | 0.00029 |
| FLYWCH2 | 265.09 | 39.90 | 2.73 | 0.00029 |
| LAT2 | 1.57 | 0.24 | 2.71 | 0.01434 |
| GABARAPL2 | 218.56 | 33.48 | 2.71 | 0.00029 |
| MICB | 139.60 | 21.53 | 2.70 | 0.00029 |
| NBR2 | 12.88 | 2.01 | 2.68 | 0.00029 |
| MIDN | 26.93 | 4.22 | 2.67 | 0.00029 |
| GEMIN2 | 64.38 | 10.14 | 2.67 | 0.00029 |
| MAP2K3 | 45.87 | 7.23 | 2.67 | 0.00029 |
| MIR31HG | 3.64 | 0.58 | 2.66 | 0.00290 |
| RASL11A | 3.26 | 0.52 | 2.66 | 0.00715 |
| SPATA2 | 41.11 | 6.54 | 2.65 | 0.00029 |
| MAP1LC3C | 85.27 | 13.76 | 2.63 | 0.00029 |
| LOC100292680 | 7.25 | 1.17 | 2.63 | 0.00029 |
| APOBEC3D | 7.95 | 1.28 | 2.63 | 0.00029 |
| PAX6 | 0.81 | 0.13 | 2.62 | 0.00563 |
| ZSWIM6 | 25.81 | 4.22 | 2.61 | 0.00029 |
| TMEM169 | 12.16 | 2.04 | 2.58 | 0.00029 |
| OLR1 | 11.10 | 1.86 | 2.58 | 0.00029 |
| C10orf114 | 9.61 | 1.62 | 2.57 | 0.00029 |
| RANBP1 | 1935.47 | 326.66 | 2.57 | 0.00029 |
| TNPO2 | 88.88 | 15.13 | 2.55 | 0.00029 |
| SHOX2 | 7.74 | 1.32 | 2.55 | 0.00029 |
| PTP4A3 | 25.82 | 4.41 | 2.55 | 0.00029 |
| R3HDM2 | 71.05 | 12.17 | 2.55 | 0.00029 |
| SH3KBP1 | 75.67 | 12.98 | 2.54 | 0.00029 |
| ELOVL7 | 4.85 | 0.83 | 2.54 | 0.00029 |
| AKIRIN2 | 37.40 | 6.52 | 2.52 | 0.00029 |
| ENO2 | 189.65 | 33.26 | 2.51 | 0.00029 |
| FKBP1A | 508.62 | 89.87 | 2.50 | 0.00029 |
| ZCCHC3 | 58.96 | 10.46 | 2.50 | 0.00029 |
| CEBPB | 40.41 | 7.19 | 2.49 | 0.00029 |
| CXADRP2 | 1.94 | 0.35 | 2.49 | 0.00852 |
| SLC48A1 | 55.16 | 9.85 | 2.49 | 0.00029 |
| CAMTA1 | 178.90 | 32.31 | 2.47 | 0.00029 |
| LOC284276 | 2.98 | 0.54 | 2.47 | 0.00353 |
| LOC283683 | 8.02 | 1.46 | 2.46 | 0.00153 |
| HLF | 2.83 | 0.52 | 2.46 | 0.00053 |
| BASP1 | 425.87 | 78.20 | 2.45 | 0.00029 |
| TMSB4X | 5233.13 | 964.61 | 2.44 | 0.00029 |
| CSNK1G3 | 65.84 | 12.17 | 2.44 | 0.00029 |
| WNT3 | 13.76 | 2.56 | 2.43 | 0.00029 |
| LPAR3 | 1.34 | 0.25 | 2.42 | 0.03730 |
| B4GALT1 | 101.04 | 18.99 | 2.41 | 0.00029 |
| CITED2 | 407.46 | 76.61 | 2.41 | 0.00029 |
| LSM1 | 109.46 | 20.59 | 2.41 | 0.00876 |
| PRKACA | 172.37 | 32.44 | 2.41 | 0.00029 |
| LOC100288842 | 5.31 | 1.00 | 2.40 | 0.03837 |
| SSR4P1 | 3.88 | 0.73 | 2.40 | 0.00171 |
| NPPB | 25.92 | 4.94 | 2.39 | 0.00171 |
| ZNF578 | 1.06 | 0.20 | 2.39 | 0.00306 |
| KCNIP4 | 6.11 | 1.17 | 2.38 | 0.00029 |
| PPP4C | 470.88 | 90.99 | 2.37 | 0.00029 |
| BIRC5 | 386.58 | 74.70 | 2.37 | 0.00029 |
| RUNX1 | 35.34 | 6.84 | 2.37 | 0.00029 |
| CHTOP | 87.30 | 16.91 | 2.37 | 0.00171 |
| PADI2 | 2.40 | 0.46 | 2.37 | 0.00095 |
| LAPTM4A | 488.97 | 94.97 | 2.36 | 0.00029 |
| MASP2 | 2.01 | 0.39 | 2.36 | 0.00460 |
| MESDC1 | 27.92 | 5.43 | 2.36 | 0.00029 |
| TP53 | 247.53 | 48.20 | 2.36 | 0.00029 |
| LPCAT4 | 43.99 | 8.57 | 2.36 | 0.00029 |
| LOC339803 | 20.26 | 3.96 | 2.35 | 0.00029 |
| NAV1 | 34.60 | 6.77 | 2.35 | 0.00029 |
| LHFP | 68.59 | 13.45 | 2.35 | 0.00029 |
| TWSG1 | 166.50 | 32.67 | 2.35 | 0.00029 |
| RCC2 | 283.41 | 55.92 | 2.34 | 0.00029 |
| TMEM178A | 2.03 | 0.40 | 2.34 | 0.03147 |
| CNOT6 | 77.97 | 15.42 | 2.34 | 0.00029 |
| IKZF2 | 5.97 | 1.18 | 2.34 | 0.00029 |
| SPRY2 | 12.02 | 2.38 | 2.34 | 0.00029 |
| AKIP1 | 49.95 | 9.91 | 2.33 | 0.00029 |
| TMEM59 | 261.83 | 52.17 | 2.33 | 0.00029 |
| COX7A2L | 487.27 | 97.17 | 2.33 | 0.00029 |
| CSK | 71.98 | 14.37 | 2.32 | 0.00029 |
| NFYC | 61.86 | 12.35 | 2.32 | 0.00029 |
| KAZN | 43.75 | 8.77 | 2.32 | 0.00029 |
| C9orf41 | 57.51 | 11.55 | 2.32 | 0.00029 |
| USP2 | 6.90 | 1.39 | 2.31 | 0.00029 |
| LINC00294 | 30.70 | 6.19 | 2.31 | 0.00029 |
| COX16 | 78.75 | 15.89 | 2.31 | 0.00812 |
| RPIA | 27.22 | 5.50 | 2.31 | 0.00029 |
| SCARNA7 | 4402.03 | 889.50 | 2.31 | 0.00258 |
| PBX2 | 89.31 | 18.05 | 2.31 | 0.00029 |
| SMAD9 | 22.28 | 4.53 | 2.30 | 0.00029 |
| AP3S1 | 220.41 | 44.85 | 2.30 | 0.00029 |
| LINC00663 | 1.47 | 0.30 | 2.29 | 0.01506 |
| TINAGL1 | 168.76 | 34.47 | 2.29 | 0.00029 |
| SCD5 | 40.58 | 8.30 | 2.29 | 0.00029 |
| FAM192A | 103.78 | 21.27 | 2.29 | 0.00029 |
| CHRNA7 | 1.22 | 0.25 | 2.29 | 0.02591 |
| YPEL5 | 74.54 | 15.33 | 2.28 | 0.00029 |
| PRMT6 | 37.45 | 7.71 | 2.28 | 0.00029 |
| PTGER4P2-CDK2AP2P2 | 1.42 | 0.29 | 2.28 | 0.00578 |
| PFN1 | 2589.07 | 535.65 | 2.27 | 0.00029 |
| LOC100131655 | 3.04 | 0.63 | 2.27 | 0.01757 |
| KCNMB4 | 2.19 | 0.45 | 2.27 | 0.00290 |
| RABL2A | 2.38 | 0.49 | 2.27 | 0.00592 |
| PPIC | 109.27 | 22.64 | 2.27 | 0.00029 |
| AES | 350.75 | 72.67 | 2.27 | 0.00029 |
| DLC1 | 121.73 | 25.25 | 2.27 | 0.00029 |
| MGC57346 | 47.53 | 9.86 | 2.27 | 0.00029 |
| SLC52A1 | 2.79 | 0.58 | 2.27 | 0.00606 |
| CCRN4L | 26.70 | 5.55 | 2.27 | 0.00029 |
| GSG1 | 11.70 | 2.44 | 2.26 | 0.00029 |
| AGTR1 | 1.24 | 0.26 | 2.26 | 0.01080 |
| HIBADH | 113.29 | 23.73 | 2.26 | 0.00029 |
| PRRG4 | 3.94 | 0.83 | 2.25 | 0.00053 |
| RAB14 | 46.02 | 9.69 | 2.25 | 0.00029 |
| ZBTB34 | 13.81 | 2.91 | 2.25 | 0.00029 |
| NEBL | 2.04 | 0.43 | 2.25 | 0.00029 |
| ARHGEF25 | 59.53 | 12.58 | 2.24 | 0.00029 |
| CSDA | 193.67 | 41.03 | 2.24 | 0.00029 |
| MED28 | 98.03 | 20.78 | 2.24 | 0.00029 |
| UBTD2 | 62.43 | 13.26 | 2.24 | 0.00029 |
| ATPBD4 | 9.27 | 1.97 | 2.24 | 0.00053 |
| REXO1 | 17.59 | 3.74 | 2.23 | 0.00029 |
| VMAC | 3.07 | 0.66 | 2.23 | 0.01481 |
| BRD4 | 63.76 | 13.62 | 2.23 | 0.00029 |
| IER2 | 29.92 | 6.40 | 2.23 | 0.00029 |
| NFIX | 46.84 | 10.02 | 2.22 | 0.00029 |
| TRIM8 | 152.45 | 32.85 | 2.21 | 0.00029 |
| LOC100506190 | 25.03 | 5.40 | 2.21 | 0.00029 |
| HEATR3 | 33.60 | 7.25 | 2.21 | 0.00029 |
| C15orf48 | 21.62 | 4.67 | 2.21 | 0.00115 |
| MAT2B | 126.47 | 27.32 | 2.21 | 0.00029 |
| SLC35F1 | 1.25 | 0.27 | 2.21 | 0.00676 |
| PFN2 | 425.58 | 92.19 | 2.21 | 0.00029 |
| PHACTR4 | 64.09 | 13.90 | 2.21 | 0.00029 |
| CXADR | 63.24 | 13.72 | 2.20 | 0.00029 |
| DAZAP2 | 395.45 | 85.79 | 2.20 | 0.00029 |
| PLAC8 | 275.61 | 59.88 | 2.20 | 0.00029 |
| PIP4K2A | 105.16 | 22.97 | 2.19 | 0.00029 |
| CDR2L | 44.90 | 9.81 | 2.19 | 0.00029 |
| LOC494141 | 3.12 | 0.68 | 2.19 | 0.01204 |
| SKIDA1 | 7.31 | 1.60 | 2.19 | 0.00029 |
| YIPF2 | 134.72 | 29.60 | 2.19 | 0.00029 |
| ULBP1 | 1.55 | 0.34 | 2.18 | 0.01457 |
| APOBEC3C | 28.77 | 6.34 | 2.18 | 0.00029 |
| OSR2 | 4.59 | 1.01 | 2.18 | 0.00290 |
| CCDC6 | 63.24 | 13.95 | 2.18 | 0.00029 |
| POU2AF1 | 0.96 | 0.21 | 2.18 | 0.04523 |
| DLEU2L | 31.44 | 6.97 | 2.17 | 0.00075 |
| HIST1H4H | 4703.60 | 1043.29 | 2.17 | 0.00029 |
| TVP23B | 16.81 | 3.73 | 2.17 | 0.00029 |
| GCH1 | 14.38 | 3.19 | 2.17 | 0.00053 |
| GJC1 | 68.87 | 15.32 | 2.17 | 0.00029 |
| GXYLT2 | 91.31 | 20.37 | 2.16 | 0.00029 |
| SLC8A1 | 36.46 | 8.14 | 2.16 | 0.00029 |
| LSR | 15.39 | 3.45 | 2.16 | 0.00029 |
| TLCD2 | 25.58 | 5.73 | 2.16 | 0.00029 |
| PHLDA1 | 16.74 | 3.75 | 2.16 | 0.00029 |
| PTDSS1 | 120.92 | 27.19 | 2.15 | 0.00029 |
| COTL1 | 293.85 | 66.20 | 2.15 | 0.00029 |
| NFIC | 32.19 | 7.28 | 2.15 | 0.00029 |
| STEAP4 | 3.24 | 0.73 | 2.15 | 0.00095 |
| TEAD2 | 61.34 | 13.87 | 2.14 | 0.00029 |
| FRS3 | 7.96 | 1.80 | 2.14 | 0.00029 |
| MID1IP1 | 28.36 | 6.43 | 2.14 | 0.00029 |
| KBTBD12 | 2.99 | 0.68 | 2.14 | 0.00241 |
| RAB39A | 8.92 | 2.02 | 2.14 | 0.00053 |
| FAM108C1 | 14.84 | 3.37 | 2.14 | 0.00029 |
| FJX1 | 25.13 | 5.72 | 2.14 | 0.00029 |
| MAX | 78.25 | 17.80 | 2.14 | 0.00224 |
| LOC100506394 | 30.70 | 6.99 | 2.14 | 0.00029 |
| C20orf112 | 51.63 | 11.78 | 2.13 | 0.00029 |
| ELOVL4 | 2.72 | 0.62 | 2.12 | 0.00460 |
| KDELR2 | 502.04 | 115.13 | 2.12 | 0.00029 |
| GSDMC | 0.79 | 0.18 | 2.12 | 0.04919 |
| SLC35E2 | 14.98 | 3.46 | 2.12 | 0.00075 |
| NCOA5 | 69.82 | 16.14 | 2.11 | 0.00029 |
| LINC00339 | 31.24 | 7.23 | 2.11 | 0.00029 |
| YBX2 | 9.77 | 2.26 | 2.11 | 0.00053 |
| LINC00471 | 3.88 | 0.90 | 2.11 | 0.00980 |
| C4orf46 | 51.35 | 11.92 | 2.11 | 0.00029 |
| ZFHX3 | 11.85 | 2.75 | 2.11 | 0.00029 |
| MTFR2 | 11.16 | 2.60 | 2.10 | 0.00053 |
| LINC00629 | 2.56 | 0.60 | 2.10 | 0.02792 |
| BEND3P3 | 3.68 | 0.86 | 2.10 | 0.00053 |
| NRSN1 | 1.38 | 0.32 | 2.10 | 0.02442 |
| LOC151475 | 0.85 | 0.20 | 2.09 | 0.04605 |
| LOC100128288 | 2.52 | 0.59 | 2.09 | 0.00876 |
| HCG4 | 1.69 | 0.40 | 2.09 | 0.03657 |
| TFDP1 | 315.50 | 74.10 | 2.09 | 0.00029 |
| DOLPP1 | 66.65 | 15.67 | 2.09 | 0.00029 |
| CEBPG | 17.05 | 4.01 | 2.09 | 0.00029 |
| SLC7A8 | 0.78 | 0.18 | 2.08 | 0.03336 |
| KCNK1 | 2.29 | 0.54 | 2.08 | 0.00876 |
| RBMS2 | 55.11 | 13.02 | 2.08 | 0.00275 |
| LOC100499405 | 3.82 | 0.90 | 2.08 | 0.00520 |
| GOLPH3 | 155.11 | 36.67 | 2.08 | 0.00029 |
| ZNF468 | 8.66 | 2.05 | 2.08 | 0.00029 |
| CAMK2N1 | 3.93 | 0.93 | 2.08 | 0.00430 |
| TBX3 | 5.53 | 1.31 | 2.08 | 0.00029 |
| MGST3 | 141.65 | 33.58 | 2.08 | 0.00029 |
| SRSF8 | 15.51 | 3.69 | 2.07 | 0.00029 |
| CYP1A2 | 3.62 | 0.86 | 2.07 | 0.00095 |
| RAB19 | 23.96 | 5.70 | 2.07 | 0.00207 |
| XIAP | 33.90 | 8.07 | 2.07 | 0.00029 |
| STX1B | 2.09 | 0.50 | 2.07 | 0.00321 |
| PGAM5 | 87.84 | 20.93 | 2.07 | 0.00029 |
| JDP2 | 32.49 | 7.75 | 2.07 | 0.00029 |
| ZNF674-AS1 | 4.20 | 1.00 | 2.07 | 0.00368 |
| B3GALTL | 43.31 | 10.34 | 2.07 | 0.00029 |
| ING3 | 27.84 | 6.65 | 2.07 | 0.00029 |
| TNFRSF9 | 3.40 | 0.81 | 2.06 | 0.00053 |
| RAB7A | 220.66 | 52.79 | 2.06 | 0.00029 |
| RAB6A | 90.33 | 21.62 | 2.06 | 0.00029 |
| SUZ12P1 | 36.43 | 8.72 | 2.06 | 0.00095 |
| UBE2C | 1732.13 | 414.93 | 2.06 | 0.00029 |
| RYBP | 38.94 | 9.33 | 2.06 | 0.00029 |
| STX11 | 7.94 | 1.91 | 2.06 | 0.00029 |
| ZNF738 | 2.29 | 0.55 | 2.06 | 0.00029 |
| C4orf32 | 2.17 | 0.52 | 2.06 | 0.01068 |
| XYLT2 | 78.75 | 18.98 | 2.05 | 0.00029 |
| PRR16 | 3.04 | 0.73 | 2.05 | 0.01043 |
| PXDC1 | 150.95 | 36.53 | 2.05 | 0.00029 |
| ARPC1A | 519.66 | 125.90 | 2.05 | 0.00029 |
| ZC3HAV1L | 59.20 | 14.36 | 2.04 | 0.00029 |
| BRD3 | 38.31 | 9.31 | 2.04 | 0.00029 |
| LYRM1 | 61.76 | 15.01 | 2.04 | 0.01531 |
| SNX3 | 408.43 | 99.50 | 2.04 | 0.00029 |
| SIAH1 | 34.61 | 8.43 | 2.04 | 0.01846 |
| SEPT11 | 117.84 | 28.74 | 2.04 | 0.00029 |
| AMD1 | 121.12 | 29.55 | 2.04 | 0.00029 |
| DMD | 13.21 | 3.23 | 2.03 | 0.00029 |
| UBE2M | 123.35 | 30.14 | 2.03 | 0.00029 |
| NUAK2 | 26.88 | 6.58 | 2.03 | 0.00029 |
| C3orf33 | 4.13 | 1.01 | 2.03 | 0.00606 |
| CDKL1 | 14.50 | 3.56 | 2.03 | 0.00171 |
| RBM47 | 4.62 | 1.14 | 2.02 | 0.00029 |
| ADAM19 | 34.62 | 8.53 | 2.02 | 0.00029 |
| SMAD6 | 40.78 | 10.07 | 2.02 | 0.00029 |
| MB21D1 | 21.01 | 5.19 | 2.02 | 0.00029 |
| ADRBK1 | 64.43 | 15.92 | 2.02 | 0.00029 |
| NACC1 | 114.06 | 28.22 | 2.02 | 0.00029 |
| ZNF284 | 3.81 | 0.95 | 2.01 | 0.00353 |
| GMCL1P1 | 1.57 | 0.39 | 2.01 | 0.04575 |
| PLEKHF2 | 25.03 | 6.23 | 2.01 | 0.00029 |
| SETD5-AS1 | 11.72 | 2.92 | 2.00 | 0.00029 |
| ZNF76 | 37.14 | 9.26 | 2.00 | 0.00029 |
| MYC | 95.77 | 23.90 | 2.00 | 0.00029 |
