# Supplementary material for: Identification of tumorigenesis-related mRNAs associated with RNA-binding protein HuR in thyroid cancer cells
Source: Oncotarget. 2016 Aug 12;7(39):63388–407. doi: 10.18632/oncotarget.11255 (PMC5325372; doi:10.18632/oncotarget.11255)
Supplement: Supplementary file 6 [file oncotarget-07-63388-s006.docx]

*Supplementary Table 5. BCPAP specific HuR interaction target RNA*

| Gene | HuR FPKM | IgG FPKM | Fold Change (log2) | q value |
| --- | --- | --- | --- | --- |
| GHRL | 1.08 | 0.00 | Infinite | 0.00081 |
| GNAT2 | 1.22 | 0.00 | Infinite | 0.00031 |
| HCST | 5.43 | 0.00 | Infinite | 0.00031 |
| LOC285540 | 1.48 | 0.00 | Infinite | 0.00031 |
| MAP1LC3B2 | 0.99 | 0.00 | Infinite | 0.00406 |
| MRAP2 | 1.16 | 0.00 | Infinite | 0.00031 |
| NRN1 | 1.66 | 0.00 | Infinite | 0.00031 |
| PFN4 | 3.04 | 0.00 | Infinite | 0.00031 |
| ZNF826P | 1.81 | 0.00 | Infinite | 0.00341 |
| C16orf54 | 10.39 | 0.75 | 3.80 | 0.00031 |
| EXPH5 | 12.26 | 1.08 | 3.50 | 0.00031 |
| LOC100131067 | 18.79 | 1.73 | 3.44 | 0.01602 |
| ZSCAN12 | 2.71 | 0.25 | 3.41 | 0.00203 |
| DMRTA1 | 3.07 | 0.30 | 3.34 | 0.01835 |
| MGC16275 | 3.00 | 0.30 | 3.31 | 0.02577 |
| LOC100505648 | 1.97 | 0.20 | 3.31 | 0.02727 |
| LOC284260 | 1.08 | 0.11 | 3.30 | 0.03251 |
| ATF7IP2 | 1.40 | 0.14 | 3.27 | 0.03384 |
| HOGA1 | 2.17 | 0.24 | 3.19 | 0.02408 |
| GCNT4 | 3.06 | 0.34 | 3.19 | 0.02046 |
| GRIPAP1 | 97.90 | 10.75 | 3.19 | 0.00031 |
| ZNF624 | 3.23 | 0.36 | 3.15 | 0.00103 |
| LOC152225 | 9.08 | 1.03 | 3.14 | 0.00103 |
| TBX20 | 11.60 | 1.33 | 3.12 | 0.00031 |
| KLHL15 | 17.88 | 2.13 | 3.07 | 0.00031 |
| GLCE | 39.65 | 4.75 | 3.06 | 0.00031 |
| TSSK6 | 2.70 | 0.33 | 3.05 | 0.03755 |
| TSPYL1 | 66.73 | 8.12 | 3.04 | 0.00031 |
| REST | 77.02 | 9.42 | 3.03 | 0.00031 |
| UBA6 | 246.89 | 30.64 | 3.01 | 0.00031 |
| PUS7L | 42.97 | 5.41 | 2.99 | 0.00031 |
| PM20D2 | 10.85 | 1.38 | 2.98 | 0.00031 |
| LOC441155 | 11.30 | 1.45 | 2.96 | 0.00031 |
| PPP1R14C | 22.10 | 2.88 | 2.94 | 0.00031 |
| ZNF805 | 25.45 | 3.32 | 2.94 | 0.00031 |
| FOXN2 | 30.33 | 4.01 | 2.92 | 0.00031 |
| MSR1 | 11.22 | 1.49 | 2.91 | 0.00031 |
| FAM210A | 29.01 | 3.89 | 2.90 | 0.00031 |
| SLC39A10 | 22.32 | 3.00 | 2.90 | 0.00031 |
| FRRS1 | 3.15 | 0.43 | 2.87 | 0.04268 |
| MAF | 1.48 | 0.21 | 2.81 | 0.02514 |
| GP6 | 1.62 | 0.23 | 2.80 | 0.03582 |
| ZNF460 | 93.27 | 13.49 | 2.79 | 0.00031 |
| TRIM59 | 18.40 | 2.68 | 2.78 | 0.00031 |
| ZBTB41 | 21.59 | 3.15 | 2.78 | 0.00031 |
| ISPD | 1.28 | 0.19 | 2.76 | 0.01801 |
| ITGB1 | 801.28 | 118.27 | 2.76 | 0.00031 |
| TMEM144 | 11.95 | 1.78 | 2.74 | 0.00031 |
| MIER1 | 84.39 | 12.63 | 2.74 | 0.00031 |
| FRG2 | 102.03 | 15.33 | 2.73 | 0.00031 |
| PHF6 | 113.24 | 17.08 | 2.73 | 0.00031 |
| BTC | 5.63 | 0.85 | 2.73 | 0.01372 |
| CRLF3 | 47.58 | 7.20 | 2.72 | 0.00031 |
| CEP44 | 11.16 | 1.69 | 2.72 | 0.00031 |
| LOC93622 | 5.10 | 0.78 | 2.71 | 0.01198 |
| IRGQ | 36.03 | 5.51 | 2.71 | 0.00031 |
| LOC201651 | 63.86 | 9.80 | 2.70 | 0.00031 |
| SLC6A15 | 57.02 | 8.76 | 2.70 | 0.00031 |
| SOX5 | 2.09 | 0.32 | 2.69 | 0.00689 |
| SPIN4 | 10.81 | 1.68 | 2.69 | 0.00031 |
| GLS | 90.16 | 14.14 | 2.67 | 0.00031 |
| EREG | 277.96 | 43.78 | 2.67 | 0.00031 |
| THBS1 | 41.29 | 6.52 | 2.66 | 0.00031 |
| PTEN | 124.96 | 19.85 | 2.65 | 0.00031 |
| FGF5 | 18.52 | 2.96 | 2.65 | 0.00031 |
| TM4SF1 | 143.78 | 22.98 | 2.65 | 0.00031 |
| THAP5 | 31.63 | 5.07 | 2.64 | 0.00031 |
| TXNDC16 | 2.42 | 0.39 | 2.64 | 0.00604 |
| RBM12B | 12.42 | 1.99 | 2.64 | 0.00031 |
| SFR1 | 28.85 | 4.64 | 2.64 | 0.00031 |
| SLC41A2 | 7.86 | 1.27 | 2.63 | 0.00031 |
| EZR | 260.89 | 42.12 | 2.63 | 0.00031 |
| KIF3A | 23.00 | 3.71 | 2.63 | 0.00031 |
| ATP6V0D2 | 44.87 | 7.25 | 2.63 | 0.00031 |
| SNX10 | 3.51 | 0.57 | 2.63 | 0.00576 |
| FAM161A | 4.34 | 0.71 | 2.62 | 0.00103 |
| TMEM168 | 7.60 | 1.25 | 2.61 | 0.00031 |
| TTI2 | 85.12 | 14.01 | 2.60 | 0.00221 |
| APOL2 | 18.92 | 3.12 | 2.60 | 0.00031 |
| FUT1 | 1.57 | 0.26 | 2.60 | 0.02918 |
| PPM1L | 4.45 | 0.74 | 2.59 | 0.00545 |
| PCBD2 | 22.59 | 3.76 | 2.59 | 0.00103 |
| C8orf4 | 27.11 | 4.51 | 2.59 | 0.00031 |
| FAM103A1 | 51.79 | 8.63 | 2.59 | 0.00031 |
| ST8SIA4 | 5.82 | 0.97 | 2.59 | 0.00031 |
| MYBL1 | 3.78 | 0.63 | 2.58 | 0.00184 |
| ERBB2IP | 154.38 | 25.95 | 2.57 | 0.00031 |
| PCMTD1 | 27.53 | 4.63 | 2.57 | 0.00031 |
| PRKAR1A | 489.34 | 82.34 | 2.57 | 0.00031 |
| FAM171B | 21.25 | 3.59 | 2.57 | 0.00031 |
| LOC646719 | 17.05 | 2.88 | 2.57 | 0.00031 |
| ZNF165 | 10.39 | 1.76 | 2.56 | 0.00103 |
| EEF2K | 11.12 | 1.89 | 2.56 | 0.00031 |
| NFIB | 5.32 | 0.91 | 2.54 | 0.00031 |
| CYP51A1 | 71.94 | 12.40 | 2.54 | 0.00031 |
| ADAM10 | 98.34 | 17.00 | 2.53 | 0.00031 |
| CHRAC1 | 52.08 | 9.01 | 2.53 | 0.00031 |
| RORA | 8.14 | 1.41 | 2.53 | 0.00031 |
| EPM2AIP1 | 9.54 | 1.66 | 2.53 | 0.00031 |
| PHACTR2 | 23.33 | 4.06 | 2.52 | 0.00031 |
| FAM111A | 55.76 | 9.72 | 2.52 | 0.00031 |
| SRBD1 | 33.76 | 5.90 | 2.52 | 0.00031 |
| CCDC122 | 18.24 | 3.19 | 2.52 | 0.00545 |
| ZNF311 | 1.79 | 0.31 | 2.52 | 0.03919 |
| ESCO2 | 9.71 | 1.70 | 2.51 | 0.00031 |
| THUMPD1 | 44.08 | 7.73 | 2.51 | 0.00031 |
| SHISA2 | 70.02 | 12.33 | 2.51 | 0.00031 |
| ZNF486 | 2.75 | 0.48 | 2.50 | 0.00661 |
| ZNF490 | 18.26 | 3.22 | 2.50 | 0.00031 |
| THRAP3 | 171.03 | 30.17 | 2.50 | 0.00031 |
| HLTF | 40.67 | 7.19 | 2.50 | 0.00031 |
| HDAC2 | 91.02 | 16.08 | 2.50 | 0.00031 |
| DTX3L | 62.74 | 11.09 | 2.50 | 0.00031 |
| THAP1 | 23.97 | 4.25 | 2.49 | 0.00031 |
| BMPR1B | 11.59 | 2.06 | 2.49 | 0.00031 |
| PPM1K | 11.34 | 2.02 | 2.49 | 0.00031 |
| LIMCH1 | 33.78 | 6.03 | 2.49 | 0.00031 |
| ERI2 | 4.45 | 0.80 | 2.48 | 0.01591 |
| SLITRK1 | 13.07 | 2.34 | 2.48 | 0.00031 |
| NOL9 | 83.45 | 14.94 | 2.48 | 0.00031 |
| EVI5 | 41.84 | 7.53 | 2.47 | 0.00031 |
| RYK | 118.94 | 21.40 | 2.47 | 0.00031 |
| LOC100506730 | 4.63 | 0.83 | 2.47 | 0.00391 |
| STK38L | 14.43 | 2.60 | 2.47 | 0.00031 |
| FUBP1 | 190.07 | 34.27 | 2.47 | 0.00031 |
| GAN | 20.64 | 3.72 | 2.47 | 0.00031 |
| DDX17 | 284.92 | 51.38 | 2.47 | 0.00031 |
| CCDC43 | 107.26 | 19.36 | 2.47 | 0.00031 |
| PPP1R15B | 123.73 | 22.43 | 2.46 | 0.00031 |
| NAA15 | 79.97 | 14.54 | 2.46 | 0.00031 |
| YES1 | 103.59 | 18.84 | 2.46 | 0.00031 |
| SOCS4 | 26.58 | 4.85 | 2.45 | 0.00031 |
| NAA30 | 48.14 | 8.79 | 2.45 | 0.00031 |
| TRPM7 | 65.94 | 12.09 | 2.45 | 0.00031 |
| MEIS3P1 | 27.85 | 5.11 | 2.45 | 0.00031 |
| GTPBP10 | 10.85 | 2.00 | 2.44 | 0.00031 |
| ZNF626 | 23.88 | 4.41 | 2.44 | 0.00124 |
| GOLIM4 | 66.02 | 12.19 | 2.44 | 0.00031 |
| UTP15 | 36.17 | 6.70 | 2.43 | 0.00031 |
| TMEM209 | 50.30 | 9.32 | 2.43 | 0.00031 |
| HNRNPU | 276.97 | 51.39 | 2.43 | 0.00031 |
| EDEM3 | 56.78 | 10.55 | 2.43 | 0.00031 |
| SH3BGRL2 | 21.28 | 3.96 | 2.43 | 0.00031 |
| LIN54 | 15.22 | 2.84 | 2.42 | 0.00031 |
| GNRHR2 | 18.64 | 3.49 | 2.42 | 0.00164 |
| DYNC1LI2 | 67.75 | 12.75 | 2.41 | 0.00031 |
| ALG10 | 6.08 | 1.15 | 2.41 | 0.00358 |
| FNIP1 | 105.40 | 19.91 | 2.40 | 0.00031 |
| SLX4IP | 20.98 | 3.96 | 2.40 | 0.00081 |
| CTDSPL2 | 38.51 | 7.35 | 2.39 | 0.00031 |
| UPF2 | 45.52 | 8.70 | 2.39 | 0.00031 |
| CEP41 | 8.86 | 1.70 | 2.38 | 0.00031 |
| SLC16A6 | 24.86 | 4.76 | 2.38 | 0.00184 |
| OCLN | 4.68 | 0.90 | 2.38 | 0.00057 |
| PSIP1 | 68.87 | 13.25 | 2.38 | 0.00031 |
| REPS2 | 15.18 | 2.92 | 2.38 | 0.00031 |
| FAM199X | 45.40 | 8.76 | 2.37 | 0.00031 |
| POU5F1 | 1.68 | 0.33 | 2.37 | 0.04790 |
| HIF1A-AS2 | 184.89 | 35.88 | 2.37 | 0.02986 |
| AXL | 789.79 | 153.33 | 2.36 | 0.00031 |
| TMX3 | 65.80 | 12.80 | 2.36 | 0.00031 |
| GAPVD1 | 55.46 | 10.79 | 2.36 | 0.00031 |
| RAB5A | 101.98 | 19.92 | 2.36 | 0.00031 |
| WDR43 | 123.08 | 24.08 | 2.35 | 0.00031 |
| ORAOV1 | 14.15 | 2.77 | 2.35 | 0.00031 |
| MBOAT1 | 6.41 | 1.26 | 2.35 | 0.00031 |
| PKN2 | 59.60 | 11.74 | 2.34 | 0.00031 |
| MTX3 | 52.26 | 10.36 | 2.33 | 0.00031 |
| TOPORS | 16.04 | 3.18 | 2.33 | 0.00031 |
| KIAA1715 | 19.80 | 3.94 | 2.33 | 0.00031 |
| KIAA1430 | 55.65 | 11.07 | 2.33 | 0.00031 |
| MPZL2 | 6.46 | 1.29 | 2.33 | 0.00341 |
| SDE2 | 39.58 | 7.88 | 2.33 | 0.00031 |
| SOGA1 | 58.71 | 11.71 | 2.33 | 0.00031 |
| C16orf87 | 7.67 | 1.53 | 2.32 | 0.01068 |
| C2CD2 | 23.29 | 4.66 | 2.32 | 0.00031 |
| CASC4 | 144.94 | 29.01 | 2.32 | 0.00031 |
| ZNF543 | 12.78 | 2.56 | 2.32 | 0.00031 |
| TMEM38B | 19.24 | 3.86 | 2.32 | 0.00031 |
| SSR3 | 348.09 | 70.01 | 2.31 | 0.00031 |
| TNRC6B | 17.66 | 3.55 | 2.31 | 0.00031 |
| SLC9A7 | 48.47 | 9.81 | 2.30 | 0.00031 |
| PLSCR4 | 1.84 | 0.37 | 2.30 | 0.01602 |
| ESM1 | 79.11 | 16.07 | 2.30 | 0.00031 |
| RDX | 141.03 | 28.66 | 2.30 | 0.00031 |
| SP3 | 87.94 | 17.96 | 2.29 | 0.00031 |
| SAMD12 | 5.19 | 1.06 | 2.29 | 0.00031 |
| YOD1 | 22.11 | 4.53 | 2.29 | 0.00031 |
| NTM | 147.63 | 30.23 | 2.29 | 0.00031 |
| RICTOR | 41.16 | 8.45 | 2.28 | 0.00031 |
| C8orf37 | 3.30 | 0.68 | 2.28 | 0.00358 |
| LOC389641 | 12.67 | 2.60 | 2.28 | 0.00031 |
| SMC5 | 25.91 | 5.33 | 2.28 | 0.00031 |
| CLCC1 | 27.68 | 5.70 | 2.28 | 0.00031 |
| ZFAND1 | 121.26 | 25.04 | 2.28 | 0.00031 |
| CUL5 | 52.10 | 10.80 | 2.27 | 0.00031 |
| SYT14 | 35.46 | 7.35 | 2.27 | 0.00031 |
| COQ10B | 60.09 | 12.46 | 2.27 | 0.00031 |
| ZKSCAN3 | 3.77 | 0.78 | 2.27 | 0.00145 |
| MANEA | 11.22 | 2.33 | 2.27 | 0.00031 |
| SAYSD1 | 9.85 | 2.04 | 2.27 | 0.00103 |
| KLHL11 | 51.23 | 10.62 | 2.27 | 0.00031 |
| PPP1R12A | 117.85 | 24.54 | 2.26 | 0.00031 |
| ZBTB10 | 14.52 | 3.03 | 2.26 | 0.00031 |
| FAM102B | 14.87 | 3.11 | 2.26 | 0.00031 |
| C2orf69 | 24.72 | 5.18 | 2.25 | 0.00031 |
| PCDHB14 | 2.50 | 0.52 | 2.25 | 0.02935 |
| HBS1L | 36.48 | 7.65 | 2.25 | 0.00031 |
| GK | 11.77 | 2.48 | 2.25 | 0.00031 |
| DENND6A | 36.67 | 7.73 | 2.25 | 0.00031 |
| LEF1 | 4.36 | 0.92 | 2.25 | 0.00923 |
| LBR | 37.09 | 7.82 | 2.25 | 0.00031 |
| DCUN1D4 | 35.41 | 7.47 | 2.24 | 0.00031 |
| DNAJC16 | 31.05 | 6.57 | 2.24 | 0.00031 |
| FLI1 | 42.44 | 8.99 | 2.24 | 0.00031 |
| TSNAX | 98.10 | 20.78 | 2.24 | 0.00031 |
| AGPHD1 | 6.24 | 1.32 | 2.24 | 0.03209 |
| ZNF814 | 9.24 | 1.96 | 2.24 | 0.00031 |
| PAIP2B | 3.30 | 0.70 | 2.23 | 0.00031 |
| ERO1L | 172.80 | 36.94 | 2.23 | 0.00031 |
| LOC100129550 | 2.67 | 0.57 | 2.23 | 0.00257 |
| HAS2 | 17.10 | 3.66 | 2.22 | 0.00031 |
| JRKL | 26.53 | 5.68 | 2.22 | 0.00031 |
| CHSY1 | 62.35 | 13.35 | 2.22 | 0.00031 |
| KLHL24 | 45.57 | 9.76 | 2.22 | 0.00031 |
| MYEF2 | 13.79 | 2.95 | 2.22 | 0.00031 |
| TMX1 | 94.28 | 20.21 | 2.22 | 0.00031 |
| IGFBP5 | 13.00 | 2.79 | 2.22 | 0.00031 |
| ZBTB1 | 52.17 | 11.21 | 2.22 | 0.00031 |
| WNT5A | 83.19 | 17.89 | 2.22 | 0.00031 |
| MAP3K1 | 11.83 | 2.54 | 2.22 | 0.00031 |
| PRPS2 | 89.72 | 19.36 | 2.21 | 0.00031 |
| C5orf51 | 51.39 | 11.10 | 2.21 | 0.00031 |
| B4GALT6 | 40.48 | 8.74 | 2.21 | 0.00031 |
| TMEM170B | 7.86 | 1.70 | 2.21 | 0.00031 |
| PER2 | 7.33 | 1.59 | 2.21 | 0.00031 |
| SUPV3L1 | 50.09 | 10.84 | 2.21 | 0.00031 |
| HOXA3 | 3.90 | 0.85 | 2.20 | 0.01186 |
| LOC100506599 | 4.09 | 0.89 | 2.20 | 0.00406 |
| SLC25A51 | 28.01 | 6.09 | 2.20 | 0.00031 |
| MRPL42 | 66.49 | 14.45 | 2.20 | 0.00031 |
| PRKCI | 81.40 | 17.71 | 2.20 | 0.00031 |
| SNTB2 | 14.03 | 3.05 | 2.20 | 0.00031 |
| CDKN2B | 5.59 | 1.22 | 2.20 | 0.00661 |
| CDS2 | 24.44 | 5.33 | 2.20 | 0.00031 |
| RNF169 | 37.31 | 8.13 | 2.20 | 0.00031 |
| SGK196 | 43.15 | 9.41 | 2.20 | 0.00031 |
| IMPAD1 | 101.32 | 22.12 | 2.20 | 0.00031 |
| BTBD9 | 10.60 | 2.32 | 2.19 | 0.00031 |
| SYDE2 | 4.84 | 1.06 | 2.19 | 0.00124 |
| C1orf115 | 12.70 | 2.78 | 2.19 | 0.00031 |
| TNFAIP8 | 21.62 | 4.73 | 2.19 | 0.00057 |
| GOLPH3L | 28.69 | 6.31 | 2.19 | 0.00031 |
| CDC73 | 32.67 | 7.21 | 2.18 | 0.00031 |
| FEZ1 | 89.86 | 19.84 | 2.18 | 0.00031 |
| STK17B | 40.49 | 8.94 | 2.18 | 0.00031 |
| BCL11A | 7.44 | 1.64 | 2.18 | 0.00391 |
| ZBTB25 | 13.07 | 2.89 | 2.18 | 0.00145 |
| TMTC4 | 5.48 | 1.21 | 2.18 | 0.00203 |
| ARAP2 | 8.46 | 1.87 | 2.18 | 0.00031 |
| TULP4 | 51.06 | 11.31 | 2.17 | 0.00031 |
| CDH6 | 11.82 | 2.62 | 2.17 | 0.00031 |
| FAM208A | 61.05 | 13.55 | 2.17 | 0.00031 |
| CADPS2 | 19.14 | 4.26 | 2.17 | 0.00031 |
| SNX27 | 31.84 | 7.10 | 2.17 | 0.00031 |
| RAB3D | 40.57 | 9.04 | 2.17 | 0.00031 |
| ZNF772 | 19.14 | 4.28 | 2.16 | 0.00031 |
| ALDH1A3 | 418.74 | 93.62 | 2.16 | 0.00031 |
| BBX | 60.52 | 13.53 | 2.16 | 0.00031 |
| FAM46C | 1.66 | 0.37 | 2.16 | 0.02864 |
| ZNF221 | 3.44 | 0.77 | 2.16 | 0.01274 |
| G3BP2 | 98.55 | 22.07 | 2.16 | 0.00031 |
| PTPLB | 25.68 | 5.75 | 2.16 | 0.00081 |
| BNIP3L | 166.18 | 37.28 | 2.16 | 0.00031 |
| NPFFR2 | 21.59 | 4.85 | 2.16 | 0.00031 |
| SPATA5 | 21.13 | 4.75 | 2.15 | 0.00031 |
| KRCC1 | 9.48 | 2.13 | 2.15 | 0.00453 |
| CHD2 | 87.89 | 19.80 | 2.15 | 0.00031 |
| ZC3H6 | 3.00 | 0.68 | 2.15 | 0.00031 |
| ZNF726 | 2.45 | 0.55 | 2.15 | 0.03645 |
| ARL4A | 14.97 | 3.38 | 2.15 | 0.00031 |
| TGFB2 | 2.77 | 0.63 | 2.15 | 0.00203 |
| PIM2 | 46.96 | 10.63 | 2.14 | 0.00031 |
| EIF2S1 | 185.82 | 42.08 | 2.14 | 0.00031 |
| ZNF764 | 8.25 | 1.87 | 2.14 | 0.00124 |
| TAPBP | 183.50 | 41.66 | 2.14 | 0.00031 |
| RUFY2 | 19.21 | 4.36 | 2.14 | 0.00031 |
| SLC30A6 | 31.19 | 7.08 | 2.14 | 0.00031 |
| JMY | 37.69 | 8.57 | 2.14 | 0.00031 |
| SYT15 | 7.52 | 1.71 | 2.14 | 0.00453 |
| RAB11FIP2 | 21.45 | 4.88 | 2.14 | 0.00031 |
| ZNF783 | 3.93 | 0.90 | 2.13 | 0.00164 |
| SUZ12 | 78.32 | 17.87 | 2.13 | 0.00031 |
| PKI55 | 4.13 | 0.95 | 2.13 | 0.00812 |
| HSD17B11 | 29.04 | 6.64 | 2.13 | 0.00031 |
| TJP1 | 64.62 | 14.79 | 2.13 | 0.00031 |
| ARSK | 6.53 | 1.49 | 2.13 | 0.00103 |
| CAAP1 | 14.68 | 3.36 | 2.13 | 0.00031 |
| TXNRD1 | 869.98 | 199.34 | 2.13 | 0.00031 |
| DSC2 | 17.24 | 3.95 | 2.13 | 0.00031 |
| SMAD4 | 29.84 | 6.84 | 2.13 | 0.00031 |
| TLR3 | 9.02 | 2.07 | 2.12 | 0.00031 |
| ZCCHC7 | 46.05 | 10.58 | 2.12 | 0.00031 |
| SLC12A2 | 32.68 | 7.51 | 2.12 | 0.00031 |
| IPMK | 19.43 | 4.47 | 2.12 | 0.00031 |
| PPAT | 29.73 | 6.84 | 2.12 | 0.00031 |
| MDM4 | 37.03 | 8.54 | 2.12 | 0.00031 |
| SLC35F2 | 199.34 | 45.97 | 2.12 | 0.00031 |
| TMA16 | 58.60 | 13.51 | 2.12 | 0.00031 |
| KCNAB1 | 1.90 | 0.44 | 2.12 | 0.01223 |
| APPBP2 | 26.24 | 6.06 | 2.12 | 0.00031 |
| UBL3 | 18.08 | 4.17 | 2.11 | 0.00031 |
| RDH10 | 11.82 | 2.73 | 2.11 | 0.00031 |
| FLJ45974 | 8.21 | 1.90 | 2.11 | 0.00057 |
| IL6ST | 135.95 | 31.43 | 2.11 | 0.00031 |
| PPFIBP1 | 64.71 | 14.97 | 2.11 | 0.00031 |
| PIGX | 5.77 | 1.34 | 2.11 | 0.00453 |
| SSR1 | 87.60 | 20.30 | 2.11 | 0.00031 |
| MTR | 32.55 | 7.55 | 2.11 | 0.00031 |
| DPY19L2 | 1.32 | 0.31 | 2.10 | 0.03384 |
| SBNO1 | 70.26 | 16.41 | 2.10 | 0.00031 |
| CASD1 | 9.67 | 2.27 | 2.09 | 0.00103 |
| PRKAA2 | 15.81 | 3.71 | 2.09 | 0.00031 |
| RASGEF1B | 32.63 | 7.66 | 2.09 | 0.00031 |
| C9orf64 | 19.08 | 4.49 | 2.09 | 0.00031 |
| DERL2 | 115.52 | 27.21 | 2.09 | 0.00031 |
| CHP1 | 105.91 | 24.96 | 2.09 | 0.00031 |
| KRBOX4 | 10.91 | 2.57 | 2.09 | 0.00124 |
| CCL28 | 11.58 | 2.74 | 2.08 | 0.01710 |
| ZC3HAV1 | 24.51 | 5.81 | 2.08 | 0.00031 |
| NOG | 9.52 | 2.26 | 2.07 | 0.00515 |
| CPEB4 | 19.19 | 4.56 | 2.07 | 0.00031 |
| FKBP1AP1 | 12.01 | 2.86 | 2.07 | 0.00604 |
| ITGB8 | 79.46 | 18.96 | 2.07 | 0.00031 |
| ZMAT3 | 10.50 | 2.51 | 2.07 | 0.00031 |
| MEF2C | 2.56 | 0.61 | 2.07 | 0.00291 |
| FAM169A | 13.92 | 3.33 | 2.06 | 0.00031 |
| GFPT1 | 70.34 | 16.82 | 2.06 | 0.00031 |
| VPS4B | 81.74 | 19.58 | 2.06 | 0.00031 |
| MSMO1 | 33.79 | 8.10 | 2.06 | 0.00031 |
| SEPT7 | 69.54 | 16.68 | 2.06 | 0.00031 |
| VCPIP1 | 23.48 | 5.64 | 2.06 | 0.00031 |
| UTP11L | 84.00 | 20.22 | 2.05 | 0.00031 |
| HERC2P7 | 4.80 | 1.16 | 2.05 | 0.01825 |
| ING1 | 17.41 | 4.21 | 2.05 | 0.00031 |
| SH3BP2 | 39.36 | 9.51 | 2.05 | 0.00031 |
| ITPRIPL2 | 38.42 | 9.29 | 2.05 | 0.00031 |
| G3BP1 | 378.93 | 91.82 | 2.04 | 0.00031 |
| UBN2 | 7.46 | 1.81 | 2.04 | 0.00031 |
| TTC33 | 11.73 | 2.85 | 2.04 | 0.00031 |
| SATB2 | 28.04 | 6.84 | 2.03 | 0.00031 |
| FBXO32 | 25.46 | 6.23 | 2.03 | 0.00031 |
| WASL | 33.40 | 8.17 | 2.03 | 0.00031 |
| SCAI | 4.84 | 1.18 | 2.03 | 0.00031 |
| TRHDE | 13.65 | 3.35 | 2.03 | 0.00031 |
| KLHL28 | 24.06 | 5.90 | 2.03 | 0.00031 |
| ZNF264 | 12.74 | 3.12 | 2.03 | 0.00031 |
| DNAJB4 | 14.26 | 3.51 | 2.02 | 0.00103 |
| SEPT14 | 1.87 | 0.46 | 2.02 | 0.01951 |
| ICK | 5.38 | 1.33 | 2.02 | 0.00057 |
| GOPC | 34.79 | 8.58 | 2.02 | 0.00031 |
| METTL10 | 19.82 | 4.89 | 2.02 | 0.00031 |
| MAN1A1 | 6.70 | 1.65 | 2.02 | 0.00031 |
| IDH1 | 78.31 | 19.32 | 2.02 | 0.00031 |
| ANKRD30BP3 | 3.60 | 0.89 | 2.01 | 0.04574 |
| ZNF562 | 35.77 | 8.87 | 2.01 | 0.00031 |
| ACER3 | 28.25 | 7.01 | 2.01 | 0.00031 |
| KIAA1737 | 21.85 | 5.43 | 2.01 | 0.00031 |
| INHBA | 332.62 | 82.63 | 2.01 | 0.00031 |
| AFF1 | 57.37 | 14.27 | 2.01 | 0.00031 |
| RRS1 | 97.08 | 24.15 | 2.01 | 0.00031 |
| AK4 | 30.19 | 7.51 | 2.01 | 0.00031 |
| BHLHB9 | 12.05 | 3.00 | 2.01 | 0.00031 |
| CEP57L1 | 12.78 | 3.18 | 2.01 | 0.00081 |
| RAB27B | 13.97 | 3.48 | 2.01 | 0.00031 |
| BAG5 | 39.39 | 9.82 | 2.00 | 0.00031 |
| MKX | 27.27 | 6.80 | 2.00 | 0.00031 |
| B3GNT7 | 20.13 | 5.03 | 2.00 | 0.00031 |
| SRSF1 | 119.56 | 29.86 | 2.00 | 0.00031 |
| TMEM123 | 171.97 | 42.97 | 2.00 | 0.00031 |
