# Supplementary material for: Identification of tumorigenesis-related mRNAs associated with RNA-binding protein HuR in thyroid cancer cells
Source: Oncotarget. 2016 Aug 12;7(39):63388–407. doi: 10.18632/oncotarget.11255 (PMC5325372; doi:10.18632/oncotarget.11255)
Supplement: Supplementary file 7 [file oncotarget-07-63388-s007.docx]

*Supplementary Table 6. Tumor specific HuR interaction target RNA*

|  | BCPAP | | | | K1 | | | | TPC1 | | | |
| --- | --- | --- | --- | --- | --- | --- | --- | --- | --- | --- | --- | --- |
| Gene | **HuR FPKM** | **IgG FPKM** | **Fold Change (log2)** | **q value** | **HuR FPKM** | **IgG FPKM** | **Fold Change (log2)** | **q value** | **HuR FPKM** | **IgG FPKM** | **Fold Change (log2)** | **q value** |
| ADAM10 | 98.34 | 17.00 | 2.53 | 0.00031 | 130.60 | 18.66 | 2.81 | 0.00028 | 222.49 | 40.60 | 2.45 | 0.00073 |
| AFF1 | 57.37 | 14.27 | 2.01 | 0.00031 | 92.42 | 1.62 | 5.84 | 0.00028 | 79.20 | 17.26 | 2.20 | 0.00073 |
| ARAP2 | 8.46 | 1.87 | 2.18 | 0.00031 | 2.24 | 0.40 | 2.50 | 0.00735 | 5.76 | 1.28 | 2.17 | 0.01503 |
| AXL | 789.79 | 153.33 | 2.36 | 0.00031 | 687.14 | 34.51 | 4.32 | 0.00028 | 491.89 | 38.17 | 3.69 | 0.00073 |
| B4GALT6 | 40.48 | 8.74 | 2.21 | 0.00031 | 36.81 | 4.44 | 3.05 | 0.00028 | 12.23 | 1.54 | 2.99 | 0.01440 |
| BCL11A | 7.44 | 1.64 | 2.18 | 0.00391 | 19.73 | 1.76 | 3.49 | 0.00099 | 0.98 | 0.00 | Infinite | 0.00073 |
| C2CD2 | 23.29 | 4.66 | 2.32 | 0.00031 | 19.62 | 2.08 | 3.23 | 0.00028 | 19.52 | 4.72 | 2.05 | 0.02369 |
| CADPS2 | 19.14 | 4.26 | 2.17 | 0.00031 | 49.60 | 5.66 | 3.13 | 0.00028 | 15.43 | 2.14 | 2.85 | 0.00428 |
| CDS2 | 24.44 | 5.33 | 2.20 | 0.00031 | 30.06 | 2.61 | 3.53 | 0.00028 | 11.98 | 1.49 | 3.01 | 0.00073 |
| CEP41 | 8.86 | 1.70 | 2.38 | 0.00031 | 31.31 | 3.10 | 3.33 | 0.00028 | 9.73 | 2.40 | 2.02 | 0.04092 |
| CPEB4 | 19.19 | 4.56 | 2.07 | 0.00031 | 33.52 | 6.31 | 2.41 | 0.00028 | 9.18 | 2.24 | 2.04 | 0.01299 |
| CRLF3 | 47.58 | 7.20 | 2.72 | 0.00031 | 19.95 | 2.80 | 2.83 | 0.00028 | 20.66 | 4.85 | 2.09 | 0.04702 |
| CTDSPL2 | 38.51 | 7.35 | 2.39 | 0.00031 | 32.79 | 4.06 | 3.01 | 0.00028 | 22.91 | 3.62 | 2.66 | 0.01018 |
| CUL5 | 52.10 | 10.80 | 2.27 | 0.00031 | 36.37 | 8.91 | 2.03 | 0.00028 | 37.82 | 6.98 | 2.44 | 0.00073 |
| DCUN1D4 | 35.41 | 7.47 | 2.24 | 0.00031 | 67.60 | 5.12 | 3.72 | 0.00028 | 33.01 | 6.97 | 2.24 | 0.00190 |
| DDX17 | 284.92 | 51.38 | 2.47 | 0.00031 | 886.25 | 77.53 | 3.51 | 0.00028 | 230.06 | 45.35 | 2.34 | 0.00073 |
| DENND6A | 36.67 | 7.73 | 2.25 | 0.00031 | 41.34 | 4.37 | 3.24 | 0.00028 | 47.62 | 7.54 | 2.66 | 0.00073 |
| DNAJB4 | 14.26 | 3.51 | 2.02 | 0.00103 | 10.09 | 1.43 | 2.82 | 0.00226 | 24.49 | 3.40 | 2.85 | 0.00954 |
| DTX3L | 62.74 | 11.09 | 2.50 | 0.00031 | 41.01 | 3.20 | 3.68 | 0.00028 | 31.42 | 4.00 | 2.97 | 0.00240 |
| DYNC1LI2 | 67.75 | 12.75 | 2.41 | 0.00031 | 91.09 | 17.39 | 2.39 | 0.00028 | 82.10 | 10.71 | 2.94 | 0.00073 |
| EDEM3 | 56.78 | 10.55 | 2.43 | 0.00031 | 76.68 | 8.11 | 3.24 | 0.00028 | 38.70 | 8.03 | 2.27 | 0.00073 |
| EIF2S1 | 185.82 | 42.08 | 2.14 | 0.00031 | 152.06 | 22.35 | 2.77 | 0.00028 | 100.75 | 16.87 | 2.58 | 0.00073 |
| EREG | 277.96 | 43.78 | 2.67 | 0.00031 | 29.77 | 3.88 | 2.94 | 0.00028 | 243.24 | 43.02 | 2.50 | 0.00073 |
| EXPH5 | 12.26 | 1.08 | 3.50 | 0.00031 | 7.65 | 0.55 | 3.79 | 0.00028 | 6.12 | 0.65 | 3.23 | 0.00710 |
| EZR | 260.89 | 42.12 | 2.63 | 0.00031 | 153.88 | 17.26 | 3.16 | 0.00028 | 298.11 | 40.60 | 2.88 | 0.00073 |
| FAM111A | 55.76 | 9.72 | 2.52 | 0.00031 | 56.08 | 4.07 | 3.78 | 0.00028 | 32.73 | 3.15 | 3.38 | 0.00073 |
| FAM169A | 13.92 | 3.33 | 2.06 | 0.00031 | 7.00 | 1.08 | 2.70 | 0.00028 | 11.18 | 2.67 | 2.06 | 0.01560 |
| FAM199X | 45.40 | 8.76 | 2.37 | 0.00031 | 30.09 | 5.41 | 2.47 | 0.00028 | 37.44 | 7.46 | 2.33 | 0.00073 |
| FGF5 | 18.52 | 2.96 | 2.65 | 0.00031 | 147.19 | 7.81 | 4.24 | 0.00028 | 28.07 | 2.74 | 3.36 | 0.00631 |
| FKBP1AP1 | 12.01 | 2.86 | 2.07 | 0.00604 | 48.47 | 2.08 | 4.54 | 0.00028 | 35.02 | 2.41 | 3.86 | 0.03428 |
| FNIP1 | 105.40 | 19.91 | 2.40 | 0.00031 | 107.34 | 8.70 | 3.63 | 0.00028 | 47.23 | 7.94 | 2.57 | 0.00073 |
| FOXN2 | 30.33 | 4.01 | 2.92 | 0.00031 | 41.52 | 6.14 | 2.76 | 0.00028 | 30.92 | 4.62 | 2.74 | 0.00073 |
| G3BP1 | 378.93 | 91.82 | 2.04 | 0.00031 | 492.03 | 41.79 | 3.56 | 0.00028 | 178.11 | 26.61 | 2.74 | 0.00073 |
| G3BP2 | 98.55 | 22.07 | 2.16 | 0.00031 | 132.78 | 26.50 | 2.33 | 0.00028 | 88.84 | 20.65 | 2.11 | 0.00073 |
| GAN | 20.64 | 3.72 | 2.47 | 0.00031 | 28.31 | 4.02 | 2.82 | 0.00028 | 8.56 | 1.64 | 2.38 | 0.03748 |
| GAPVD1 | 55.46 | 10.79 | 2.36 | 0.00031 | 56.86 | 5.22 | 3.44 | 0.00028 | 10.79 | 1.51 | 2.83 | 0.02737 |
| GFPT1 | 70.34 | 16.82 | 2.06 | 0.00031 | 130.41 | 15.74 | 3.05 | 0.00028 | 54.17 | 11.84 | 2.19 | 0.00073 |
| GLS | 90.16 | 14.14 | 2.67 | 0.00031 | 67.02 | 7.54 | 3.15 | 0.00028 | 241.90 | 25.83 | 3.23 | 0.00073 |
| GRIPAP1 | 97.90 | 10.75 | 3.19 | 0.00031 | 131.40 | 20.78 | 2.66 | 0.00028 | 25.16 | 0.35 | 6.19 | 0.00073 |
| GTPBP10 | 10.85 | 2.00 | 2.44 | 0.00031 | 12.88 | 1.79 | 2.85 | 0.00028 | 26.07 | 5.23 | 2.32 | 0.00073 |
| HDAC2 | 91.02 | 16.08 | 2.50 | 0.00031 | 201.07 | 23.48 | 3.10 | 0.00028 | 426.45 | 46.91 | 3.18 | 0.00073 |
| HLTF | 40.67 | 7.19 | 2.50 | 0.00031 | 34.10 | 4.10 | 3.06 | 0.00028 | 25.21 | 2.75 | 3.20 | 0.00073 |
| HNRNPU | 276.97 | 51.39 | 2.43 | 0.00031 | 355.59 | 51.81 | 2.78 | 0.00028 | 209.68 | 39.61 | 2.40 | 0.00073 |
| HSD17B11 | 29.04 | 6.64 | 2.13 | 0.00031 | 11.10 | 0.93 | 3.58 | 0.03164 | 45.93 | 8.34 | 2.46 | 0.01078 |
| IDH1 | 78.31 | 19.32 | 2.02 | 0.00031 | 105.39 | 19.42 | 2.44 | 0.00028 | 308.99 | 64.82 | 2.25 | 0.00073 |
| IL6ST | 135.95 | 31.43 | 2.11 | 0.00031 | 185.69 | 32.13 | 2.53 | 0.00028 | 122.58 | 29.09 | 2.07 | 0.00073 |
| ING1 | 17.41 | 4.21 | 2.05 | 0.00031 | 70.70 | 6.95 | 3.35 | 0.00028 | 68.42 | 6.45 | 3.41 | 0.00710 |
| INHBA | 332.62 | 82.63 | 2.01 | 0.00031 | 1617.64 | 162.73 | 3.31 | 0.00028 | 72.47 | 7.20 | 3.33 | 0.00190 |
| IRGQ | 36.03 | 5.51 | 2.71 | 0.00031 | 63.41 | 2.58 | 4.62 | 0.00028 | 8.88 | 0.42 | 4.39 | 0.00747 |
| ITGB1 | 801.28 | 118.27 | 2.76 | 0.00031 | 3393.72 | 201.59 | 4.07 | 0.00028 | 2721.36 | 322.71 | 3.08 | 0.00073 |
| JRKL | 26.53 | 5.68 | 2.22 | 0.00031 | 26.94 | 6.48 | 2.06 | 0.00028 | 20.48 | 4.74 | 2.11 | 0.03389 |
| KIF3A | 23.00 | 3.71 | 2.63 | 0.00031 | 54.17 | 7.53 | 2.85 | 0.00028 | 25.97 | 5.32 | 2.29 | 0.00073 |
| KLHL11 | 51.23 | 10.62 | 2.27 | 0.00031 | 46.30 | 6.80 | 2.77 | 0.00028 | 18.12 | 0.84 | 4.42 | 0.00337 |
| LBR | 37.09 | 7.82 | 2.25 | 0.00031 | 50.24 | 6.43 | 2.97 | 0.00028 | 150.18 | 18.90 | 2.99 | 0.00073 |
| LOC441155 | 11.30 | 1.45 | 2.96 | 0.00031 | 46.85 | 5.74 | 3.03 | 0.00028 | 8.52 | 0.08 | 6.76 | 0.00710 |
| LOC646719 | 17.05 | 2.88 | 2.57 | 0.00031 | 8.58 | 0.63 | 3.77 | 0.00028 | 6.12 | 0.51 | 3.58 | 0.01785 |
| MAP3K1 | 11.83 | 2.54 | 2.22 | 0.00031 | 10.31 | 0.82 | 3.65 | 0.00028 | 13.52 | 3.36 | 2.01 | 0.01864 |
| MTX3 | 52.26 | 10.36 | 2.33 | 0.00031 | 24.52 | 2.31 | 3.41 | 0.00028 | 12.50 | 2.58 | 2.28 | 0.00517 |
| MYEF2 | 13.79 | 2.95 | 2.22 | 0.00031 | 35.25 | 5.07 | 2.80 | 0.00028 | 65.18 | 14.28 | 2.19 | 0.00073 |
| NAA15 | 79.97 | 14.54 | 2.46 | 0.00031 | 53.25 | 9.84 | 2.44 | 0.00028 | 29.82 | 6.59 | 2.18 | 0.01268 |
| NFIB | 5.32 | 0.91 | 2.54 | 0.00031 | 117.35 | 16.31 | 2.85 | 0.00028 | 155.83 | 25.54 | 2.61 | 0.00073 |
| PCMTD1 | 27.53 | 4.63 | 2.57 | 0.00031 | 104.10 | 14.64 | 2.83 | 0.00028 | 66.85 | 12.98 | 2.36 | 0.00073 |
| PHACTR2 | 23.33 | 4.06 | 2.52 | 0.00031 | 16.27 | 2.21 | 2.88 | 0.00028 | 49.32 | 8.59 | 2.52 | 0.00073 |
| PHF6 | 113.24 | 17.08 | 2.73 | 0.00031 | 66.97 | 5.04 | 3.73 | 0.00028 | 42.81 | 7.62 | 2.49 | 0.02070 |
| PKN2 | 59.60 | 11.74 | 2.34 | 0.00031 | 21.74 | 3.76 | 2.53 | 0.00028 | 21.94 | 5.14 | 2.09 | 0.00133 |
| PPP1R12A | 117.85 | 24.54 | 2.26 | 0.00031 | 96.14 | 16.41 | 2.55 | 0.00028 | 90.03 | 18.33 | 2.30 | 0.00073 |
| PPP1R15B | 123.73 | 22.43 | 2.46 | 0.00031 | 107.21 | 8.02 | 3.74 | 0.00028 | 31.57 | 6.77 | 2.22 | 0.00592 |
| PRKAR1A | 489.34 | 82.34 | 2.57 | 0.00031 | 655.06 | 72.42 | 3.18 | 0.00028 | 180.57 | 42.11 | 2.10 | 0.01078 |
| PRKCI | 81.40 | 17.71 | 2.20 | 0.00031 | 72.96 | 10.67 | 2.77 | 0.00028 | 105.18 | 16.78 | 2.65 | 0.00073 |
| PRPS2 | 89.72 | 19.36 | 2.21 | 0.00031 | 21.25 | 4.62 | 2.20 | 0.00028 | 36.15 | 8.75 | 2.05 | 0.01389 |
| PUS7L | 42.97 | 5.41 | 2.99 | 0.00031 | 9.85 | 2.37 | 2.06 | 0.00028 | 16.86 | 1.86 | 3.18 | 0.01414 |
| RAB11FIP2 | 21.45 | 4.88 | 2.14 | 0.00031 | 16.95 | 2.97 | 2.51 | 0.00028 | 15.78 | 1.75 | 3.17 | 0.00133 |
| RDH10 | 11.82 | 2.73 | 2.11 | 0.00031 | 202.52 | 18.36 | 3.46 | 0.00028 | 4.29 | 0.24 | 4.16 | 0.01173 |
| RDX | 141.03 | 28.66 | 2.30 | 0.00031 | 457.44 | 17.63 | 4.70 | 0.00028 | 220.32 | 37.36 | 2.56 | 0.00073 |
| REST | 77.02 | 9.42 | 3.03 | 0.00031 | 105.24 | 9.46 | 3.48 | 0.00028 | 45.42 | 6.46 | 2.81 | 0.00073 |
| RYK | 118.94 | 21.40 | 2.47 | 0.00031 | 51.34 | 8.31 | 2.63 | 0.00028 | 42.62 | 3.62 | 3.56 | 0.00190 |
| SATB2 | 28.04 | 6.84 | 2.03 | 0.00031 | 110.30 | 6.68 | 4.05 | 0.00028 | 33.54 | 4.77 | 2.81 | 0.00073 |
| SAYSD1 | 9.85 | 2.04 | 2.27 | 0.00103 | 17.92 | 1.13 | 3.99 | 0.00028 | 18.57 | 3.83 | 2.28 | 0.03605 |
| SLC30A6 | 31.19 | 7.08 | 2.14 | 0.00031 | 21.26 | 3.41 | 2.64 | 0.00028 | 20.49 | 3.94 | 2.38 | 0.00133 |
| SLC35F2 | 199.34 | 45.97 | 2.12 | 0.00031 | 62.06 | 6.10 | 3.35 | 0.00028 | 62.10 | 13.68 | 2.18 | 0.00592 |
| SLC41A2 | 7.86 | 1.27 | 2.63 | 0.00031 | 18.78 | 0.86 | 4.44 | 0.00028 | 11.88 | 1.56 | 2.93 | 0.01362 |
| SLC9A7 | 48.47 | 9.81 | 2.30 | 0.00031 | 37.17 | 4.73 | 2.97 | 0.00028 | 14.84 | 1.53 | 3.27 | 0.00073 |
| SOCS4 | 26.58 | 4.85 | 2.45 | 0.00031 | 49.57 | 12.31 | 2.01 | 0.00028 | 40.37 | 7.23 | 2.48 | 0.00073 |
| SP3 | 87.94 | 17.96 | 2.29 | 0.00031 | 41.71 | 3.62 | 3.53 | 0.00028 | 24.56 | 3.28 | 2.90 | 0.00288 |
| SPATA5 | 21.13 | 4.75 | 2.15 | 0.00031 | 8.50 | 1.23 | 2.79 | 0.00028 | 6.66 | 0.96 | 2.79 | 0.01813 |
| SPIN4 | 10.81 | 1.68 | 2.69 | 0.00031 | 5.04 | 0.68 | 2.88 | 0.01546 | 5.19 | 0.70 | 2.88 | 0.03979 |
| SRBD1 | 33.76 | 5.90 | 2.52 | 0.00031 | 18.68 | 2.69 | 2.79 | 0.00028 | 22.58 | 3.84 | 2.56 | 0.01503 |
| SUPV3L1 | 50.09 | 10.84 | 2.21 | 0.00031 | 39.66 | 5.42 | 2.87 | 0.00028 | 37.46 | 7.83 | 2.26 | 0.02936 |
| THBS1 | 41.29 | 6.52 | 2.66 | 0.00031 | 3.76 | 0.34 | 3.47 | 0.00099 | 164.54 | 21.83 | 2.91 | 0.00073 |
| THRAP3 | 171.03 | 30.17 | 2.50 | 0.00031 | 192.36 | 25.51 | 2.91 | 0.00028 | 73.85 | 14.95 | 2.30 | 0.00073 |
| THUMPD1 | 44.08 | 7.73 | 2.51 | 0.00031 | 62.95 | 9.28 | 2.76 | 0.00028 | 65.44 | 13.05 | 2.33 | 0.00073 |
| TM4SF1 | 143.78 | 22.98 | 2.65 | 0.00031 | 695.64 | 45.04 | 3.95 | 0.00028 | 1606.82 | 260.40 | 2.63 | 0.00073 |
| TMA16 | 58.60 | 13.51 | 2.12 | 0.00031 | 34.45 | 7.40 | 2.22 | 0.00028 | 81.70 | 19.59 | 2.06 | 0.00288 |
| TMEM209 | 50.30 | 9.32 | 2.43 | 0.00031 | 28.30 | 3.88 | 2.87 | 0.00028 | 44.63 | 6.65 | 2.75 | 0.00133 |
| TMX1 | 94.28 | 20.21 | 2.22 | 0.00031 | 53.85 | 4.90 | 3.46 | 0.00028 | 66.53 | 11.68 | 2.51 | 0.00073 |
| TMX3 | 65.80 | 12.80 | 2.36 | 0.00031 | 48.78 | 7.02 | 2.80 | 0.00028 | 120.11 | 27.82 | 2.11 | 0.00073 |
| TNRC6B | 17.66 | 3.55 | 2.31 | 0.00031 | 23.91 | 1.68 | 3.83 | 0.00028 | 5.24 | 1.18 | 2.15 | 0.01440 |
| TRPM7 | 65.94 | 12.09 | 2.45 | 0.00031 | 61.06 | 6.29 | 3.28 | 0.00028 | 53.21 | 8.06 | 2.72 | 0.00073 |
| TSPYL1 | 66.73 | 8.12 | 3.04 | 0.00031 | 145.36 | 5.05 | 4.85 | 0.00028 | 27.22 | 3.11 | 3.13 | 0.00073 |
| TTC33 | 11.73 | 2.85 | 2.04 | 0.00031 | 8.61 | 1.80 | 2.26 | 0.00028 | 11.49 | 1.32 | 3.12 | 0.01078 |
| TULP4 | 51.06 | 11.31 | 2.17 | 0.00031 | 96.84 | 10.00 | 3.28 | 0.00028 | 28.52 | 2.79 | 3.35 | 0.00073 |
| UBA6 | 246.89 | 30.64 | 3.01 | 0.00031 | 208.49 | 19.74 | 3.40 | 0.00028 | 57.21 | 6.03 | 3.25 | 0.00073 |
| UTP15 | 36.17 | 6.70 | 2.43 | 0.00031 | 53.53 | 4.00 | 3.74 | 0.00028 | 27.76 | 4.23 | 2.71 | 0.00073 |
| VCPIP1 | 23.48 | 5.64 | 2.06 | 0.00031 | 62.26 | 8.57 | 2.86 | 0.00028 | 23.75 | 4.91 | 2.27 | 0.00190 |
| VPS4B | 81.74 | 19.58 | 2.06 | 0.00031 | 28.80 | 6.55 | 2.14 | 0.00028 | 104.58 | 20.25 | 2.37 | 0.00073 |
| YOD1 | 22.11 | 4.53 | 2.29 | 0.00031 | 12.65 | 1.86 | 2.76 | 0.00028 | 7.92 | 1.67 | 2.25 | 0.01755 |
| ZBTB1 | 52.17 | 11.21 | 2.22 | 0.00031 | 36.08 | 7.66 | 2.24 | 0.00028 | 62.57 | 11.28 | 2.47 | 0.00073 |
| ZBTB10 | 14.52 | 3.03 | 2.26 | 0.00031 | 16.56 | 1.89 | 3.13 | 0.00028 | 7.57 | 1.17 | 2.69 | 0.01389 |
| ZBTB41 | 21.59 | 3.15 | 2.78 | 0.00031 | 33.89 | 3.24 | 3.39 | 0.00028 | 17.27 | 1.67 | 3.37 | 0.00073 |
| ZKSCAN3 | 3.77 | 0.78 | 2.27 | 0.00145 | 5.34 | 0.67 | 2.99 | 0.00099 | 2.29 | 0.00 | Infinite | 0.00073 |
| ZMAT3 | 10.50 | 2.51 | 2.07 | 0.00031 | 152.54 | 19.25 | 2.99 | 0.00028 | 21.16 | 4.18 | 2.34 | 0.00133 |
| ZNF460 | 93.27 | 13.49 | 2.79 | 0.00031 | 109.09 | 4.54 | 4.59 | 0.00028 | 19.01 | 1.64 | 3.53 | 0.00073 |
| ZNF562 | 35.77 | 8.87 | 2.01 | 0.00031 | 14.71 | 0.24 | 5.91 | 0.00028 | 11.25 | 2.39 | 2.23 | 0.02936 |
| ZNF805 | 25.45 | 3.32 | 2.94 | 0.00031 | 166.33 | 3.16 | 5.72 | 0.00028 | 14.85 | 2.52 | 2.56 | 0.00133 |
